# Perceptions of physical activity and technology enabled exercise interventions among people with advanced chronic kidney disease: a qualitative study

**DOI:** 10.1186/s12882-021-02591-9

**Published:** 2021-11-10

**Authors:** Mary Beth Weber, Susan Ziolkowski, Ahad Bootwala, Alan Bienvenida, Shuchi Anand, Felipe Lobelo

**Affiliations:** 1 Hubert Department of Global Health, Rollins School of Public Health, Emory University, 1518 Clifton Road, NE, MS 1518-002-7BB, Atlanta, GA USA; 2 Division of Nephrology, Stanford University School of Medicine, Stanford, USA; 3 Exercise is Medicine Global Research and Collaboration Center, Rollins School of Public Health, Emory University, Atlanta, GA USA

**Keywords:** CKD, Exercise, Type 2 Diabetes, Pre-dialysis, Physical activity

## Abstract

**Background:**

Exercise improves health outcomes and quality of life in persons with chronic kidney disease (CKD). The numbers of persons with advanced CKD meeting physical activity guidelines however is low. We undertook a qualitative study of men and women aged 36–74 from various race/ethnic populations with advanced CKD not requiring dialysis to describe their experiences and opinions around prior physical activity, motivating factors for and barriers to exercise, and perceptions of exercise-promoting technology and group-based programming designed to improve physical activity levels.

**Methods:**

Nineteen persons with advanced CKD not requiring dialysis were interviewed at two high volume nephrology clinics enriched with racial/ethnic minority patients (Emory University and Santa Clara Valley Medical Center). We used thematic analysis to identify dominant themes (*n* = 4) and subthemes (*n* = 19) around exercise experience, barriers, motivators, views, and preferences.

**Results:**

Four dominant themes and 19 subthemes were identified. The most common motivators to exercise included physical and mental health benefits, appearance, improvement in energy levels, and potential social interaction in group-based programs. Common barriers included health concerns, particularly complications related to other co-morbidities, as well as time and transportation constraints. Participants were skeptical of exercise programs solely reliant on technology.

**Conclusions:**

The use of group-based exercise programs may motivate persons with CKD to increase exercise levels, while programs entirely based on technology may be less effective.

**Supplementary Information:**

The online version contains supplementary material available at 10.1186/s12882-021-02591-9.

## Introduction

Physical activity (PA) is inversely related to mortality, quality of life, and physical functioning in persons with chronic kidney disease (CKD) [1–6]. Individuals with CKD should achieve at least 150 min/week of moderate intensity PA [7]. However, due to low exercise levels, nearly 95% of individuals starting dialysis have physical fitness levels below the 20th percentile for the general population [8].

Understanding barriers to, views of, and motivations for PA among people with CKD is key to designing acceptable and feasible interventions tailored to the needs of this diverse and highly co-morbid population. A few studies explored these topics in persons with end stage kidney disease [9–12], but less is known about exercise views among those with CKD not on dialysis. Group-based exercise programming and technology-based fitness tools (e.g., smartphone applications, wearable fitness trackers) have been shown to overcome PA barriers and improve knowledge and activity levels [13–20]. More information is needed to understand perceptions of these proven exercise program components among people with CKD not on dialysis and provide a more nuanced picture of how to design exercise programs for this population.

Herein, we report the results of a qualitative, formative investigation of participant views and experiences to inform a technology enabled, group-based, patient-centered exercise program for persons with CKD at two clinical sites. The aim of this analysis is to describe the results of the in-depth interviews, specifically participants’: 1) current and previous exercise experiences; 2) knowledge, barriers and motivators for PA; 3) views and current use of PA promotion technologies; and 4) perceptions of group-based exercise training programs. We conclude by discussing how this information can inform the design of exercise programming for people with CKD.

## Materials and methods

### Participants and recruitment

Participants were recruited from nephrology clinics at Emory University (Atlanta, GA) and Stanford University/ Santa Clara Valley Medical Center (San Jose, CA) as part of the “Exercise is Medicine” clinical research trial designed to improve PA levels in ethnic minority patients with advanced CKD (NCT #NCT03311763) [21]. The study was approved by Emory University (IRB#00099894) and Stanford University (IRB#43198) Institutional Review Boards (IRB), and all methods were performed in accordance with the relevant IRB guidelines and regulations. Participants (Table 1) were recruited for the parent study with a screening questionnaire at a nephrology clinic visit, in-clinic fliers, and provider referrals. All recruited individuals were eligible for the formative, in-depth interviews, and study coordinators invited information-rich individuals as they enrolled in the parent study. In order to capture differences in exercise experiences and views across in men and women and participants with or without diabetes status, study coordinators kept a rolling tally of participants these factors and sampled evenly from these subpopulations. These stratifications were selected because: (a) differences in physical activity are well documented by sex [22]; and (b) clinical guidelines for diabetes and prediabetes recommend exercise as part of clinical management for patients which could affect experiences of these participants [23, 24]. All approached participants agreed to be interviewed. Participants provided written informed consent before study enrollment and additional oral consent before their interview.

**Table 1:** Inclusion and exclusion criteria of study participants

Inclusion Criteria	Exclusion Criteria
Able to provide informed consent in English	Diagnosed mental health disorder
Non-wheel chair bound	Alcohol or drug abuse
Not yet on dialysis	No fixed address or contact details
Aged 30–80 years	Unstable angina or unstable arrhythmias
No plans to move during the study period	Lack of access to internet
Interested in becoming more physically active over the next six months	Other concerns stated by the patient’s nephrologist

The research team developed the semi-structured interview guide internally (Supplement 1). An initial draft of the guide was created to include questions on topics of interest for intervention development; the research team then reviewed the guides, adding relevant probes and additional questions. The guide was then pilot tested internally through practice interviews with study team members and final edits were made to ensure clarity of questions and overall interview flow. Interviews were conducted by individuals trained on qualitative methods and the study tools (male graduate research assistants with extensive research experience [Emory] and a female project coordinator who was new to qualitative interviewing [Stanford]). Interviewers had no prior interaction with interview participants. Interviews lasted 30–50 min and were conducted at the clinic or by phone based on participant request. Key topics of discussion were (1) PA experience; (2) motivating factors for exercise; (3) exercise barriers; (4) barriers to exercise-promoting technologies; and (5) feedback on planned group-based exercise programming. Interviews were audio-recorded, and field notes were added during transcription. The planned sample size was 20 interviews, 5 in each sex-diabetes strata (males with diabetes; females with diabetes; males without diabetes; and females without diabetes). The sample size was selected based on budgetary and time factors; however, there is support from the literature that a sample of this size is sufficient for reaching saturation [25]. In fact, interviews were stopped prematurely at the Stanford site, because ongoing review of the data indicated that there was already saturation of both codes and code meaning, [25] with no new themes emerging and no novel information being reported by participants. Participants were interviewed once and were not contacted to review transcripts or data interpretation.

### Analysis

Verbatim transcripts (Supplement 2) from interview audio-recordings were reviewed to create an initial codebook highlighting key deductive and inductive themes. This initial codebook was shared with co-investigators, refined, and finalized. The final codebook included 4 themes (all deductive) and 19 subthemes (a mix of inductive and deductive). The codes corresponding to the themes/subthemes were as follows: PA Experience/Current Activity, Past Activity, Apps, Advice from Doctor, Inability to Be Active, and PA for CKD; Barriers/Health, Individual, Person, Structural, and Tech; Program Components/Trainers, Logistics, and Design; and Facilitators/Motivators, Appealing, Desire, Benefits, and Outcomes. Data were coded by one investigator (MBW) and manipulated using MaxQDA (Verbi software). Data was analyzed using thematic analysis whereby thick descriptions were developed for each subtheme, noting differences and similarities across participant demographics (e.g., male vs. female, self-reported active vs. non-active, study sites, diabetic vs non-diabetic participants). Throughout the analysis, including manuscript preparation, the data ware revisited to ensure results were consistent with the textual data.

## Results

Nineteen in-depth interviews were conducted across the two sites (11 at Emory, 8 at Stanford, Table 2). Participants did not discuss race or ethnicity as factors affecting any of the topics discussed, and unless otherwise noted, results did not vary by age, sex, site, or CKD type (diabetic or nondiabetic).

**Table 2:** Demographic and Health Characteristics of Study Participants

Variable^a^	Interview Site		Total
	Emory (***n*** = 11)	Stanford (***n*** = 8)	
**Sex, Male**	4 (36%)	4 (50%)	8 (42%)
**Race/Ethnicity**			
**Asian**	0 (0%)	3 (38%)	3 (16%)
**Black/African American**	8 (73%)	1 (13%)	9 (47%)
**Hispanic**	1 (9%)	1 (13%)	2 (11%)
**White**	2 (18%)	2 (25%)	4 (21%)
**Other/Declined**	0 (0%)	1 (13%)	1 (5%)
**Age; Mean ± SD (Range)**	58.5 **±** 11.3 (36–71)	62.4 **±** 7.6 (48–74)	60.2 **±** 10.2 (36–74)
**Employment Status**			
**Employed, Full Time**	2 (18%)	3 (38%)	5 (26%)
**Employed, Part Time**	2 (18%)	1 (13%)	3 (16%)
**Retired**	6 (55%)	3 (38%)	9 (47%)
**Unemployed**	1 (9%)	1 (13%)	2 (11%)
**Diabetic CKD**^a^	3 (33%)	6 (75%)	9 (53%)
**eGFR;**^a^ **Mean ± SD (Range) ml/min/1.73 m**	34.6 **±** 10.5 (18–52)	19.3 **±** 10.3 (6–40)	26.9 **±** 12.8 (6–52)

^a^type of CKD missing for two Emory participants; Baseline eGFR missing for three Emory participants; Data presented as Number (percent) unless otherwise indicated *SD* Standard deviation, *CKD* Chronic Kidney Disease, *eGFR* Estimated glomerular filtration rate

### Physical activity experiences

Participants’ current and past activity ranged from sedentary to running several miles daily. Disease or pain limited physical functioning and independence for four participants. Most participants reported a decline in activity after age 40. Self-described “exercisers” reported more consistent activity patterns and types over time and more enjoyment with current activities.

### Perceived benefits and motivating factors for exercise

Perceived benefits (positive outcomes of exercise) and motivating factors (reasons participants are or desire to be more active) for exercise are shown in Table 3**.** Exercise helped participants manage co-morbidities (e.g., heart conditions, lupus, arthritis, and age-related stiffness), and three men believed exercise increase life expectancy. Participants reported that exercise was important for CKD; as one man shared, “Since I think the most driving factor of kidney disease is high blood pressure, I am thinking that exercise is like the best medicine that might be made for it.” Participants understood that exercise would not cure their disease; however, several participants talked about how exercise made you stronger for dialysis.

**Table 3:** Perceived benefits and motivating factors for exercise in people with chronic kidney disease

Benefits	Motivating Factors
**Physical Health**	**Improve health**
Reduction in blood pressure	**Opportunity to be social**
Heart health	**Desire to reach goals**
Musculoskeletal health	**Weight loss/ maintenance**
Pulmonary health	Physical Appearance
Lower cholesterol	Improved flexibility
Reduction in medication use	
Improvement of other co-morbidities	
Reduce fall risk	
Delay dialysis	
**Mental Health**	
Stress reduction	
Reduce depression	
Improve mood	
Aid in focus	
**Appearance**	
**Energy and Endurance**	
Improved sleep	

CKD diagnosis was not a motivating factor for any participant to start exercising; however, a few participants did later understand that their activity could be help them manage their condition. For example, one woman shared:

*When I was first diagnosed … the doctor at that time told me that … this is something that was gonna be going on the rest of my life. So, it, so I didn't think there was anything I could do to change the results of the diagnosis … . when I started walking, I didn't think about, you know, it helping kidney disease or having any effect on kidney disease.*

Furthermore, participants spoke about wanting to remain mobile and independent: “Because if you don’t walk, you won’t be able to walk pretty soon. … if I get on dialysis, I want to still be able to walk.”

For one-third of participants, reaching PA or weight goals was an important motivator. For some participants, seeing progress towards goals was internally motivating, while others responded to the feedback they received from others. For women, weight loss was discussed in terms of physical appearance (being smaller, looking more youthful, etc.), as shown in this quote: “Once I saw the weight coming off and people was giving me compliments and, and I just felt good.” For men, however weight loss was described as it related to improvements in physical function (increasing flexibility, mobility): “I believe that if I lost weight, I would be more flexible …. Because as you know getting up and down in a chair... you know just moving around.”

### Barriers to exercise

Health-related issues were the most frequently discussed barrier to being active. Participants cited multiple co-morbidities and secondary CKD complications including arrhythmia, asthma, urinary tract infections, gout, overweight/obesity, arthritis, hypertension, neuropathy, mental health, and musculoskeletal issues. Participants were sometimes reluctant to exercise or increase exertion because of fear of pain or causing “flare-ups.” Participants’ medications made them “really fatigued … I didn’t feel like going to the gym. I didn’t like to do anything.” Except in the case of advanced disease, CKD was not identified as the dominant health-related barrier to exercise. Participants who believed their chronic conditions were well-managed stated that health was no longer a barrier to being active.

Internal (self-imposed) barriers, including feeling too busy to exercise, irregular schedules, having no time scheduled for PA, and finding available options uninspiring or not appropriate (too easy or too hard), were mentioned by most participants. For retired participants, prioritization of exercise was needed, since time was no longer a barrier: “I need to … have the discipline to essentially treat exercise like it was you know, one of the more important tasks that I have to do each day.” Other participants stated that they did not like exercise or felt like it took “a lot of effort” to do it; although this was not thought to be an insurmountable obstacle.

Over half of participants mentioned external exercise barriers. Men mentioned work-related issues such as inactive office job, long hours or commutes as barriers to exercise. Although women also stated that time was a barrier, work/work hours were not specifically mentioned. A few women mentioned specific events that were making it difficult to find time for exercise (e.g., a recent move). However, most women referred to being generally busy (“I’m usually involved in other things, you know, and I’m not able to be consistent doing it.”), being too distracted by other activities (“I can waste a lot of time … .that’s one of my barriers to exercise in general”), or late nights (“Because I like the mornings [to exercise], but lot of times, if I like I have a bible study late at night.. and uh.. I go to bed late so … I wind up getting up late.”). Other barriers mentioned by one or two participants were sleep, weather, transportation, distance to facilities, and family members discouraging PA.

Although not specified as a barrier, almost all participants did not recall getting detailed advice about exercise from their physician. As one woman shared, “I don’t recall, you know, having any conversations concerning, you know, exercising.” There was some variability by study site, with Georgia participants being more likely to remember hearing something about PA from their doctor, while participants in California reported that their doctors’ advice was more nutrition and weight loss focused. With additional probing, participants mentioned getting general advice to increase PA but no information on how to implement an exercise routine (“Well, you know, honestly, I think he asked me about it, but um, no direct instructions”). There were a few positive deviants, with two participants receiving more detailed advice ranging from an exercise brochure to gym and physical therapy referrals.

### Use and barriers of exercise-promoting technology

Despite some participants expressing an eagerness or willingness to try technological solutions for increasing PA (e.g., wearable activity trackers, mobile phone health applications), few had used these tools in the past and the majority discussed technology-specific barriers. Existing applications were not tailored to them: “That’s the biggest problem with apps, there’s no true app that’s made for someone that has a disability or health issue, they’re all made for healthy people.” Several women were uncomfortable or unskilled with technology, most relying on family members to set up or use computers, tablets, or the internet. Some men resisted smartphones or other devices because of the cost or not wanting to be “a slave to technology.” Two men expressed dissatisfaction with a specific fitness application that works with a wearable device, stating that it was “poorly designed,” not intuitive, and “a little bit techy.” Two women did not like wearable devices for aesthetic reasons. Participants were not motivated to use technology long-term: “Well I had actually gotten it for, to monitor my um, heart rate and pulse and stuff, when I first um, got sick. … But um, when I finally mellowed off, it [use of technology] did too.”

### Group-based programming

Most participants recalled fondly a time they were active, with the most favorable memories typically involving social activities (athletics, dancing, running groups), and an opportunity to be more social was the second most widely discussed motivator for PA. Participants discussed how group exercise helped overcome barriers, was more fun, and improved mental health. Also, seeing others be active, particularly similar individuals, encouraged participants to do more and try different activities: “And like I said, I think other people can motivate you too … just looking at the other people.”

Participants felt that a group exercise program specifically for patients with CKD could improve general and specific health (e.g., cholesterol levels, blood pressure, glomerular filtration rate) and possibly improve or slow progression of their kidney disease, although no program could reverse kidney damage (“I’ll never be without kidney disease … it’s chronic, it’s, it doesn’t go away.”). Most participants discussed how an exercise program could help them be in better shape, increase strength and energy, improve mobility and weight, and build muscle. Half of the participants stated that the program would improve their motivation for exercise: “That is one of the one of the reasons why I want to be a part of it, because I’m hopeful … I’ll be able to just use that as... a jumping off point for you know, healthy regimens.” Participants felt the program could increase knowledge of exercise and kidney disease, and build community, especially among older people where community isolation was more common.

Participants discussed the importance of planning programs that overcame barriers to class attendance like traffic, work hours, and lack of transportation. About half of the participants were willing to try any type of exercise or activity as long it was beneficial to their health. Walking (especially outdoors), the most common response, elliptical machines, stationary bicycles, Zumba or other dance-based exercises, stretching exercises (e.g., Yoga, Barre), weight lifting, games/sports, or interval training were particularly appealing. Participants hoped instructors/exercise trainers would be skilled motivators and empathetic to their health limitations. Although participants understood that trainer should push participants a little, they would be discouraged by someone too strict and rigid (“the drill sergeant”). Trainers should be knowledgeable about CKD: “I could go to class do everything and … then two weeks later, I can come back and not be able to do anything, and most trainers don’t understand that concept because they’re trained that your supposed to make gains every single week and that does not work for people who have any type of chronic illness.”

## Discussion

**Fig. 1:**
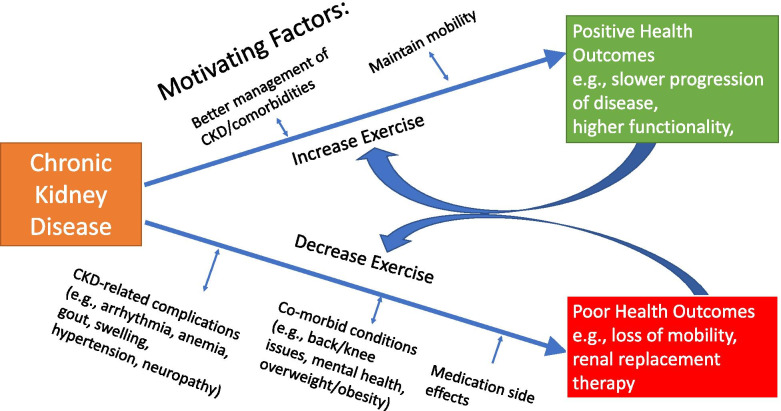
Health-related Exercise Barriers and Motivators in People with CKD and Their Interconnectivity with Health Outcomes

In a racially and ethnically diverse group of people with advanced CKD but not yet on dialysis, we found that participants expressed realistic expectations about the benefits of exercise including potential improvement to their health and physical functioning. None expected exercise to ‘cure’ existing kidney disease, but many were hoping to delay progression of CKD, maintain mobility, or better withstanding initiation of renal replacement therapy. Health-related factors were both the primary barrier and motivating factor to PA in this study (Fig. 1). These health-related factors have a cyclical relationship with both exercise and CKD, whereby health-related factors: 1. directly affect PA levels thereby affected CKD outcome for the better (increased mobility, slower disease progression) or worse (earlier need for renal replacement therapy, loss of mobility); and 2. are made worse (barriers) or better (motivating factors) by both exercise and CKD. Participants expressed the necessity of exercise programming to accommodate significant physical debility and self-efficacy limitations.

Even though all participants mentioned at least one benefit to and one motivator for exercise and most participants expressed an interest in being more active, few reported a regular activity schedule. These findings are consistent with other studies showing generally low PA levels among people with CKD [2]. The few participants reporting regular PA had very high activity levels (e.g., daily runs), which is consistent with published quantitative data showing that persons with CKD meeting PA guidelines were very active [26].

The results reported here are generally consistent with other studies. One qualitative study among individuals with CKD in the UK, reported similar barriers and motivating factors for PA; however, family support was a key motivating factor for exercise among UK participants, [27] while in this US-based study, participants were motivated by the social interactions exercise could provide. This could reflect a higher social isolation among participants in this study. In an integrative review of the literature on barriers to PA among renal disease patients, low energy level and fatigue were the most frequently cited barriers to PA [28]. This is consistent with what participants reported in this study. Although low energy was not specifically cited, health issues, multimorbidity, medication side effects, and stress-related strains on mental health, all of which can result in physical and/or mental fatigue, were cited as the most significant barrier to exercise.

Although interventions are needed in this high risk population to address low PA levels, [29] few programs have been customized to the specific challenges facing patients with CKD. Participants reported that social support was a major motivating factor for PA. Group-based exercise programs have been shown to improve cardiometobolic health [30–33], and social support could help mitigate the mental health strain of chronic disease. However, more research is needed to find successful, sustainable and acceptable group-based programs to increase PA in all persons with CKD. Existing trials in this area are limited, focusing primarily on patients on dialysis and on individual exercise [34–37]. CKD programs could benefit from the knowledge gained in proven, group-based programs used in prevention or management or other chronic conditions, where tools like goal setting, problem solving, responding to barriers and challenges, and building social support have been successfully used to improve health behaviors (for example [18, 30, 38, 39]).

Furthermore, participants discussed how existing exercise programs and mobile applications were not responsive to their needs as CKD patients, often applying general exercise advice and feedback, which was not always appropriate to their current realities. Programs, including m-health applications, are needed that take into account patients’ health needs, changing energy levels, and multi-morbidities. Participants also reported a reluctance to maintain use of technology-based exercise tools and found existing tools too complicated for those with lower computer literacy. To be successful, technology interventions must respond to the needs of the particular patient population, in this case including an easy-to-use interface, flexible advice that responds to the changing needs and abilities of CKD patients, and promotion of social support. In addition, interventions should build in tools and time to reduce the digital divide within and across their patient population by increasing self-efficacy for technology use, building trust with users, and including sufficient education efforts before asking participants to adopt new technologies [40].

Participants in this and other CKD studies [27] report a lack of specific exercise advice from their medical care providers. Understanding the barriers to and motivations for exercise in this population can help healthcare professionals guide their patients’ PA. Exercise advice should be specific (e.g., 30 min of moderate activity like walking 5 days per week), address barriers (e.g., chair exercises for when mobility is limited), and focus on motivating factors specific to different patients (e.g., physical appearance aspects of exercise for women and longer life for men).

This study has several strengths. In-depth interviews with a diverse population with CKD from two clinics in different regions of the country provides a rich description of the exercise barriers, motivating factors and experiences. Study results could be strengthened by including interviews with non-English speaking patients, who might have a very different experience with care and disease management, and data is needed on patients with early CKD and at non-academic healthcare centers. The goal of these interviews was to collect information for the development of an intervention in the target community, and the discussions focused on topics that would assist in that goal (e.g., understanding experiences and views of physical activity, identifying barriers and facilitators, and discussion planned intervention components). Because of this focus, even though the interview participants were racially and ethnically diverse, there could be additional issues related to race, ethnicity, or other demographic or sociocultural factors that were not identified.

In conclusion, this study showed there is interest in exercise programs for CKD management, provided such programs address important barriers and are responsive to the unique needs of the CKD community. Careful attention needs to be paid to the likely severe physical disability in a majority of participants, setting realistic expectations and logistical barriers. Programs should not rely solely on technology, but could integrate technology into a program that promotes social support and includes preferred motivators like goal setting and supportive instructors.

## Supplementary Information

**Additional file 1.**

**Additional file 2.**

## Data Availability

All data generated or analyzed during this study are included in this published article (please see Supplements 1 and 2 for the interview guide and de-identified verbatim transcripts, respectively).
