# Supplementary material for: Perceptions of physical activity and technology enabled exercise interventions among people with advanced chronic kidney disease: a qualitative study
Source: BMC Nephrol. 2021 Nov 10;22:373. doi: 10.1186/s12882-021-02591-9 (PMC8579645; doi:10.1186/s12882-021-02591-9)
Supplement: Supplementary file 1 — Additional file 1. [file 12882_2021_2591_MOESM1_ESM.docx]

**INTERVIEW GUIDE**

**Manuscript Title: Perceptions of physical activity and technology enabled exercise interventions among people with advanced chronic kidney disease: a qualitative study**

Authors:

Mary Beth Weber, PhD^1**^

Susan Ziolkowski, MD^3^

Ahad Bootwala, MPH^1,2^

Alan Bienvenida, MPH^1,2^

Shuchi Anand, MD^3*^

Felipe Lobelo, MD PhD^1,2^

Affiliations:

^1^ Hubert Department of Global Health, Rollins School of Public Health, Emory University, Atlanta, GA

^2^ Exercise is Medicine Global Research and Collaboration Center, Rollins School of Public Health, Emory University, Atlanta, GA

^3^ Division of Nephrology, Stanford University School of Medicine

**Corresponding Author: Emory University, 1518 Clifton Road, NE, MS 1518-002-7BB, Atlanta, GA 30322; +1-404-712-1902; mbweber@emory.edu

**For Study Staff only:** Interview Date:____ /____ /____ Location: __________________ Staff Initials:____

Please fill out the questions on the following pages. We will not share your answers with anyone else. **If anything is unclear to you, please ask a member of the study team, and we will help you.** For each question, read all the answer choices and then check the box next to the answer that best describes you or your behavior.

1. How old are you? ________________ Years
2. What is your sex?
   - Male
   - Female
   - Transgender
3. Are you of Hispanic, Latino, or Spanish origin?
   - Yes
   - No
   - Unknown
   - Prefer not to Answer
4. Which category best describes your race? (One or more categories may be marked)
   - American Indian/Alaska Native
   - Asian
   - Black or African American
   - Native Hawaiian/Other Pacific Islander
   - White
   - Some other race
   - Declined
   - Unavailable/Unknown
5. Are you currently (check one):
   - A full time student
   - Employed full-time
   - Employed part-time
   - Retired
   - Unemployed
6. Which type of mobile phone do you have?
   - I do not have a mobile phone
   - I have a mobile phone, but not a smartphone
   - iPhone 5 (released September 2012) or older
   - iPhone 5s (released September 2013) or newer
   - Android 4.3 (released 2012) or older
   - Android 4.4 and later (released 2013) or newer
7. Do you own a fitness wearable device?
   - No
   - Yes, an Apple Watch
   - Yes, a Fitbit
   - Yes, a Garmin
   - Yes, a Withings
8. If you enrolled in an exercise program for people with kidney disease, when would be the best times to come for classes (check all that apply)?
   - I would not be willing to attend a class like this
   - Weekend mornings (before noon)
   - Weekend afternoons (1-6 pm)
   - Weekend evenings (after 6 pm)
   - Weekday mornings (before noon)
   - Weekday afternoons (1-6 pm)
   - Weekday evenings (after 6 pm)

**Introduction:**

Hello, my name is XXXXX. I am working with other researchers here at Stanford and Emory University in Atlanta to understand the experiences people with kidney disease have with exercise. What you tell us today will help us to create a program to make it easier and more fun for you and other people with kidney disease to be more active.

Before we start our discussion, I want to get a go over the informed consent document with you. I want to hear your personal experiences. There are no right or wrong answers, so please be honest. Everything you say today will be kept completely confidential. To help us catch everything you say to us, we will be making an audio-recording of our discussion. This is YYYY (note taker’s name). She/he is here to help make sure I do not miss. Do you mind YYY joining us to take notes on our discussion? We anticipate that this interview will last about 60 minutes. [*Review the Informed Consent document and answer any questions the participants has.*]

We also want to learn a little more about you before we get started. Could you please fill out this short questionnaire?

Thank you! Let’s begin.

**Introductory Questions** (10 minutes)

1. Can you tell me about a time you were physically active that you really enjoyed?
2. What do you think when you hear the word exercise?

**Physical Activity/Exercise Behaviors** (30 minutes)

1. Tell me about your current exercise routine.
   1. Probes: frequency, type, location
   2. Probe: Is this your usual amount of physical activity?
   3. Probe: If participant responds he/she does NOT have an exercise routine, explore: In what ways are you able to be physically active/can you describe a week when you were active? How were you physically active this week?
   4. Probe: How did your exercise routine change when you were diagnosed with kidney disease?
2. What are some of the benefits of exercising?
   1. Probe: health related; personal benefits
   2. Probe: What motivated you to be physically active?
3. How important is exercise for patients with kidney disease?
   1. Probe: Has your doctor talked to you about exercise and kidney disease management? What has she/he told you?
   2. Probe: **What exercise advice were you given?**
4. What are some of the things that make it hard for you to exercise?
   1. Probe: health barriers; time; feeling unmotivated
   2. Probe: What would make it easier for you to overcome X barrier?

**Feedback on possible program components** (15 minutes)

1. One of the things we are considering in our exercise program is group classes. How do you feel about group exercise classes in general?
   1. Probe: What do you like/dislike about group classes
   2. Probe: how do you feel about exercise classes created especially for people with kidney disease?
   3. Probe: ask about barriers to participating – transportation, time
2. How should we design these classes to be most convenient and appealing to you?
   1. Probe: type of activities, location, time
   2. Probe:
   3. Probe: What would you value most from an exercise program?
3. How would you judge if the exercise program was successful?
   1. Probe: Individual/personal success
   2. Probe: Program success
4. What is your experience with exercise instructors?
   1. Probe: If you were attending an exercise class, what could the exercise instructor do to help you be more willing and excited about exercising? How helpful is goal setting for you when exercising?
   2. Probe: What things would discourage you from exercising?
5. We are also considering using a mobile phone app as part of our program. Can you tell me about your past or current experiences with using a health app on your mobile phone?
   1. Probe: exercise apps – which used, what liked/did not like
   2. Probe: activity tracker
   3. Probe: are you still using the app? Why or what made you stop?
   4. Probe if participant does not use apps or stopped using apps, explore why (e.g., are they intimidated by technology).

**Conclusions**

That is great. You have given a lot of very helpful information. Is there anything else that you want to share with us, talk more about, or clarify from what we discussed today?

Wonderful! Thank you so much for taking the time to talk to us today. I know that your experiences will help us to create a program that will make exercise easier and more fun for people with kidney disease.
