# Supplementary material for: Perceptions of physical activity and technology enabled exercise interventions among people with advanced chronic kidney disease: a qualitative study
Source: BMC Nephrol. 2021 Nov 10;22:373. doi: 10.1186/s12882-021-02591-9 (PMC8579645; doi:10.1186/s12882-021-02591-9)
Supplement: Supplementary file 2 — Additional file 2. [file 12882_2021_2591_MOESM2_ESM.docx]

**TRANSCRIPTS**

**Manuscript Title: Perceptions of physical activity and technology enabled exercise interventions among people with advanced chronic kidney disease: a qualitative study**

Authors:

Mary Beth Weber, PhD^1**^

Susan Ziolkowski, MD^3^

Ahad Bootwala, MPH^1,2^

Alan Bienvenida, MPH^1,2^

Shuchi Anand, MD^3*^

Felipe Lobelo, MD PhD^1,2^

Affiliations:

^1^ Hubert Department of Global Health, Rollins School of Public Health, Emory University, Atlanta, GA

^2^ Exercise is Medicine Global Research and Collaboration Center, Rollins School of Public Health, Emory University, Atlanta, GA

^3^ Division of Nephrology, Stanford University School of Medicine

**Corresponding Author: Emory University, 1518 Clifton Road, NE, MS 1518-002-7BB, Atlanta, GA 30322; +1-404-712-1902; mbweber@emory.edu

**Audio File Name: E-01 10-25-2018.mp4**

**Recording Date: 10/25/18**

**Interviewer: AB**

**Transcriber: AB**

**Dates: 1/10, 1/11 2019**

**File Name: #1 CKD Interview Emory University (10-25-2018)**

I: The first question I am going to as you in this introductory question is, can you tell me about a time that you were physically active that you really enjoyed?

P: Wow, my whole life. I mean, I've never been inactive since I was age 12.

I: So, you've been participating in physical activity?

P: I'll be 70 in two months. And I am still a competitive runner. So, I have been an athlete all my night. Up to the national level so, you know I am constantly exercising, going to the gym, yoga, or stretching, or running, or [ricing?].

I: So, you do enjoy physical activity?

P: Absolutely. That's probably why I am still alive.

I: It is important. Okay, so then we can move on to the next question which is, what do you think when you hear the word exercise?

P: Um. wow. Vitality, energy. I can eat more because I am not going to gain weight. [laughter] I mean, I love to exercise and I'm an athlete. I enjoy it. It brings a lot of joy to me.

I: Okay. Alright. So, next question is tell me about your current exercise routine.

P: Do you want to go day by day? Or a summary of the week?

I: Um, whatever you feel most comfortable. The more information you do provide us, the better information we will have to create a better program.

P: Okay, let's just go seven days a week. I can go specific day, or just kind of you overall what I do a week. You know, in a seven-day period where I am exercising probably at least 6 days a week. And, that would include... I run three to four days a week. I go to the gym, kind of varies in the warmer weather. I probably only go to the gym a couple days a week. In colder weather, I probably go to the gym maybe 4 days a week. And that would include uh, when I go to the gym, lifting weights, stationary bicycle, um, stretching, yoga type exercises. The treadmill. I may walk a lot. I do a lot of walking too, with my running. And, um you know I probably put in currently I probably put in 20-25 miles of running a week.

I: wow

P: And um, you know I don't lift heavy weights. But I do, do a lot of repetition stuff like bench presses and curls. Um... and just that type of exercise. I am not doing let's say heavy weights, but I am doing lighter weights but a lot of repetition and sets. Like, 3 sets of 20 and that type of thing.

I: Okay, so you are very active, that is what I am gathering.

P: Uh, I am probably a nut about exercising.

I: Okay, so I am guessing this is your usual amount of physical activity? Like you do this every week.

P: Yes. Yes, every week. You know, it may vary. You know it may vary in intensity of running. Like I may one week do um, if you know what interval work is, where you are doing speed work. Short distances, very fast.

[05:08.2]

P: With a short interval of rest. To peak out your heart rate. Then some days, I'm doing, like this week I'm doing a lot of longer runs, you know up to 10 miles. And I am actually racing pretty much every week too. I average about 40 races a year.

I: You must have a lot of t-shirts and medals.

P: What you want to buy some? [Laughter]

[06:00.1]

I: Alright so then, another question would be, how did your exercise routine change when you were diagnosed with kidney disease?

P: You know really, the kidney disease was kind of a byproduct if I can just regress a minute. I don't really want to brag but I was a really good athlete in high school, college, and after college. And you know, it of course went into a job and a business career and I still kept running. I kind of didn't compete for a while but I did keep up my exercise. And then, around 50, I kind of started competing again. And um, I started having some heart arrythmia’s. I thought I was in perfect health. You know, I tried to eat good, maintain my body, didn't smoke, didn't really drink or anything. So, I had arrythmias and that is how this came to fruition. You know, went to Emory and found out I had atrial fibrillation. And then, as things progressed, I found out a lot of this was caused by exposure to agent orange in Vietnam. And that is how I found out I had kidney. Yeah so, I didn't know it. I thought I was just a really healthy guy. And then that just kind of, I guess really crushed my world so to speak. You know, other than the arrythmia, the atrial fibrillation, were given me some problems exercising. I just continued on you know because, my parents were in the medical field and I just kind of knew that exercise was not the magic pill to stay healthy, but it would give me more help than harm. You know, I just kind of, took a little hiatus from competition when I had that atrial fibrillation. I had a couple of [operations] and they kind of fixed that. So, I just started competing with guys, you know, the only problem it caused me with the kidney thing is I have to watch out what I do before I go take my blood test because my [keratins?] levels go crazy if I’ve been exercising or lifting weights. So, other than that, I really don't think about it. I really don't give it a second thought. Now I have a physician at Emory, and then I go to the VA too. They are tracking my kidney test like all the time. You know, until... and they all encourage me to keep exercising. So, I don't really... I don't just say oh god, I don't think I should be running because I have kidney disease. I just keep going. Well, I know it is good for your overall health and that is the only reason why I am willing to help you in this, is that I see so many people quit exercising if they have kidney disease or heart disease or other illnesses. Even though they still could be active and they just kind of give up. And I've had a lot of friends die because I am getting at that age. Because they just basically sit down and quit exercising.

10:00]

P: So, it is very important if you can physically do it, to keep moving. And it doesn't have to be as hard as I am going.

I: Yeah, that's a great segway into our next question which is, what are some of the benefits of exercising, and so these include like health related or also like personal benefits. So, if you could elaborate on that a little bit.

P: Yeah, that's you know [inaudible], scientific benefits. I mean, your mind is clear, you have more energy. Um, I mean psychologically, you are just more alert. You are more interested in doing other things other than exercise. Going places. If you have other hobbies. My wife and I run together, we exercise together, we race together. You know, and she didn't start exercising till she was 64. And this made a tremendous difference in her life. And I mean, you just meet so many other people. I mean it is fate that we are all just a bunch of old people. But there is a big segment of the younger generation that are really into exercise and activity whether it is running, or swimming, or um, tennis, or golf, you know, whatever. I am amazed by the number of older people who are very active... It makes your appetite better. Your whole senses, your whole body, you are just more in tune to everything.

I: Agreed. And so, I am guessing these benefits have motivated you to continue to be physically active. Is that correct?

P: Absolutely. I used to take a day or two off. I take at least one day off a week. Sometimes two. And if I take like two days off together, about the second day, I feel like sluggish. You know, I just kind of don't have the energy [inaudible] So, that's why I probably make a point everyday of being... you know there is a lot of days I go run 6 to 7 miles and I come home and walk and work out in the yard, for 5 or 6 hours. I mean I stay, I don't just come home and sit and watch tv.

I: Okay. Alright, that was great. A lot of good information there. We’re going to move on to the next question. And so, this question is kind of talking about the same thing and you kind of touched upon it a little bit. It is, how important is exercise for patients with kidney disease?

P: I mean, I think kidney disease is so related to everything else, hypertension, heart disease, um. I guess the bad thing about kidney disease, from my experience and most people I know, you don't really know. You know, if you hadn’t had a blood test or if you hadn't had a physical, you really don't know that you had kidney disease until like it's bad. And, I'm not sure a lot of people know they have kidney disease. Because a lot of guys don't want to go to the doctor, you know until they are like [close to the end]. I am very in tune with my help. I won't hesitate. I don't know, I just think that if your body is fit, your blood flow is better, your organs are more healthier. Obviously, you don't, if your exercising hopefully your weight is under control. You know, you don't have layers of fat squeezing your organs. [15:00]

I: Yeah, those are all really important.

P: From the scientific standpoint, I don't really know. From the scientific, I know exercising the heart, but I am not really sure about the kidneys other than exercise helps you keep your blood pressure lower. You know you got better circulation if your blood and all that jazz.

I: And so, has your doctor talked to you about exercise and kidney disease management? Like directly? So, when you were diagnosed, did they offer you any exercise advice?

P: Not really. But to be honest to you, everybody I see at Emory and I see 3 different doctors there, they all know I am a runner. So, they kind of well, I mean I don't. They know I am a very serious runner. I guess they don't feel like they need to tell me because they probably feel like I can tell them. I mean, that may sound kind of goofy, but I make it well known that you know I exercise a lot and I know the importance of my total health as it is related to activity.

I: Okay. Alright and so, moving on to the next question, so I know from what you have told me so far, that exercise comes very easy to you.

P: Yes. Well, it doesn't come easy now. You know I can give you 10 excuses why on a day when it is 21 degrees, I don't really want to go running. [laughter] You have to make an effort to do it. It isn't like eating ice cream where you can just sit down and enjoy it. You know, you got to go. I am getting ready to run a 10-mile race Saturday and I can be honest with you at 3 o'clock Saturday morning when I am getting out of bed, that is not easy. [laughter]

I: Yeah, I can only imagine.

P: But I know, the benefits outweigh the discomfort.

I: And so, we are going to delve into some of those discomforts and barriers just for people in the future, deal with these barriers more. Times when you do find it hard for you to exercise, what are those barriers or those factors that affect whether or not you want to exercise or your motivation to exercise.

P: Well, I mean, at my age. It should be any age. As a friend and I were discussing yesterday in fact. When you are 30, you think you are going to live forever. I don't know how old you are. But if you are young, you might not, you may not give death or your health a lot of thought. Because you feel good all the time and have plenty of energy. Yadda Yadda yo. But when you start getting older, if you are positive about life, that you really enjoy life, you kind of want to hang around a little bit longer. And being healthy, you know, that is the ticket. It is not the guarantee, but it is a pretty good ticket. And, I am not going to say, I mean there is a lot of important things in my life, but my health is probably number one. Because as I always tell my wife, if I am not healthy, I can’t take care of her. So, I got to be healthy to take care of myself. She don't need taking care of, but you know what I mean. You can't do other things if you are not healthy. So, when I get up in the morning and it's raining and snow... it’s cold and miserable. I just say I am going to the gym and unless it’s an emergency of some type, that's my first priority every day. And, like say I can come up with a lot of excuses. Maybe one of my friends calls me and says, let’s go to lunch. You know, doing my exercise is what is most important because my health, good health, [20:00] allows me to enjoy other things. So, my recommendation to anybody is, it is easy to sit on the couch, eat a donut, drink coffee, and watch tv. That's easy. But it is only easy for a short tv for a short period of time because when your health gets so bad, you won't be able to do that. And, you know you don't have to be, go out and do all the things that I do. You can go to the mall and walk around the mall if it’s cold and you don't want to go to the gym. My mom use to do that all the time. She went to the mall and would walk several miles every day. I mean, you can do things like that. You can go to yoga. My wife goes to yoga almost every Monday through Friday every day. And um, there is just a lot of different things you can do that are enjoyable, that are social. Do you know what pickleball is? Yeah, I mean we have a big group of people here who play pickleball. It is a very social event, but you get exercise. The yoga class that we go to you know, there are 20 to 30 people in those classes you know. It's not just about sweat and stinking, it's a social activity too. It can be enjoyable, and you can look forward to seeing you friends.

I: That's a great segway into our next question. And so, one of the things that we are considering for this exercise program is group classes. And so, how do you feel group exercise classes in general?

P: 100% support them.

I: What do you like or dislike about them?

P: There is no, really, you know other than you may run into a personality that you may have a conflict with because you generally not at all cases you can control who is in that group. But it is so much positive because there is so much positive reinforcement. If everybody in the group has the same goal, um, you know, where there is to be a good runner. We got a little running group up here that meets a couple times a week. We go all run and after we run we go grab some coffee or whatever. And you know, we will sit around after we run. We may run 10 miles, 6 miles or whatever. And then we sit around for an hour and a half. Talking about different things. Politics, telling fish stories about how good we were when we were young. I mean it's just, helps you the days where you don't really feel like going but you know your friends are waiting on you. And you all are going to accomplish something together. You know, it is a really great motivation. To get out of bed. Sometimes you have to get up early to go meet these people. And I definitely. When you run by yourself sometimes and I do do that sometimes, it is much more easier to succumb to reasons not to do it. I need to cut the grass or I need to do laundry, or I may... blah blah blah. But if you got other people that you know that are waiting on you, but I wouldn't say depend on you but, you know that you interact with that's more of a motivational thing. And you feel like you are accomplishing goals together.

I: Yeah, definitely that goal portion is very very helpful. And so, um, let's see.

P: And I just want to add one thing. And the goal doesn’t have to mean that you are going to run your fastest 5K time, but it could be that you lose one pound, or you want to do whatever. You want to learn a new yoga pose. You know, whatever. Everybody has got different goals, but you know, you all work together, you know in our little group, we never leave anybody behind. If we are running, everybody stays together. You know if one is faster than the other, we just kind of slow down a little and pull them up with us. Okay. I talk a lot.

I: No, it's great. You are giving us a lot of great information, thank you. So, for these exercise classes, these group exercise classes, [25:00] how do you feel, how do you feel people with kidney, how do you think they would react. Do you think it would be something that would be feasible? Do you think there are possibly any barriers for people to participate specifically for group classes? And since we already know that you are very adamant about group classes. So, for people who may not be as active, do you think they would benefit from these group classes.

P: Oh sure! You know. To be blunt. If you are getting somebody off their ass just to do something. Whether it is walking or getting on a stationary bike. Or doing yoga, or stretching, or Zumba, or whatever. I mean, no matter what their ability is, if you just getting them out of the house. I mean, you've got, they’re going to have a brighter outlook on life. They are going to be mentally stimulated as well as physically stimulated. You know, I mean, I go to the gym with people, you know and were all different levels. I might be over on the treadmill doing a hard exercise uh, [inaudible], they may be walking but you know, they are getting benefits too. Um, you know I don't know scientifically at what point with kidney disease do you get into um, dialysis or something. I mean, I don't know. I am not at that stage, so I don't know how that would change me. I am sure it would dramatically. So, I just think that where I am in my kidney disease is that exercise is very beneficial. You know, it's just, I don't know. Usually I think, that kidney disease is usually out of some other, disease or condition that probably goes along with that. Since I think the most driving factor of kidney disease is high blood pressure, I am thinking that exercise is like the best medicine that might be made for it.

I: Yeah, I think that is the whole point of our study, so I am glad you resonate with that. And so, another question would be, if we were to create these classes, these group classes, in your opinion, what would be most convenient and appealing to you in the way that we design them? So, specifically like what kind of activities would you be willing to participate in as a group that you think would be appealing to a large group of people?

P: Um, I mean I would be interested in any level. You know, it's me. You know, I got a lick that I am kind of abnormal, for my exercise. So, you know something that would involve walking, or stationary bike type thing, cardio. Some cardio work. Maybe, doesn’t have to be rigorous but something that gets your blood flowing and stimulates your senses. Probably a lot of stretching, yoga type things. Um, because that is the big thing with older people. As you get older, you get stiffer. Everything hurts, your knees, your ankles, your hips, your shoulders, whatever. And most of that is caused by atrophy of not using your muscles. So, I think, if I were doing a program for, and really it would have to be age related but, I would try to start people off with exercising, stretching, yoga, light weights, and kind of get them and maybe do that for a few weeks and then get into um, whether it be walking or [30:00] stationary bikes. you know stationary bikes are good because it is a low impact type situation. Yeah, and meditation. My wife says meditation because she says she’s a big yoga person. You know, that, you got a get people to tune back to what is important and it is their body. Because like, if you care what is going on in your body. And I hope you are not fat, I don't want to hurt your feelings. But if you're 400 pounds, you are not doing much of anything but eating. You're not 400 pounds, are you?

I: [laughter] I am not. I am actually very skinny.

P: But uh, you're thin?

I: Yeah, I would say so.

P: Okay, that's good. Well, I don't want to hurt your feelings.

I: Well, I appreciate that.

P: You know, well I go to the doctor’s office, and I got, when I, if I go to my cardiologist and my nephrologist and you are out in the waiting room and you are like, at least 70% of the people are like overweight, you are going like, no wonder you are here. You know, I am not trying to be condescending because I am there too, but I know that it can happen to healthy, slender people, but the key is you got to be in some kind of shape.

I: Yeah, definitely. And so, moving on from there, the next question would be...

P: One more thing on that, you got to start off slow. Cause, if you hit the road running so to speak, a lot of people will get scared off. Yeah, you got to start slow, get people kind of see where you are going with this. focus on the stretching and some easy stuff and just let them build up from that.

I: In your opinion, what would be a right a length of time for an exercise class or group class to the point where you feel like, um, you exercised enough. Like what would that range be like for you?

P: 30 minutes to an hour. I mean that wouldn't be good to be, but for the average person, 30 minutes to an hour. I mean I sit through 4 hours a day sometimes. But, usually the problem with 30 minutes is by the time you get ready to exercise and your kind of cool down, you've only done about 10 minutes. You know, an hour is really good because, you know how people are, they talk a lot, socialize.

I: Okay

P: They try to find an excuse not to sweat [laughter]

I: Yeah, I am guilty of that sometimes.

P: Yeah, we all are. I mean, I go to the gym and I see these guys that are monster muscle guys, and they are there for a while but they really only exercise for 10 minutes because they are talking too much. I mean when I go to the gym, it is all business. I mean, I say hi to people, but you know, I really don't stand around and talk. I'm there, I am on a mission. I come in, and when I start, I don't stop till I am getting ready to walk out the door. And that is just me because I am going like, I am not wasting, I've got so much to do in my day and I don't want to waste a lot of time.

I: Alright, so moving on, the next question is, how would you judge if the exercise program was successful?

P: Well, I don't know. My kidney disease, because we are strictly talking about kidney disease, is been stable for 20 years now. Uh, you know, I've had some ups and downs with it but generally speaking, it has been very stable. And I do believe that exercise has been the main reason. Because I kept my blood pressure stable too. And um, like say now, I have some instance during that 20 years where I've had some health issues pop up. Some of them are my fault probably but some of them not my fault. I mean, I had a bout with sepsis last year. [35:00] It wasn't my fault. You know I had a little foot injury and the doctor, whether he intentionally misdiagnosed it to try to gain more financial success from me, or he was incompetent, he let a very minor foot issue flair up into sepsis and, talk about kidney disease, my kidneys almost shut down. So, you know, I had to rebound from that and I have. I have gotten my kidneys back. They are not normal I mean, I mean obviously I got chronic kidney disease, so I am not normal. But I got them back to where I was, and I just do think because I am very cognizant of my blood pressure, I take it every day. I watch what I eat. I am not a perfect eater but we very very, put great effort into doing it. I just need my exercise to keep my weight in check. It has kept my blood pressure down. And I, because there is no magic pill that you can take for kidney disease, I just believe that is what has kept me stable. And, Dr. [name] is my nephrologist at Emory and she has gone from hitting me with a ruler every time I went because she is very, very strict, very stringent. I mean, she is doing it for my health, but now she pats me on the back and says you are doing good. So.

I: That's great, that's good news. And so, in your eyes, an exercise program would be successful if it allowed you to continue to be stable. Your kidneys to be stable.

P: Absolutely because it is my understanding from a scientific standpoint that most kidney damage is never reparable. Some is but you know, what you have lost, you can never get back to full. So, you are only saving grace is to keep it stable where it is. And like set up and very successful in doing that and I do believe my exercises definitely been the major factor in that.

I: Okay. Alright. That's great information. So, we have two more questions. This next question is going to talk a little bit more about the logistics of the class. So, if we do decide to create a group class, we will need exercise instructors. And so, um if you were attending an exercise class, um, what could the exercise instructor do to help you to be more willing and excited about exercising. So, in your case, you are very willing and excited, so I guess try to put yourself in someone else’s shoes, and so what would that instructor have to do in order to motivate somebody that is not all there yet. Does that make sense?

P: I think first thing is, is one, to make it fun. Fun and enjoyable. Not like, oh my god, there is the drill sergeant, they going to put me through my places and I am going to hate every minute of it. Make it fun. Make it enjoyable. Involve everybody. You know, make it group oriented versus just treating everybody like an individual. Not to, that’s a fine line to walk because everybody has different abilities and interests. But, yeah. And give positive feedback versus negative. You know, make people feel involved and feel good about what they are doing. Let them know that they are doing things right. You know, I don't know how you plan to measure results, you know, like I don't know the logistics of how you do that, but just make it where it's, you know where you get up in the morning. Like when I run with my running group, man I look forward to it, I want to go. Because I enjoy their company. [40:00] And we, we all positively reinforce everybody efforts. Uh, and if one person raced the previous Saturday, and we are meeting the following week, we, we show interest in what they did and how they did. You know, positively give them, you know positive constructive criticism or give them a good pat on the back or whatever. If it is just uh, if you got and instructor that is like, they hate to be there as much as the participants and you can sense that, then everybody loses interest. I mean, it just got to be, you got to find someone that has great interpersonal skills. Um, you know, that enjoys and really wants to be there, helping people get better.

I: Yeah, I 100% agree with you. And so, let's see. In terms of like goal setting, so, how helpful do you think that would be. I know, it seems like you have a lot of goals. Like you continue to run a lot of races. But for somebody who isn't as active, like how helpful do you think goal setting would be for them?

P: Uh, I mean, goals can be any marker. Can be uh, somebody that just got border line blood pressure. You take there... I wonder how often is it going to be, is it going to be once a week, twice a week, whatever. You know, maybe you got a goal to lower their blood pressure. And maybe you give a chart of that. Maybe they want to lose a little weight. And, you keep, that can be a goal. A goal doesn't have to be, I'm going to run a twenty-minute 5K or I am going to run a mile in 6 minutes. It can be, you know different things. My wife, one time, her goal was to lose a little weight. She started doing that and then, she kind of got interested in her times and then, her overall fitness, and then running lead to going to the gym. And then it led to yoga and then it led to Zumba. I mean, there is other goals that people may want to get involved. I mean, you probably got grandparents can't keep up with their grandkids and their goal is to be able to go out and pitch a baseball with them. I mean that is a goal. I mean, you can set any kind of goal. Like say with kidney disease, I don't know really what, other than, you know your kidney function, to maintain that, I don't know what, I don’t know scientifically how you can do that. But, there are other goals in life. I mean like blood pressure is probably the biggest with uh kidney disease and uh weight.

I: Yeah, I think that uh...

P: Exercise so you can eat a donut. [laughter]. You know. That is what we always kid about. We like to exercise and run because we like to eat.

I: Oh, I do like to eat.

P: And if I didn't run, I would weigh 300 pounds.

I: So yeah, I definitely agree like goals can be something that is a number, but it can also be like a personal goal. So yeah, I definitely resonate with that.

P: Yeah, I mean you know. Some women may want to get into a size 10 jeans for herself and that would be her goal or... Or just, be able to swim. Maybe somebody was a good swimmer when they were young, and they gave it up. They'd like to swim again but, they are just no physically fit to do it. Like they get to swimming again. Um, we've got, I'm not involved in it, but we've got a club up here in Blueridge that uh, they walk a couple of days a week. They are trying to get over people to walk. You know, the goal is to get them out of the house. And get them off that recliner chair with the remote in their hands. So, like you said, goals can be [45:00] anything, it doesn't have to be a number per se. And I think, with the older people, and I don't know if there is a lot of younger people with kidney disease. I mean, I know there is like really young people with kidney disease. I mean, different goals for different people.

I: Got it. Alright, and thank you for that. And for our last question, we are going to switch it up a little bit. Um, and talk more about um, some logistics of the program. And so, we are also considering using a mobile phone app as part of our program. And it would help us track progress and that kind of thing. So, I was wondering, if you could tell me your current or past experiences with using a health app on your mobile phone.

P: Okay, well like I said, my wife is the one with a smart phone and um. So, my Fitbit is tied with her phone. And my Garmin is tied with my mac computer. So, when I do use them, which is not real often, you know, I have to either, bother her, and that is why when we were setting this thing up, I didn't want to uh, use her phone to talk and tie her phone up. It was okay to text on her phone, but it is better to use this phone for talking. But, um, you know she has apps, she's got her Fitbit too. And it is tied to that. [asking wife in background] And, what is it, do you have some other exercise apps? You know, what STRAVA is?

I: I think I have heard of it.

P: STRAVA? Or something, are you familiar with it. Are you familiar with STRAVA?

I: I've heard of it. Is that a mobile phone app?

P: Yes, it is. I've had that on my iPad. And, um, of course, I have to manually enter my stuff because I don't use, I could have my Garmin hooked up to it, but I don't. But like, a lot of the running community uses STRAVA, well even mountain bikers and road bikers use that too. You can try just about anything. Gym time, running, walking, biking, elliptical, that type thing. It will track all that. So, I would think probably because of my age, um, I am notes interested in technology like said, my whole career, I kind of, was forced to be a slave to technology. So, its kind of repulsed me. So that's why I don't do it. But of course, their younger generation, their tied to it, their whole world is tied to it. So, you know I think those apps are very beneficial and for someone who is maybe not as motivated or let's face it, who works full time. And can't just pop out of bed at 9AM and go to the gym. You know, that is a really good reminder or motivational tools to, you know the Fitbit which kind of buzzes you when you are sitting on your butt for too long. You know, you need to get up and walk around. I mean, I do think they are very beneficial. I am not against them, it is just personally, unfortunately, the two careers I had in my life, they were all 24/7 type, it wasn't just a 9 to 5 or 8 to 4. They were 24/7 jobs and, you know I just, was tied to some type of device, most of my working career, and I just, you know, I was the only guy in my neighborhood that had a computer stand and a printer stand in my car.

I: In your car? Can I ask what you did? Like, why would you need a printer in your car. That is my question.

P: To print out reports and stuff.

I: So, you were, you were surrounded.

P: Yeah, I mean, it was just you know, you couldn't go anywhere without, I mean even on vacation. We would go on vacation. I even actually quit a job one time because I was on vacation, at a family reunion, and I spent all my time, on the blackberry or the phone [50:00], talking about different issues at work. So, um, you know I did that all. Of course, part of my life, there was no technology. I mean, it was the cutting-edge technology at the time. So, I remember the gigantic phones and all that jazz. So, I have seen the whole evolution of it. You know, it doesn't interest me anymore. My wife, she is very interested. You know, I got an iPad and a Mac desktop and all that jazz. I'm not all like completely cut off from the world. You know, I don't want an apple watch or any of that kind of stuff. But, I think an app, yes, I am very in favor of that for the program even though personally, personally I don't need them because I am self-motivated. Where a lot of people may need them. And, you know, and also, it's good for motivation, that they can see the progress. Like you can graph out progress, you can see what you have done in a day or a week. Like, it gives you the weekly recaps and you can tie that into your friends and you can have little competition so to speak. Well Sally Sue did 80,000 steps, I need to do 82,000 or whatever. So, I mean, I do find it very beneficial and I think it would be a fabulous tool for somebody to have.

I: Yeah, so we are considering using one. But, um, sounds like, well, let's just say hypothetically that you were to be in the program and that you were to use an app. Would it be correct to assume that the app would need to be, an easy to use, and uh, not time-consuming app would be more appealing to you versus something um, besides that. Would that be accurate?

P: Yeah, exactly. Something that would kind of, keep track. Actually, probably one that would automatically generate results and sending it to me versus me having to go dig for it. And like say, that is one thing I do like about the Fitbit. It's... [asks wife, would you say Fitbit is easy to use] ... Yeah, I think Fitbit is a very um, would probably be, like I said, it sends you the little records and sends you the little awards that you reached during the day and their level. You've walked the great wall of china or whatever. That's, that's, for most people, that is pretty neat. You know, you got to remember, well maybe you don't remember, I've been up to national championship. I mean, getting the great wall of china medallion award doesn't really thrill me. Because, I have been on a national, because I have won national awards. But to the average joe blow, that would be a big incentive maybe.

I: Those in-app achievement awards. That is what you are referencing right?

P: Yes, exactly. Yeah, something that says you know, you have reached a goal. You know. And give you an Atta boy. Cause you know, the whole basis of being a successful athlete or successful coaches, every once in a while, somebody kind of patting you in the back, saying you did a great job. And, if that if running a 4-minute mile, or walking a mile, that can be an um, you know, a big barrier for somebody you know, that maybe they never been able to walk a mile or whatever. You know.

I: Yeah, I see what you are saying. But [name], that concludes our interview, thank you so much for all the information you gave me. I think it would be very helpful for our program. Before we conclude officially, I just wanted to ask you if you had anything else you wanted to share with us, or talk about, or clarify from what we have discussed today.

P: No.

[55:00]

Closing Remarks - End of Interview

**Audio File Name: E-02 10-26-2018.mp4**

**Recording Date: 10/65/18**

**Interviewer: AB**

**Transcriber: AB**

**Dates: 1/12, 1/13 2019**

**File Name: #2 CKD Interview Emory University (10-26-2018)**

[00:00:00]

I: Talking about your exercise, perceptions. So, the first question we have for you is can you tell me about a time you were physically active that you really enjoyed.

P: Uh that I was physically active?

I: That you really enjoyed yes.

P: Well I like to um, skate but it’s been a while.

I: Like ice skate?

P: roller skate

I: Okay. And is that something that you really enjoyed?

P: Yes. As far as physically fit.

I: What was it that you really enjoyed about it?

P: It’s just the entertainment you know the music, and the exercise. You know, going around and around.

I: Yeah. It sounds fun.

P: My main way to exercise. I like to lift weights too, but I can’t overdo it.

I: So, you do you lift weights occasionally but not a lot of weight. Is that correct?

P: Yeah occasionally.

I: Occasionally. Okay.

P: Not often cause like I said, I can’t overdo it.

I: All right. So, then our next question is, what do you think when you hear the word exercise?

P: Uh just being fit. Improving my health.

I: So, when you say when you say being fit can you expand on that a little bit more like what does being fit mean to you?

P: Just being in shape you know. To help, help maintain your, your health. Like, because it helps your heart and helps everything your blood circulation. Giving you energy and all that stuff. Just being in good shape, having energy.

I: Having energy, okay. All right so we're going to start talking more now about your current or past exercise routines. And so, the first question is, tell me about your current exercise routine.

P: Really, I haven’t doing too much exercise lately, so. J. Every once in a while, I get out and I walk my dog and lift weights every once in a blue moon but, not often.

I: Do you walk your dog every day?

P: Yes.

I: And so how often do you walk your dog. You're like know how often sorry how long?

P: Every day. About 30 minutes...

I: 30 minutes. OK. And do you know distance by chance, like a mile?

P: No, I don’t know the distance.

I: Is it just around the block?

P: Yes, pretty much.

I: And so, is this your usual amount of physical activity?

P: Yeah, I have been trying to work on being more. That's why I decided to join this program, time to get more motivated and work out more.

I: OK. How so, it sounds like lately you haven't been doing much exercise. And so, did your exercise routine change when you were diagnosed with kidney disease? Were you more active before or could you tell me more about that?

P: Yeah, I was active. I used to work out a lot. Do a lot of cardio.

I: That was before you had kidney disease? When you were diagnosed with it?

[00:05:00]

P: Yeah, I use to work out more before I got diagnosed with kidney disease and when I got diagnosed with kidney disease, its kind of slowed me down.

I: OK. All right. So, then the next question. will be talking about some of the benefits of exercising and then I know we talked about this a little bit in the introductory questions but we're going to ask you to delve deeper into it a little bit. And so, in your opinion what are some of the benefits of exercising and these could be health related and also personal benefits.

P: Yeah, I thought I just answered that question. That’s why.

I: Just being fit, improving your health.

P: Yeah, basically. That is what I do it for. Because I really don’t like exercising, but I do it for that because I know it benefits my health. It helps my health.

I: Is there another reason besides benefiting your health. Are there like more personal benefit?

P: No.

I: No. OK.

P: That’s the only reason why I do it. It benefits my health. It helps, helps with my health. And that's the reason why I do it.

I: OK. And so. What motivates you to be physically active? So, from what I'm hearing improving your health is a pretty big motivator. Are there any other motivators that encourage you to be physically active?

P: Uh, just looking good in my clothes [laughter]

I: Yeah. That's important. Anything else?

P: Uh, that's pretty much it. Staying young and you know, when you exercise, its kind of helps you to stay more young looking. At least I think.

I: Okay. And so, we can move on to the next question. So, the next question is talking about exercise specifically for patients with kidney disease. And so, the question, is how important is exercise for patients with kidney disease, in your opinion?

P: Well I mean I think it's I think it's very important. I mean it will help the kidney. It will help the kidney. Help the kidneys, the circulation you know, because I mean exercise helps circulation in your body so. I think it would help that tremendously.

I: Okay... And so, have you talked to your doctor or has your doctor talked to you about exercising kidney disease management?

P: Yeah. My doctor’s have mentioned to me various times about it’s good to exercise. To exercise.

I: Mm hmm. What, what exactly did they say. Did the same thing this specific or?

P: Well I was told that I need to exercise more because I, I know recently I was told that I have some cholesterol problems. So, that's one reason why I was told to exercise more.

I: So, did they give you like specific advice on how, like maybe the number of minutes you're supposed to exercise or like how hard you supposed to exercise?

P: Yeah, probably at least 30 minutes a day but if possible, an hour.

I: Okay, and this is your primary care physician?

P: Well I received the papers. You know, my kidney doctor had talked to me about my cholesterol. The last time I received papers instructing me about exercise and stuff like that.

[00:09:59]

I: OK. And these papers they were like health pamphlets or brochures?

P: Yes

.

I: OK. All right. And so, we can move on to the next question which is going to talk about some barriers to exercise. And so, the question is, what are some of the things that make it hard for you to exercise?

P: Well, what makes it hard for me to exercise is that I have a back problem. So, that kind of, I have herniated disks in my back and that is what makes it kind of difficult for me. Other than that. It’s not really... [inaudible]

I: And so, health related your back prevents you from exercising as often as you would like. Would you say there are any other barriers? I know you said you drove Uber and Lyft part time. Is time an issue for you?

P: Uh, well, if I am driving too long, my knees will kind of swell up. You know, I have to get up, but I just found out that stretching and exercise so, that kind of helps with that.

I: Okay so the stretching, stretching and exercise. Okay. How, so like do you drive Uber every day?

P: No. No I don't go in every day.

I: What times of day do you normally do it?

P: Normally during the morning.

I: Morning like early morning?

P: Huh?

I: What would you say early morning?

P: Yes, early morning from 6:00 in the morning until like lunch and then I'll take a break and then um, then I'll go back out about 6:00 pm. Sometimes. It all depends how I feel. I said I don't drive every day.

I: OK. And so, if you were to exercise would, you would, when would you most likely do that? Like you said noon right around noon. Is that correct?

P: Yeah. I would do it around my lunch break.

I: Around the lunch break. Got it. So, what would make it easier for you to overcome these barriers. I guess the herniated disk is something that's not easily solved. But… are there any things that would make it easier for you to overcome the barriers?

P: Yeah, well if I didn’t have herniated disc problems, it would be much easier.

I: Is there anything else?

P: Well, no it’s not really nothing. Well I mean I do get, I do get tired faster and I do get tired faster with the fact of me having kidney disease and lupus.

I: Oh, you have lupus... okay.

P: Yeah. Lupus is associated with my kidney disease. That's why I have it. Lupus got into the kidneys. And it is called lupus nephritis.

I: So those two things make you tired faster.

P: Yeah. And that’s why I can’t overdo anything.

I: Okay.

P: Did you hear me?

I: I did. I did.

P: Okay. My phone was connecting to the vehicle from the Bluetooth. Sorry about that.

I: No worries. Are you driving now?

P: Yes.

I: OK. Is that still something you're comfortable with. Talking while.

P: Yeah, I am fine. I am on the speaker phone. I am talking through my speaker, the vehicle speaker.

I: OK. Speaker just double checking. OK. So, talking about back problems, you're talking about getting tired faster because of the issues you are dealing with.

[00:15:16]

I: So, let's see we can move on to the next question. And so, this question is going to start talking more about the actual possible program we're going to implement. And so, we are considering a group exercise program for people with CKD. How do you feel about group exercise classes in general? Is there things that you like? Are there things that you dislike?

P: Um, I don’t mind doing a group, you know, group activity. I am a people person so. I have no problem with that.

I: OK. And so, you're a people person you liked to interact. How do you feel about an exercise class created especially for people with kidney disease?

P: I think it would be pretty cool and work out with, you know when, you know you have something in common with, you know and getting to get to know other people and what they experience and go through on a day to day basis.

I: Like having a space to share your experience is kind of.

P: Yes

I: Okay, and so would there be any barriers for you in participating in a group exercise class?

P: No, no and how does the program last?

I: We are still in the development stage, but I think it would be around an hour. But that's subject to change. It wouldn't be very long, but we would definitely not make it a very short program.

P: Oh okay.

I: So yeah once we start collecting all the information from these interviews then we can start to tailor it specifically to people's needs.

P: Okay.

I: And so yeah. So, how should we design these classes? Is there like any war like how should we design them to be most convenient and appealing to you? And so, for instance like what kind of activities would you like to participate in in these group classes?

P: I mean, I really don't know how to answer that question. I mean it doesn't, it doesn't really matter to me what type of. Activities that participate in. I pretty much like all type of exercise as long as it don't put me in any pain. It doesn’t matter to me.

I: Okay as long as it me as long as they don't put you in pain. Okay.

P: I am pretty much open to any activities.

I: So that includes like maybe like running, lifting weights?

P: Yeah. Yeah that's fine. I can try to do the best I can [laughter].

I: And so, I know in the beginning you were also talking about how you like to roller skate and you liked the music and so it sounds like you would also like something that's fun. Am I wrong or?

P: Yeah, like aerobics. Like aerobics class.

I: Like Zumba maybe?

P: Yeah, I was just about to say that. Like Zumba.

I: Zumba, aerobics, something with a little bit of rhythm.

P: Yes.

I: Okay so in terms of location do you have a specific location that you think that would be most convenient for you?

[00:20:02]

P: Well I don't know where the locations are yet.

I: So, since this is a study what people would CKD, it could be like a local hospital.

P: Would that be OK with you?

I: Just do you have a specific preference or do you just like, wherever it is like, if it works in my schedule like I can make it.

P: If it’s not too far, I mean… I don’t mind as long as it is not too far.

I: OK. And what's too far for you. Just like if you were to define that like an hour away. Yeah without traffic or with traffic?

P: Well I mean I understand if I'm in traffic for an hour I'm used to that. Here in Atlanta, in Atlanta, so I can understand that sometimes you get stuck in traffic and it takes about an hour to get somewhere. So, I would say without traffic.

I: Perfect. Thank you. And so now we have three more questions. Thank you so much again for everything that you've said so far. The next question is what would you value most from an exercise program?

P: Getting in shape

I: Getting in shape, okay.

P: Bettering my health. Like I said I’ve been having cholesterol problems lately. I need to lose some weight gain because I’ve gained weight this past year that I'm not used to having.

I: And so, you have a specific goal or number in mind?

P: Yes, I have a goal to be in shape by next summer which I don't think it's going to take that long.

I: OK. What about weight wise? Do you have a specific amount of weight that you would like to lose?

P: Well, my weight is fine but um, it's more fat and muscle.

I: Okay.

P: That's the thing. So, I don't have a problem with my weight. I just want to get rid of the fat and turn it into muscle.

I: Got it. OK. And so maybe some of that more like lifting aspects that you were talking about earlier would be a way to do that.

P: Yeah.

I: Okay, and so I guess this is touching upon a little bit of the same thing but how would you judge if the exercise program was successful. If you achieve these goals, is that is that one way you would measure success?

P: Yes.

I: Okay. And so, so like measuring individual success and your own personal success, we are also wondering like how would you judge if the entire program was successful not just for you but for everyone.

P: You got kind of blurry there. I didn't quite hear the question clearly.

I: Oh okay, sorry. So, we're talking a lot about like your own personal individual success, but we also want to know like how would you judge if the exercise program in its entirety, so everyone involved in it was successful. Do you have any opinions on that?

P: You said how would I judge the program entirety?

I: Yes... So.

P: Well, I really wouldn’t know that until once I get involved in the program and the program is done in order for me to judge.

I: Okay, that’s fair. And so, our next question is going to talk more about your specific experience with exercise instructors. We are thinking of having an exercise structured instructor lead these group classes. And so, if you were attending an exercise class what could the exercise instructor do to help you be more willing and excited about exercising?

[00:25:10]

P: Well uh, you know, I guess just walking and motivating. Motivating the class, pushing the class, telling them they can do it. You know, just encouraging them. I’ve done aerobics class in the past, Zumba and stuff. They make it more exciting, you know. Instructing the class, telling you, you know, what type of exercise to do. And doing it with you.

I: So, you want him to do it with you. You want it to be motivating, encouraging. And so, is there anything else that they could do?

P: Well not be too rough [laughter] and taking consideration, you know of people with health conditions.

I: OK. So, let's see. So, you talked about this a little bit already but in terms of goals would you want your exercise instructor to help you with setting goals?

P: Yeah. That would be fun. That would be good.

I: Okay. Alright so here we are on our last question so thank you so much [name]. This question is going to talk more about how we're going to apply, some of the ways we're going to apply the program and so we are also considering using a mobile phone app as part of our program. And so, can you tell me about your past or current experiences with using a help app on your mobile phone?

P: Well I tried to use the app to do some yoga one time and it was quite difficult trying to look at the phone and do the pose.

I: Oh, I see. It was a it was an app focused on just doing yoga. It wasn't. It wasn't like one of those apps. Did it take your steps the many steps you take or do any of that kind of thing?

P: Well I have stuff like that on my cell phone already.

I: You do okay.

P: Through Samsung. It tracks my step and it takes my pulse and I can put in how much water I drink a day and caffeine and it tracks my sleep. Really how it does that is, by me touching the phone, the last time I touch the phone at night before I got to sleep and then when I wake up and touch it. That’s how it tracks my sleep which that’s not really accurate because I may still be awake and just put my phone down at night time. You know?

I: Yeah, I see what you are saying. And so, I know you're thinking of using different kinds of apps that would probably… probably measure your sleep better than that. And so, I guess for your Samsung like how it tracks your steps like are there... do you like... what's your… oh do you like it overall?

P: Oh yeah. I like it. I don't hardly use it, really, I mean, uh. I mean the times I did use it, it was alright

I: How often would you say you do use it or check it at least?

P: Well when I first got the phone I would only use it for like a couple of weeks and then I left it alone. I can see, I can see where it tracks my sleep every day. I mean it pops up on my phone so and then if I walk a certain amount of steps, it would be like, you reached your goal because I had it set on a certain goal which I don’t remember what that was [laughter] because it has been a while.

I: Okay. Yeah, they do, they do set goals they have achievements now. Okay let's see.

[00:30:43]

I: So that concludes all the questions I wanted to ask you. Thank you so much again. You have given us a lot of very helpful information and so we really appreciate that. Before I go. Is there anything else that you want to share with us? Talk more about or clarify from what we discussed today.

P: No.

I: Well like I said thank you so much and I appreciate all the helpful information [name] Have a great day.

**Audio File Name: E-03 11-2-2018.mp4**

**Recording Date: 11/2/18**

**Interviewer: AB**

**Transcriber: AB**

**Dates: 1/14/2019**

**File Name: #3 CKD Interview Emory University (11-2-2018)**

**E-03 (11-2-2018) Full Interview.m4a**

[00:00:03]

I: Exercise is Medicine CKD study I.D. number three. All right. So now I'm just going to read you the introduction, just the generic introduction and then from there we'll ask you a couple introductory questions. So, hello my name is Alan and Benita and I am working with other researchers here at Emory University in Atlanta to understand the experiences people with kidney disease have with exercise. What you tell us today will help us to create a program to make it easier and more fun for you and other people with kidney disease to be more active. Before we start a discussion. I want to go over a couple of things with you. I want to hear about your own personal experiences. There are no right or wrong answers so please be honest. Everything you say today will be kept completely confidential. And to help us catch everything where we have this audio recording device. If you have any questions, please feel free to ask me more. If you need any clarification on a question, please do ask and we anticipate that this interview will last anywhere from 45 to 60 minutes.

I: All right. And so, before we start we want to learn a little bit more about you. And so here are a couple questions. So, first question is can you tell me about a time you were physically active that you really enjoyed other in high school?

P: Probably when I was in high school.

I: Okay. What do you do in high school.

P: Oh, that was probably when I was messing around with baseball and football. I never got to really play because my parents wouldn’t let me play but I scrimmaged then I worked out with the team, but they wouldn't let me play in a real game.

I: Oh really. Yeah.

P: Because they told me you would get hurt. I was also the biggest guy in the whole place.

I: Yeah. They must have wanted you on the team.

P: They wanted me on the team real bad. So, they let me practice for them for two years before I you know, I just step away because of insurance reasons.

I: So, it was baseball and football?

P: Yeah baseball and football.

I: And so, since high school like when you do exercise, like...?

P: I don't really exercise much. I mean I used to do more until recently because they put me in the office. But I used to work doing between five to seven miles a day. I’ve walked, and I've always been heavy though. I don't walk as much anymore. I don't. I'll be surprised if I walk two miles a day right now.

I: And so, going back to that baseball and football or it just in general exercise, what is it that you enjoy about it? Is there any particular thing that?

P: No, no it’s just fun stuff...

I: So how recent was it that you were moved into the office?

P: Oh about 2015. I mean, I’ve always had jobs doing both, you know. So, to me, it’s not a big deal...

I; All right so next question. What do you think when you hear the word exercise and how do you feel?

P: Nowadays, I feel like I have to go to the gym and hang out

I: So, you have to go to the gym.

P: Well I mean everything else just a pain in the butt... I mean I'm not a streetwalker. Well that don't sound right either. But I don't go. I don't like to go out and walk down neighborhoods. I just don't get into that. I'm not doing that because I do that all day long for a living, so I don't want to see it just get [inaudible]

I: And so, again so you would say that... would you say So, you're not a streetwalker.

P: Well I do walk the streets a lot because of the nature of my job [laughter] But I don't like to get out in the neighborhood and walk around rolling, go to the mall and walk. I just don't get. I've never got into that.

I: OK. And so, when you say like gym, like going to the gym

P: I just don't have time. I would like to do that. But I don't have time. I'll leave the house at five thirty in the morning. And on a normal work day, I get home at 8 o'clock at night while I'm gone all day.

[00:04:50]

I: You’re a busy guy. So, the next couple of questions I’m going to ask you are going to go a little bit more deeper into the current exercise routines or like your personal experiences with exercise and also your like attitudes towards it. So, the first question is tell me about your current exercise routine. And so, I know that you walk a lot.

P: But I don't walk like I used to. I only walk maybe a mile to two miles a day. Maybe. And then just some days vary.

I: And I'm guessing that's two miles over the whole day?

P: Over the whole day, yes. Yeah. I mean some days. All I do is just walk around the office in circles. I feel like a hamster.

I: Do you take breaks and walk or?

P: I have to get up from my desk to if I sit there too long. My legs will get poofy, so I got to walk a mile.

I: Okay And so for the past three years you would say that it's been this way?

P: Yes. Oh about the last 18 months I've been more struck in the office in then the first 18.

[Phone rings]

I: You want to take it or?

P: I’ll call them back.

[00:06:36- Continue talking about phone call]

[00:06:58]

I: Okay, so kind touching upon the same thing, but besides walking is there any other type of a physical movement that you do outside of work when you're at home?

P: I mean just take the dog out for a walk. But my wife wants to get rid of the dog, so we give it off to my son on Sunday. So, I won’t have a dog anymore.

I: How long have you had the dog?

Uh, a year. We got him from some nurse that we know, got him from a nurse friend of hers P: ended up having to go to China. So, but she couldn't handle the dog and we've had Siberians for a long time, the Siberians, to us their easy to deal with but my wife doesn’t want a knock-on other programs injury to us are easier to deal with. Yeah but my wife doesn't want to go anymore. So, she got the dog to make me happy for a little bit and now...

I: She’s taken away.

P: Yeah, I’m going to go [inaudible] in Athens.

I: Ok, at least you’ll know where the dog is.

[Continue talking about the dog]

[00:08:08]

I: Would you say, how long do the walks normally last?

P: With him? About 10 to 20 minutes.

I: So, since you were diagnosed with kidney disease has that had an effect on your exercise routine or like the length you are.

P: Well when I got diagnosed, that's about the time I started getting transferred to a more indoor work in my office and I mean I try to redo things with some guide. I backed off some salt. And you know, just different things to try to keep it better. And you know, I gave up all carbonated drinks. I’m now, I’ll have like even teas, I’ll have honest tea or team made at home because it doesn’t have some of the stuff that is bad for your kidneys. I'm mostly a water drinker anyway. I mean everybody likes the occasional coke you know. I don’t have that anymore.

I: Water is good for you.

P: Yeah.

I: Okay, so moving on to the next question. Uh, what are some of the benefits of exercising in your and your mind?

P: Well as far as I know, it helps with your muscle strength. It helps increase your bone density. It does help your cardiovascular health and it does help control cholesterol.

I: So, a lot of benefits.

P: Yeah. And theoretically you don't get as tired as these during the day. That’s theoretical

[00:09:59]

I: And so, are there other benefits besides health benefits? So, I guess like more like similar to not getting tired all the time.

P: Well you know I’m... I've always been a high energy person and I got older it got down. 18 years ago, I used to work toward one in the morning and I was at the office at 6a.m. again. So, I was like up and down. And you know... I don't think I could do that anymore even though I go home. I mean I'm up at five thirty. I’m out the door most days I don’t get home until 8 o'clock at night which I don't like really doing that.

I: That’s a long work day.

P: Yeah.

I: Um, do you have. I guess we'll talk about this later too. So, do you... are there anymore motivations for you to be physically active like outside of work.

P: I’m always at workI mean I would like to. I talk to my wife about trying to figure out a way to get home earlier. I mean we hit the gym for a little bit. Three times a week, four times a week and then she looked at me and says when are you going to do that? Mm hmm. She's right. I mean. If I leave the office at 3:00, the way traffic is, I don't get home till 6:00. And it's crazy because it's not that far. On a Sunday I'm home in 45 minutes if I come in on Sunday. But it's just the same.

I: Okay.

P: I'm looking for ways to switch but it's I don’t know.

I: It has to fit within your schedule.

P: It has to fit, and the income has to fit too. [Brogdale], there is not whole lot of stuff over there. So, I mean we moved there when the kids were little because the schools were good. Now the schools are bad, but the kids are grown up. Now we're trapped. Can't move [chuckle]

I: No. Well hopefully this program will create a create something that will be applicable to your situation.

P: Well, that’s why I went ahead. But you know it's worth looking into because if you keep the door closed you'll never know what's behind the door. You may not want to know what's behind the door but

I: Alright, next question. How important is exercise for patients with kidney disease? Well and I guess a better question is, has your doctor talked to you about exercise and kidney disease management?

P: My internist has never believed me how much I work or walk now. He just doesn't. My nephrologist does not think I am obese.

I: Does not think you're obese. Okay.

P: He says, I need to kind of watch my sugars the way they're going now. And at all possible you know, avoid blood sugar medications unless my sugar starts getting you know, too high. Actually, my internist is the one that's helped me manage my kidney stuff better and also my blood pressure. So, my internist, I just told her because I am supposed to see an internist.

I: And so, do either you, your internist or your nephrologist, do they talk about exercise at all?

P: Yeah well, she does not think I actually, even when I was walking 5 to 7 miles a day, she did not believe me.

I: Really?

P: And I am I can't. My blood pressure was through the roof, so I don't. But if she would not be able to keep up with me. Because I used that police officers follow me to watch cars for me because I would be the middle of traffic during the day and they looked at me and, especially after I had heart surgery and they were breaking a sweat and they said, can slow down for a minute? And they’d say how do you do this? And I said, I just do it. An officer cannot keep up with me and that's how aggressive used to be. And you know, right now one of my reasons I have low energy also is because of my red blood count, lower hemoglobin, [mumbling inaudible] I mean it's gone better. Three years ago, it was 8.5. Now it's like 10.7

I: Yeah, that’s a big improvement. OK so your nephrologists sees you being sees as not obese, but your internist doesn't think you exercise as much as you do.

P: Right. Now I don't exercise as much as I used to.

I: So, in terms of exactly what they tell us so like your internist doesn't believe you and so what would they say they're just prescribe you to exercise more?

P: She thinks I need to exercise more, and I told her, I said you know, well you know there's only so much I can do.

I: Did they give you like a specific number or?

P: Well she thinks I should use actually my treadmill broke down a year ago, so I can’t use my treadmill anymore. Well, I know how to fix it, but I haven’t gotten into it. But uh, she thinks that I just need to find time to walk more.

[00:15:23]

I: She doesn't give you like specifics?

P: No. She’s a doctor, she doesn't know how to say this stuff.

I: No like 30 minutes of walking a day? No?

P: Well she thinks you know, 15 to 30 minutes but I do that. Right now, I'm. She thinks I need to walk more, and I need to walk hard, as hard as I can for about 30 minutes. But I, I just, to find that 30 minutes to do that, is tough. Because usually when I am already walking, it is already for work

but my work is not, you know, you don't you're not racing. You're having to gather information from the roadways and so on. And you know, but I mean you, you need to be quick otherwise you don't make any money. You know, it's just the nature of the beast.

I: All right. That was great. Next question. Um we touched upon this already a little bit but we're going to dive deeper um what are some of the things that make it hard for you to exercise. So, time

P: Work environment.

I: Okay. Work environment and time. So those are the two biggest ones. Is there anything else?

P: I would love to make more time to figure out how to get some more exercise in because I know it will only help me. My kidney function down from 18 to 34 anyhow. And they did not expect it to get better. They thought it was going to get worse. My nephrologist two and a half years ago figured that right now, I would be on dialysis.

I: And you’re not.

P: I went the other way.

I: That's great news. You’ re doing something right.

P: I don't know what it is yet. Maybe I’m not drinking any bad drinks.

I: So, work environment, time at work, is there anything that would make it easier for you to overcome that work environment.

P: A different job.

I: Find a different job... but.

P: I mean, they have a gym there, about the size of this room. But they are all like kid stuff. There is nothing that is really going to put any You know. But I mean they have a gym there and he uses it. It's the size of this room. Mm hmm but they're all white kids’ stuff. It's nothing to really put anyYou know what, after I had my heart surgery and I understand this is different because don't want your chest opening up again and they had me working with 5 to 10-pound weights. I’m 6’’ 4. [Emphasis]. I used to be able to lift 300 pounds. And they put me in a room doing five to ten-pound weights. I hit myself in the nose one time [mumbling + laughter] I wanted heavier weights, but they said I couldn’t use them. And I mean, I did 8 minutes of rehab there, but it was just funny. I just, it was just, this was not helping me a bit.

I: I can, it must have looked very small in your hand.

P: Oh yeah. Now I know women that exercise with those and that is a workout for them, but not for me. But I did the bike thing and the other stuff that they put me through.

I: You were also talking about your legs swell up below?

P: Yeah. That's because of two reasons. One of them is the kidney issues and the doctor thinks it's the [medication] pills causing my swelling he does not want to take me off of it.

I: So, your medication.

P: Yeah. I take 2.5 of [medication], twice a day. He kind of wants to remove it but he he's still, the nephrologist is still trying to play around with that idea.

I: Okay. And so that's when you sit down. Right?

P: Yeah, If I'm up, I'm up and walking now. That's why I get up and walk around the office just to keep things moving.

I: All right. Besides that, are there any other health barriers?

P: Well, when I had my heart surgery, my right foot is the one section swelling up, where they my foot got deformed and they couldn’t figure out how to fix it until about two years and it started shrinking again. And part of it is just I think it's because my kidneys started getting better... But it is just that one foot for a long time. Both feet get swollen. That's why I kind of get a little annoyed with my internist because I went in there when I thought something was wrong. I was all swollen up. And as far as she was concerned, I was just over eating my weight because I went back up to 276. And I don’t... I don't think it's food. I don't think it's fat. It was really soft. And she said see you in four months than she walked out the door. She did lab work and sent me out. Four hours later I was in the E.R. My potassium had gotten [inaudible] and uh, they took me off all the potassium medication. Almost everything had potassium on it. And I fussed her about it a couple years earlier. You’re giving me too much potassium. Oh no, no. That’s hardly anything. When they took me off of it, everything started getting better.

I: Is she still your doctor?

[00:21:00]

P: She’s still my doctor. You sound like my wife. Is she still your doctor? [Imitates wife] She’s an Emory doctor. All my doctors, my cardiologist, are Emory doctors.

I: Oh. Well, it sound like you got a good nephrologist though.

P: Yes, I do. [Doctor] very good.

I: That's awesome.

P: [Doctor] connected me to him and I see [Doctor] and [Doctor]

I: Okay, let's see so it sounds like you're pretty motivated too. If you were able to overcome those barriers, that you'd be motivated to make the time to exercise. Right?

P: Yeah. I might have an option on the job in January.

I: So what kind of option?

P: A different company or different job. Maybe I won’t working seven days a week. The drive is going to be killer because it’s all the way over in Norcross. I do have a way to get to Norcross across quicker. Even though it's like the route is almost the same. All I do is I bounce to the east a little bit about. Come out that way. And I can avoid a lot of traffic.

I: Nice, so you know the tricks.

P: I know this city like the back of my hand.

[00:22:25] – [Parking conversation]

[00:22:56] Conversation resumed.

I: All right. So, the next section we're going to be focusing on is going to talk more about possible program components. So right now, we're in the process of creating an exercise program. And one of the things we're considering is having our program be group based. And so, how do you feel about group exercise classes in general?

P: I'm not going to conform to the group. In other words of the group says we need to meet at 2 o'clock in the afternoon. Sorry sayonara, I can’t do it because I have to work?

I: How are your lunches?

P: By the time I get here I'll have to go back. Yeah. Because if there's no traffic, it takes me 20 minutes to get here. I can go without lunch really. But it is not good for me. Because then, I’ll end up wolfing something later that I shouldn’t.

I: Okay. So, you would want flexible group dynamics or if it's possible?

P: I mean, if it’s at all possible. Honestly if it's after four or it’s weekend stuff. I can make it work. But if it’s during the week and have to be in the middle of the day, I can’t do it.

I: Okay, besides the time aspect of it. Let's say we do find a good time for the class. So, in terms of like exercises, do you like how do you feel about exercising with a group of people?

P: Okay. I don’t have an issue with that.

I: Okay.

P: I can't swim. But if you want me to swim, I cannot swim.

I: Okay I'll put that down.

[Phone starts to ring]

[00:24:59]

I: Um what would you like about a group class?

P: I’m indifferent. Is that a term I can use? I’m not anti-social. I get along with a lot of people.

I: But there is nothing that stands out to you? Okay. Um how do you feel about an exercise class created especially for people with kidney disease.

Same thing.

P: Mm hmm.

I: So, we actually talked about this already but various participating would be work and time.

P: Yeah, mostly work. If I can’t get you know like I said, if it’s in the middle of the day, I can’t do it.

I: All right. I like this question better. How should we design these classes? To be most convenient and appealing to you.

P: Any time if you can make it so that it is feasible for participants to participate. That sounds really high tech doesn’t it. You can be more successful, but you have to if you deal with a lot... of it you're dealing with people at work that are retired they are going to want to come in the middle of the day

[participant coughing a lot, goes and gets water]

[00:27:24] – participant returns

P: I think what I am trying to say is if you make it convenient people will come but it does depend on the type of people that you're going to have. Mostly that will come. You know retired folks don’t like to get out in the evening. There are people that can’t work because of their issues. They usually don’t want to get out in the evening either. I'm just... the doctor, my nephrologist says you shouldn’t be here saying that I shouldn’t have the issues I am having, but I am having them.

I: And so, let's say again that we were able to create a program that works time wise. So, like people will be there and we’ll be ready to exercise. And one of those people is you. What kind of exercises would you wanted to do. Like what would you find most appealing?

P: I prefer to lift weights.

I: Lift weights. Okay.

P: I'm not a yoga. No no no no no. I mean, I understand some of it but I mean I'll try it.

I: So, lifting weights would be your primary.

P: I think that's more beneficial to someone like me.

I: What about cardio? Cardio kind of things

P: That’s never been my strong suit.

I: Like a bike.

P: Oh yeah, a bike and stuff. That’s alright.

I: And then you said he you had a treadmill?

P: I still have it. I have to figure out what is wrong with it.

I: So, you use to run on it though?

P: Now that’s the one thing I cannot do on a treadmill. I can walk fast. I cannot run on it. I freak out. My cardiologist says come on when I do the stress test. I just have a hard time running on it. Don’t ask me why. None of my kids do. They all run on them.

I: But you would run on something that wasn't moving very fast like the pavement?

P: Yeah. For some reason on a treadmill, I'm not... it just doesn't seem natural to me to run.

I: Okay anything else? Any kind of activities? We've been thinking of like dance like trying to be creative about it. Make it fun.

P: Dancing to me is like

I: No, not a dancer?

P: My daughter says I can’t dance. [laughter]

[00:30:00]

I: Have you ever heard of Zumba. You just follow the... follow the instructor and they just tell you what to do.

P: I have no idea what Zumba is. I don’t have much of a social life.

I: Sounds like you work too much.

P: I also don't follow typical old folk routines. I don’t know what you’re getting into but uh [mumbling]

I: We talked about this a little bit earlier and so location wise, around Emory like the school of public health would be

P: This is probably one of the better locations. It would be better than the one on Piedmont. For me.

I: Just like transportation wise?

P: Well because I can cut this way on my way home. Okay so this Emory

I: What would you value most from, like an exercise program?

P: I think maybe it might even help me with my kidney disease. I mean that's why I'm doing it. Yeah. You know. Like I told you, I feel like in my office I am on a hamster wheel and when I get outThis is the quietest this phone's been all day. It goes off all day long. Constantly. So, it’s just like. Even when I’m out on the field I get stopped. And usually it’s questions I have to answer/

I: All right. So as long as it helps with your kidney disease, that would be valuable. Um and this question kind of moves into the next one. How would you judge if the exercise program was successful?

P: If my kidneys improved. [laughter] You know they went down to 40 and it stayed at 40 for three years. And then all of a sudden it hit the 18 mark which was... 18 was scary because at 15, you are at dialysis range.

I: What’s your range right now?

P: Right now, I'm at 33.5.

I: 33. Okay

P: I'll take that half.

I: Take as many halves. Yeah. And so as long if it helps you improve your kidneys. That would be successful. Anything any other indicators?

P: Well I mean you know my blood pressure. So, I tend to develop um I like cholesterol for some reason.

I: You like cholesterol?

P: My body does. I don't like to build it, but it keeps building it, I don’t know why. It's been low for the last several years which is good but it’s strange.

I: How would you judge if the program was successful for everyone? If everyone got healthy or...

P: Well, if people got healthier. They learn how to improve their life later. It's like anything. You know sometimes it's just too late. You know I'm saying.

I: Okay so, next question is what is your experience with exercise instructors and so on?

Have you had any? Like a coach?

P: Well, I have to careful the way I say this. Well, when I was in elementary school, all the bullies were friends with the coach. When I was in high school, I was trying out for football. One of the coaches thought that I did not hit my teammates hard enough. And my attitude was, I don't want to hurt him because he is on my team. You know when we go to play, they would never let me play because my parents wouldn’t pay insurance. And he didn’t understand my logic. Why do I want to hit this guy where I could hurt him? Cause they use to tell you. Hurt him. I mean I don't know how to do that now but when I my school they did. I had with fun with that. The coach would teach me how to hit him. I was 6” 2 sophomores in high school. And coach came at me in full pads. I picked him up and dropped him every time. I said is that hard enough? Because I'm not sure I do this correctly. He came at me again. Nailed him. That was a lot of fun.

[00:35:21]

I: You enjoyed that part?

P: enjoyed that part. But anyway, I just I just had my mixed feelings with coaches. That’s not the same thing with fitness instructors but

I: So, let's just think of more like the fitness instructor.

P: Yeah, I went back too far. [laughter]

I: But like it's like some someone that encourages you rather than like tears you down.

P: I mean, I had a fitness instructor about 10 years ago and was a very nice person, but I never worked with her. We just talked about fitness.

I: Okay, um so if you were to like just to put yourself in a situation where you were in the exercise class what could that exercise instructor do in order for you to be more willing and more excited about exercising?

P: Don't ask me to do hokey stuff? I’m not going to dance. I have no rhythm.

I: Okay so you want them they want you wanting to be flexible in what exercises?

P: Right. I'm more of a traditional exercise person. Does that make sense.

I: Yes, it does. Lifting weights

P: Yeah, I’ll get on the treadmill. I’ll do some jumping jacks and that kind of stuff... running in place. But not dancing and stuff. That’s just

I: Yeah, it's just it was just an option.

P: I know people think that's great. I know people that going dancing for exercise, but I just whatever.

I: Um let's see how helpful is goal setting for you in terms of exercising?

P: Well it depends what the goal is.

I: Let's say you're lifting weights.

P: Yeah. You know let's say you're lifting weights and you're like 150 pounds you say well you know and in six weeks I want to be a 200 comfortably. You know we hit 200 or 190. You're like OK that's better. And if you don't see an improvement you’re like, what am I doing wrong. I mean we all have limits. We're not going to get to a certain point where your body is going to say, this is all I can do. Yeah, I understand that part of it too. But it makes you better. You have something that you can see, something tangible and they have my opinion it has to be realistic goal setting. You know we can't say well, I'm going to do this in four weeks when it's going to be a long stretch maybe. You know hit it. You might be doing it. but if you're the type of person who gets bummed out when you don’t hit a goal. You have to be realistic with your goal setting.

I: Okay. How could the exercise instructor discourage you from exercising? Like what about them, other than what happened with your high school coach.

P: So that was fun though. You know but that was a different time. You could do that, even if you are a student. Now you can’’ take out your instructor [laughter]. Um... I don’t know. I mean just, I just don’t want to do dance and stuff. And now what is that. Those high intensity aerobics exercises. That sounds like dancing to me. I mean. You know when I was a getting out of college that was starting to get popular. We did some of that and some of the I got to take electives and stuff. Can I just go and play tennis and stuff?

I: So, I guess put aside the actual exercise and just think about like the type of person that you would want. Or like the type of person that you're you would want your exercise instructor to be in order to like motivate you. Would you say that you would want someone that motivates you? Like in a way that

P: You know, if I see the value in something, I'll motivate myself. That's the best answer I can give you.

[00:40:00]

P: I'm not judgmental on people even though everybody is. But you know I'm not going to say well that was not a very good instructor. [mumbling] I'll do the best I can no matter who is there. I’m just here telling you what I don’t like to do. My daughter says you can't dance dad more than once. So, when your daughter says you don’t.

I: Or you could prove her wrong.

P: No, she's unfortunately as bad as I am on some things.

I: Well we are on the last question and so this one this question talks about the mobile component of our program. So, we're considering using a mobile health app like a Garmin or Fitbit app as part of a program to track like your progress and like how you're doing, and so can you tell me about your past expand past or current experiences with using a health app on your phone?

P: I've never had but I've always thought about putting... I've been wanting to at least, I’ve been thinking about the idea of getting one to keep track of my blood sugar all the time. B

I: Blood sugar OK.

P: I think that's one of the biggest things I need to really focus so I don’t get back on... I’ve being able to control the diet, but I am like just kind of in the grey zone and I am trying to keep it more in the white zone. So, I’ve been thinking about that, but I don’t think this phone can handle that app.

I: You'd be surprised.

P: I’m waiting for 5G to kick in because I’m the nicest phones are at 1000 dollars. The 5G phones, I have no idea what that would cost. And I am contributing to 5G. I’ve been helping find fiber routes for 5G.

I: Cool, that’s awesome. When’s it coming out.

P: You are probably going to see stuff sometime spring, early summer. Because I think by next Christmas, they want to hit you hard on cell phones. Because unfortunately most of these are not forward compatible.

I: And so, the blood sugar app. Have you ever used an app that tracks like the amount of steps that you do?

P: No.

I: No, okay.

P: Would you be open to doing that?

I: Oh yeah

P: [Sigma] would love it.

I: Who would love it?

P: [Sigma]. My insurance company. Oh. They do stuff promotions like that always.

I: I think my dad did something like that too. Let's see. All right. Is there anything that you don't like about apps? As long as it's a functional. [inaudible]

P: I taught my kids in the 90’s how to take apart computers and put them back together and apps and stuff. I mean, I am not scared of technology. I’m just cheap. I don’t see buying a thousand-dollar phone even though I like them. No, I am not going to do it.

I: All righty well that concludes our interview. Do you have any questions, or you just might need some clarification from anything we discussed?

P: No.

I: Mm hmm. Okay well thank you so much. I appreciate you taking your time out of your busy day to come here as well as things which may be beneficial to me for them really open the door.

Yeah. Hopefully the information that we gain from you but also the other interviews we do will help us create or create a program that works for people but also that is engaging and appealing and I think actually makes a difference.

P: Yeah, I'm not really sure what your target people are which is okay that's probably for your own private study.

I: Well it's, it's just people like the target is people and CKD. And so, a program for them to exercise because studies have shown that any form of physical activity for people with CKD improves overall health outlook. And so, we're trying to do that here at Emory.

[00:45:00]

P: Well you know it's very discouraging to me is when I go see my nephrologist in Covington and I see his other patients come in they're just dragging they're just like they're barely alive and I keep looking at them. I don't want to be there. I don’t know if that probably helped me figure out how to not get worse. And I don’t know if haven’t gotten worse by sheer luck or having a good team helping me. And the same thing when I go to the other clinic in Piedmont. You know I see some folks and they're going to the kidney side. And I look at them and I don't see anyone that's like me. You know, I see them, like you know four or five steps on the negative side of the scale. So, when I got a call for this, I thought I really need to check it out because it is possible I could learn something.

I: Yeah well, we'll keep you updated with everything but yeah. And that's what we're hoping we're hoping. That this program will improve people's health, overall health. Yeah. Thank you so much. And I'm going to end the recording right now.

**Audio File Name: E-04 11-8-2018.mp4**

**Recording Date: 11/8/2018**

**Interviewer: AB**

**Transcriber: AB**

**Dates: 1/10/2019**

**File Name: #4 CKD Interview Emory University (11-8-2018)**

[00:00:00]

I: I'm just going to start the recording and so yeah, we can we can begin soon.

Interview recording number 4, Emory University CKD study.

I: Alright Mr. Name. The first question is can you tell me a time when you were physically active that you really enjoyed.

P: About two months ago.

I: Two months? And what is it that you did.

P: It was just with a work out group, at the gym.

I: What about it did you enjoy?

P: Just being around the people. That’s a really hard question.

I: Yeah. Sorry. A lot of the questions are going to go more into detail about your exercise routines. And so just keep that in mind. And the purpose of it is to just gain as much information for us so that we can actually create a good program that will fit you. So yeah. OK. So, two months ago you were working out at the gym and you enjoyed being around people. Is there anything else you enjoyed about it or was it more just like the group?

P: No, it was just the group.

I: OK. So the next question is, What do you think about when you hear the word exercise?

P: Physical labor ok.

I: Anything else?

P: [Inaudible]

I: What was that sorry, you are cutting out.

P: Healthy!

I: Healthy, okay. Healthy in which way?

P:Well if you exercise you get healthy... muscle tone, yeah.

I: Muscle tone. Yeah okay. So it keeps you healthy, okay.

I: OK. Anything else that comes to mind when you hear the word exercise?

P: Nah.

I: OK. All right. So, then the next couple questions are going to talk about your physical activity and exercise behaviors. And so, this first question is going to ask you about what your current exercise routine is or your most recent exercise routine. So, like what do you do when you exercise normally?

P: Mostly walking. Um, walking [inaudible] miles a day, around the neighborhood.

I: How many miles do you normally walk?

P: Two miles around the neighborhood.

I: How often do you do this?

P: Three or four times a week.

I: Is this your usual amount of physical activity?

P: Yes

I: Okay. And so you were saying that about two months ago you worked out at the gym and so do you. Do you work out at the gym as well, often?

P: About two months ago I was working out pretty frequently at the gym but I did not want to pay the membership.

I: OK. And so what do you normally do at the gym?

P: I get on the treadmill. I do about. Two miles on the treadmill. I do the stair climber for about maybe 15 minutes. I lift weights and then I am gone. That’s pretty much my routine.

I: Okay. How long are you normally there for?

P: Maybe 45 minutes to an hour.

[00:05:02]

I: All right. And when you did do this, how often during the week did you go?

P: At least four hours a week four times.

I: So you're pretty active person.

P: Yeah. I was.

I: OK… How did your exercise routine change when you were diagnosed with kidney disease?

P: Um I get really fatigued, got really tired. I didn’t feel like going to the gym. I didn’t like to do anything. I was always tired. And taking the medication take something out of you so. Yeah, and then I started getting really bad leg cramps and stuff like that. And it discouraged me from going back

I: Okay. When were you diagnosed with um kidney disease?

P: 18 months ago.

I: 18 months ago okay. Do you still have these leg cramps? Is that something that still occurs?

P: Yes.

I: You do. Okay. And what kind of medication are you taking?

P: Mostly high blood pressure medication [medication names, about 3 or 4]

I: So a lot of high blood pressure medication.

P: Yes

I: Okay… Has it gotten better since you were diagnosed or are you still feeling the same issues?

P: I am still having with the same issues. [mumbling, inaudible] I mean my blood pressure is better, a lot better. But um, I am still fatigued and stuff like that.

I: All right. Well we can move on to the next question. In your mind. What are some of the benefits of exercising?

P: Get healthy. Live longer.

I: OK. And what has motivated you to continue to be physically active?

P: Hello?

I: Hey.

P: I’m sorry, I kind of lost you there for a second.

I: OK. No worries. The question I asked what motivated you to be physically active or what continues to motivate you?

P: I want to live longer.

I: Anything else?

P: Nah, I just want to live.

I: I feel that. How important is exercise for patients with kidney disease. What's your opinion…

P: How important is it? I think it is very important. For kidney health, but more important is diet. I think diet… [background noise, inaudible]

I: So when you meet with your doctor and he talks to you about kidney disease management, does your doctor talk about exercise at all?

P: He tells me I need to be more active. And, stuff like that. I guess so, he does.

I: Does he tell you like specifically how long or like how hard you have to exercise or is it just please exercise more? Could you talk more about that?

[inaudible]

I: just exercise more?

P: Yeah.

I: Does he give you any advice on how to exercise more.

P: No, not really.

I: All right. Um, do you receive exercise advice from any of your health care providers.

[00:10:02]

P: No.

I: Okay All right. Next question, what are some things that make it hard for you to exercise?

And so I know you were talking about that.. [interrupted] Go ahead

P: Times makes it hard to exercise. Um, I mean I work and I got to support myself. I’m also pretty [fat?] So, I also, like I said I be tired a lot.

I: Alright so how often do you work. Like what are your hours like?

P: So I get started in the morning about 7:30. I get up and eat breakfast. My classes at 10:15. I’m basically in classes until about 2:15… [evening]. And then I work from 3 PM to 11 PM

I: So you go to class in the morning and then in the afternoon, you transition to work. Is that correct?

P: Yes.

I: Okay. And is this Monday to Friday?

P: Yes.

I: Okay. That is a pretty busy schedule.

P: Yeah, pretty busy.

I: OK. And so we've talked more a lot about like how you feel fatigued and tired. And so what would make it easier for you to overcome these barriers.

P: Oh.. more rest. [laughter]

I: More rest. Yeah. I’m guessing you have like extra work and since you're a student as well right?

Yeah. And what, what are you going to school for?

P: Kinesiology and Health.

I: OK cool. And that's part time?

P: No, I’m full time.

I: full time, alright. OK. All right um and so what about on the weekends? Do you have more time on the weekends?

P: Yeah.

I: OK. And so do those same barriers apply on the weekend, like time?

P: Uh not really. Pretty much free on the weekends.

I: Oh, all right. So we're about halfway there so thank you for continuing to do this. The next question is one of the things we are considering in our exercise program is group classes. And so I know that earlier you were talking about how you did a group class about two months ago. And so what do you like about these kinds of like group exercise activities and what do you dislike.

P: I like that the group is motivating each other to get through it. And I dislike the fact that um, there is not really anything I dislike about it, per se.

I: So there's nothing really that you dislike.

P: Yeah not really.

I: OK. So you like how you guys motivate each other. Who do you normally go to group classes with? Is it friends or just people at the gym?

P: Friends. And people I just met along the way while going to the gym.

[15:00]

I: And so how do you feel about exercise classes created especially for people with kidney disease. Is that something that you feel a little different about? Or is it the same kind of feeling?

P: Well I don't really want to generalize everybody. Like, that’s like saying I am putting everybody with AIDS in a particular class. I mean, I don’t feel like that’s right. But people need help, they need help. [laughter] You know what I am saying? That don’t make me feel too good. But if you need help, you need help. And I guess if you get kidney disease, I guess that is what this study is for. But yeah, it would be wrong if you put everybody with this.. in this class. You know what I am saying?

I: Yeah. I guess the question was more surrounding like being in a group exercise environment with people that are in similar situations as you.

P: I mean I don’t really have no business towards it. I mean I feel like, if everybody can like relate to each other, that would be a benefit. You know, I know what you going through.

I: Yeah. I get that. And so in your opinion how should we design these classes to be most convenient and appealing to you. So think of like the type of activities we could do. The location. The time. Like what would be your ideal kind of exercise program?

P: For me, it would be someone that is patient and be with someone, a group of people that is patient. And you know and motivate each other. Um, maybe..[inaudible]. It could be something on the inside, you know like in a formal gym setting, and some could be on the outside where we are just walking around and getting some fresh air. Walk in the park, you know what I am saying. That would be for me, you know.

I: So you like flexibility. So working out inside but also doing stuff outside. Um, for like the type of activity. So I know you said that you liked to walk and run and sometimes you lift as well. Would you like to do those type of activities is what I like to or anything else?

P: Uh, nah. Just track work and stuff. So, I wouldn’t want to be lifting weights. Like, I am almost 6 4 but I am 400 pounds, a little over. I am pretty sure.. I use to play football. I am not sure many people can lift as much as I can.

I: Ah, I see. So you would like to focus more on like cardio.

P: Yes.

I: Have you tried other types of activities for cardio besides walking or running.

P: No.

I: No Okay. All right. And so what would you value most from an exercise program like this?

P: I don't. I don't know. I don’t really know. I guess the knowledge about how to exercise better..

I: OK. Yeah. That's a good one. And so next question is how would you judge if the exercise program was successful, for you individually.

P: You said how? I’m sorry.

I: So like let's say you're participating in this program for three or six months. How would you judge think for yourself if the exercise program was successful for you? Oh man. [phone cuts out] Alright interview has stopped. Will call back. One sec in.

P: Hey. Sorry about that.

I: Yeah, we probably have maybe 10 15 more minutes so hopefully it won't cut out again. But the last question was how would you judge if the exercise program was successful? And so in your eyes, how would that be?

[00:20:34]

P: If everyone overall makes progress. Whether that is, where they could do 1 push up and now they are doing 4. Or you can do, where everybody sees some sort of progress. Like losing 100 pounds or 5. Progress is progress. As a group.

I: And so what about you individually. What is something that like a goal may you would have for a project like this.

P: I want to lose a lot of weight. [laughter] I want to lose about 20 pounds. Am I answering this question right?

I: Yes, you are.

P: So, getting down like 20 pounds.

I: 20 pounds. Ok. Yeah, I think that's definitely a good way to measure success. All right. So we have two more questions. This question is going to talk more about your experience with exercise instructors and so, do you have previous experience with like an exercise instructor.

P: No

I: Okay. If you could just imagine having an exercise instructor like how, like what could they do in order to help you be more willing and excited to exercise.

P: Make it personal. Be empathetic but make it personal as well. Everybody is not on the same page, and not on the same, you know. So you have to be empathetic and uh. Just be patient and work with everybody, even individually.

I: OK. I like that work with everybody individually. What would discourage you. Like what about them would discourage you from exercising?

P: Being too intense

I: Being too intense. OK. Anything else?

P: Not being patient. I mean, that is about it.

I: Okay. Let’s see. So for the last question here's a little background. We are, as part of a program we are looking at using a mobile phone app or mobile health phone app in order to track people's exercise program, progress outside of the classes. And so can you tell me about your past or current experiences with using a health app on your mobile phone?

P: I think is, I love mobile health apps. I love it. Its more informative. You know, keep track of your steps that you do a day. Keep track of your sleep habits. I had I think Runtastic was one. And it would tell you, like hey look, this how many steps you going, how many miles. And they give you compliments like good job, good job, like keep going, you can do this. Like they also had a water app. Like have you consumed your water today. You know, stuff like that. That kind of keep you [inaudible]. You know how to do it. Like how to be better physical and not so much counting calories. And also, I think one gave you recipes. Like hey, to achieve maximum, what you should eat is salmon, asparagus, vegetables. You know, it went into detail on how to be better. Motiviation. Also like motivation, like everyday. There are quotes, like quotes from Usain Bolt. You know, stuff like that.

I: Yeah. So an app that provides you with a lot of tangible and like useful information is important to you. Is that correct?

P: Yes, information.

I: So you used the run tactic app. Have you ever used like one of those watches or those fitness tracker trackers that you put on your wrists and it tracks you have you used one of those before?

[Phone cuts out again]

[00:26:01]

P: Hey sorry again. I think it is the weather.

I: OK. Well this is our last question. But the question I asked you is, have you ever had an activity tracker like some sort of watch device that would track your steps on things.

P: Nah. Only thing is like on my phone, like through runtastic and apps like that. I might have had like watch.

I: Do you still use runtastic? You still use that one?

P: Uh yes. Well, not no more. But it is still on my phone but I haven’t opened it up or tracked my steps like that.

I:What about the water drinking app?

P: Yeah I still use that.

I: OK. And also with the dietary habits one that how to eat better. Do you use that as well?

P: Yeah. My trial runs out like a week. They give you like one month free.

I: So what was the name of that app? Do you know?

P: It called Lifesome diet plan.

I: Okay cool. All right. So yeah we are definitely considering using some types of mobile phone apps to help you guys stay active. And so I'm glad you use these currently and so that was the last question. And so thank you so much for all the information you've given us already.

P: No problem

I: Do you have any other questions or is there anything else you would want. You want to share with us or talk about?

P: No. I’m cool. I am sorry my phone keeps going in and out.

I: No worries man. So no worries man. I can I can hear you loud and clear over here so. And we're recording it so we'll go over the information and I think we should be fine.

P: OK cool yeah. Thank you so much.

**Audio File Name: E-05 (11-16-2018) – Full Interview.mp4**

**Recording Date: 11/16/18**

**Interviewer: AB**

**Transcriber: BB**

**Dates: 3/22, 3/23 2019**

**File Name: #5 CKD Interview Emory University (11-16-2018)**

[00:00:00]

I: Alright so, 11/16/2018, um, interview number five. Alright so, first question is: can you tell me about a time you were physically active that you really enjoyed?

P: Huh?

I: First question is: can you tell me about a time you were physically active that you really enjoyed?

P: Uh, well three years ago when my toe got amputated.

I: Three years ago? And so, um, what kind of activity were you doing?

P: Walking, exercising, and going to the malls [inaudible], stuff like that, stuff I don't do now.

I: Ok, this was three years ago?

P: I'm used to going shopping and moving, doing stuff for myself, stuff like that. [Inaudible] and I can't now.

I: Ok. So, you used to walk, and exercise, and go shopping and that was how you were physically active?

P: Yes.

I: Ok, and um what did, what part of that did you enjoy? Was it just going out and shopping and also walking, or?

P: I enjoyed all of it.

I: All of it. Ok.

P: I could depend on myself, I didn't have to depend on nobody else to help me do that.

I: Ok.

P: You know people help me do stuff and sometimes you get [help?] sick.

I: Um hm, ok.

P: [inaudible] I'm not used to nobody doing stuff for me, like that. You can't clean and wash how I used to do everything for myself.

I: Got it. Ok so you really enjoyed being able to take care of yourself?

P: Yes.

I: Ok, um so then the next question is: what do you think about when you hear the word exercise?

P: Oh, I think it's a good thing.

I: Can you elaborate on that a little bit more?

P: Huh?

I: Can you elaborate on that a little bit more, um, so when you say it's a good thing like what does good mean?

P: You can enjoy this stuff once you get to moving around, meet other people, you know exercising and walking and you know, doing the different stuff, you meet friends on the track or at the gym, stuff like that.

I: Ok. Alright and so the next question, so it's gonna, it's asking about your current exercise routine, and so, do you have a current exercise routine?

P: No, sometimes my sisters, they go to the gym, and sometimes I go with them when they go to the gym. To [Plantnum Fitness?], they go to the track and I'll go with them on a walk sometimes and so somebody help me with some cause sometimes I won't be able to walk.

I: Ok, so how often does this happen?

P: I get tired sometimes from walking and stuff, then I have to sit and rest.

I: Ok.

P: It's the [principal?] cause it's all new to me, put it at three, well April will be four years, so...

I: Since you were diagnosed?

P: No, since my big toe got amputated and so I limp and I'm not used to have to sit down, wait on somebody else to do stuff for me, with somebody's help, do stuff for me, stuff like that. [inaudible] food, and then I have to stop and sit down cause I get tired. So, you know.

I: Ok, and so when you do go to the gym with your sisters or walk, is that something that happens like once a week, once a month, is there a specific...

[00:04:58.6]

P: I walk on like twice a week but uh lately I haven't been. I used to go twice a week, three times, one time, but lately I haven't been, cause I had to move out the place where I stayed at, I had to move in June [inaudible] you know I had to find somewhere else to stay. So that whole month I really [didn't] interested in doing it.

I: Ok, but now you're moved into a, a new place?

P: Yes.

I: Ok, that's good to hear. Um, and so did your exercise routine change when you were diagnosed with kidney disease? I know you were saying that your big toe was a big factor, but did it change at all?

P: Yes. Yes, we used to go to the gym, to the track all the time.

I: Before you were diagnosed with kidney disease?

P: Yes.

I: Um hm. Ok and so now not so much?

P: No.

I: Ok.

P: It's not cause of kidney disease, it's cause of the amputation of the toe, that's what it is I suppose. In the leg, I got arthritis in both knees and my back and in my hands.

I: Oh, I'm sorry. Ok, so next question is: what do you think are some of the benefits of exercising?

P: Uh, I think it's a good [inaudible] because it helps you motivate yourself, you know to move around and don't get stiff and don't stay up and [inaudible], stuff like that.

I: Ok, and so can you name some health-related benefits?

P: Huh?

I: Health related, so...

P: It can make your muscles and [inaudible] stronger, [inaudible] motivate yourself to be self-dependent and mentalize and um, it can help also physical.

I: Did you say mental?

P: I said it can help you from being depressed and stuff and it would help [inaudible] that's what I'm saying.

I: I got it, prevent you from being depressed. Ok, and so what, so I'm gauging from your answers that um, some motivations for you to be active are um, that it allows you to control your mental health um, also allows you to be more independent and it's healthy, um, or makes you feel stronger. So those are some things that motivate you to be physically active right?

P: Yes.

I: Is there, are there any other things that motivate you to be physically active?

P: Yes, like keep your weight under control, your eating habits and all that, yeah.

I: Ok.

P: I mean you learn from different stages and steps, when you go through different programs and stuff.

I: Um hm. Ok. Um, next question is: how important is exercise for patients with kidney disease?

P: Very important.

I: Very important? Um, so when you were diagnosed with kidney disease, did your doctor ever talk to you about um, exercise?

P: Yes, he did.

I: Um, so they did. What exactly did they say, do you remember?

[00:09:52.0]

P: Yes, he told me uh, even though I'm gonna walk and stuff [inaudible] therapy come to the house and work with me. He signed me up [inaudible] [come?] and um, he's working with my case worker, but every time they [inaudible] never show up. So, they told me while I be at home I should try to go through as much movement with my legs and arm and waist and stuff as I can. Grab me some pounds or stand up.

I: So, your doctor was, played a pretty active role, right, in trying to motivate you to exercise, is that correct?

P: Yeah.

I: Ok and so, did he give you any specific kinds of exercise advice, like how...

P: [inaudible] like uh, when I sit down move my arm, give my leg [lifts?] and stuff.

I: Ok so lift your arms and legs?

P: Yeah and how to get a [inaudible] and bend, back, foward, and side, stuff like that.

I: Ok, bend. Ok um, so next question um, is asking: what are some of the things that make it hard for you to exercise? And so, I know we talked, you talked a little bit about this, but could you um, go over it again?

P: Sometimes I be tired from the arthritis and the kidney disease or the swelling in my leg [inaudible] come from the um, [inaudible], that stuff. And um, the pain from my uh, my leg from where I [limp?]. Other than that [inaudible] I'm not [sweating?], I'll be alright.

I: Ok. So, the main barriers for you are health related?

P: Um hm.

I: And so, ok. Would, would time, is time ever a barrier for you?

P: Uh, no cause it comes, the pains they comes and goes, different times. It ain't just when it's cold, sometimes it be hot and I still go through pain, [inaudible].

I: Ok.

P: They just, it, I mean they vary, it comes and goes.

I: Ok.

P: [inaudible] control it.

I: And so, um, what would make it easier for you to overcome this barrier? And I'm seeing that it's more health related um, what would make it easier for you?

P: Um, it might, get somebody to help me.

I: Get somebody to help you? Ok.

P: Yup [inaudible] somebody, [inaudible] or something, maybe more.

I: Ok. Alright, um, so we're gonna move on to a different section of our interview. And so, this one is gonna actually talk about um, the, the possibility of um, having a exercise program. And so, one of the things we are considering is an exercise program that's group-based. And so, how do you feel about group exercises in general?

P: Oh, it's alright.

I: It's alright?

P: Um hm.

I: Ok so, so you like them?

P: Yes.

I: And so, what about them do you like?

P: Huh?

I: What about group exercises and group classes do you like?

P: Cause it's more fun and more motivating.

I: More fun and motivating.

P: [Inaudible] have more people and that make you, lift you up with spirit [inaudible] do you know, wanna do and wanna [inaudible] see others, not just [inaudible] other people like you, they be interesting and wanna do it too.

I: Ok. So, you like it because it's fun and motivating and you like being around other people because it lifts your spirits.

[00:15:02.9]

P: Yes.

I: Ok. Um, how do you feel about an exercise class that's made especially for people with kidney disease?

P: I think it's alright.

I: Think it's alright? You have the same opinions about it too?

P: Yes.

I: Ok. Is there anything that you dislike about group classes?

P: No.

I: No? Ok. And so, um, are there any barriers for you to participating in group classes such as transportation or time?

P: Yeah, I don't drive so I would like schedule for someone to pick me up.

I: So, you don't drive you need someone to pick you up, ok. Ok, anything, no time barriers?

P: No.

I: Ok. Alright and so, next question is: how should we design these classes to be most convenient and appealing to you? So, more specifically, like what type of activities would you like to do like in order to exercise?

P: I would like to be able to move around and get around and stuff [inaudible].

I: Get around.

P: It's [inaudible] doing all the activities and stuff like I...

I: So, like walking, would you rather walk, would you rather run, would you like to...

P: Walk.

I: Walking. Any other kinds of activities that you enjoy?

P: I used to like sit and squat and stuff like that.

I: Sit and squat, ok.

P: But I don't do it no more because of the knee.

I: Ok.

P: Cause like [inaudible].

I: Ok.

P: [Inaudible] sit and squat at this point.

I: Can you, can you still bike?

P: Can I like sit on the [inaudible] and stuff and I can't do it anymore.

I: You used to sit on what, sorry?

P: On the floor, sit and stretch.

I: Ok, and stretch, but you can't do that anymore?

P: Nuh uh.

I: Ok. And so, in terms of a location, so a location in Atlanta, we were thinking about having the exercise programs at the hospital here. Um, is that something that would be convenient for you?

P: Yes, I did have to call the transportation people uh, three days ahead.

I: Three days ahead?

P: Uh huh.

I: Ok. Ok, and so, what would you value most from an exercise program?

P: I think I would value a lot.

I: Um hm. Anything specific?

P: No.

I: No? Ok. Um, alright so we're, got three more questions. So, next question is: how would you judge if the exercise program was successful?

P: I would rate it excellent.

I: What was that?

P: I would rate it excellent.

I: You would rate it excellent? So, would there be any like indicators for you that the program was actually working for you?

P: Yes.

I: What would those indicators be?

P: Uh, [coaching?] and be motivated and happy more and exercising and self-control and stuff. [Inaudible] physical.

[00:20:03.3]

I: Ok. So, if the exercise programs allow um, if the exercise program is successful in making you more motivated and improving your self-control, that's something you would say would be successful?

P: Uh huh, motivating for myself yeah.

I: Any, any like physical like um...

P: And physical.

I: Ok.

P: Doing stuff for myself and make legs and arms and body's feel stronger.

I: Ok so, if you got stronger that'd be a...ok. Um, and how would you um, how would you judge if the exercise program was successful for everyone in your group class?

P: It would be great.

I: Um hm, do you, how would you judge that?

P: I'd judge as excellent.

I: So as long as everybody is also feeling um, motivated and stronger?

P: Yup, moving around and stuff like they want to.

I: Ok. Ok um, so for our group classes we are also planning on having like an exercise instructor to help um, guide the class and so, what um, for you, what could the exercise instructor do to help you be more willing and excited to exercise?

P: Working with me towards being healthy and physical-wise.

I: Um, and so, if they were, if they work closely with you, if they worked...

P: Closely, and sometimes you know, that helps motivate you and stuff and push you to go forward.

I: Ok, so you want someone that's motivating.

P: Uh huh and in control, self-controlling, you know, sometimes you need a little push [inaudible].

I: Ok. Um, and what things would discourage you from exercising? Like what, what would, what are things that they would do that would discourage you?

P: Like not um, I don't know, cause it's hard for me to get you know, mad at somebody.

I: Um hm, I wouldn't say necessarily mad, but like what would, what would, is something that they would do that would make you less willing or excited to exercise?

P: I think so.

I: Ok.

P: Cause I'm [inaudible].

I: You're sorry, what was that?

P: It take, it takes a lot for me to get mad with somebody or [inaudible] somebody.

I: Ok. Um, and how helpful is goalsetting for, when you exercise? So, do you set goals, or would you set goals in this program if um, if you were to participate?

P: Yes.

I: So, like, I know some people, their goals are to be healthier, some people's goals are to lose weight, some people's goals are just to feel happier. So, in your situation, how would you, what would your goal be?

P: All the above.

I: What was that?

P: All the above.

I: All the above, ok. Ok, so now this is the last question. And so, we are considering using a mobile phone app as part of our program, can you...

P: Huh?

I: We are considering using a mobile phone app as part of our program, can you tell me about your past or current experiences with using a health app on your mobile phone?

P: I've never had one.

I: You've never had a mobile phone app/exercise app?

P: No.

[00:25:00.0]

I: You're familiar with using apps though on your phone, right? Just not an exercise one?

P: No, I haven't used too many. I can receive but I don't text and all that.

I: Ok.

P: [inaudible] [speaking to someone else] Yes, I'm back.

I: Ok. Is there a reason why you don't use the phone or the internet? Is there a specific reason?

P: [inaudible] [speaking with someone else] Hello?

I: Ms. Name you still there?

P: What'd you say?

I: Is there a reason why you don't use apps or internet, is there a specific reason?

P: I, I don't do [inaudible] and stuff.

I: You don't, you just...

P: My niece tell me, they do all that for me.

I: Ok, so your family...

P: They go online for me and all that stuff like that.

I: Ok.

P: And my daughter and my son, they do all that.

I: Ok. Ok, well um, that was the last question, and so, um, thank you again Ms. Name for taking the time to talk with me today, like I really appreciate it. Um, and so you have given us a lot of helpful information and we're gonna take it into account when we're creating our exercise program. And we will continue to keep you updated on um, on that. And so, before I leave is there anything else that you want to share with us, talk about, or clarify?

P: No.

I: No? Ok. Well thank you so much Ms. Name and I hope you have a great rest of your week and a great weekend.

P: Thank you, you too.

I: Alright buh bye.

P: Um hm.

[00:27:08.4]

**Audio File Name: E-06 12-9-2018.m4a**

**Recording Date: 12/9/2018**

**Interviewer: AB**

**Transcriber: BB**

**Dates: 3/13/2019**

**File Name: #6 CKD Interview Emory University (12-9-2018)**

[00:00:00]

I: Alright, and our first question is, um, a little introductory question so, can you tell me about a time you were physically active that you really enjoyed?

P: Um, I would say probably around 1997.

I: 97? Ok, and what were you doing in 97?

P: I was totally physically active, that's when I first started working at the YMCA.

I: Ok, were you a trainer?

P: No, an employee.

I: Oh, ok. And what activities did you do at the YMCA and why did you enjoy them?

P: Um, I was actually working out, lifting, and it was something that made me feel good and look good.

I: Ok, working out, lifting, and it made you feel and look good?

P: Yes.

I: Ok.

P: Young and vibrant.

I: How old were you back then?

P: Oh, I don't know, let's see 97, probably like 30 years ago, no it wouldn't, 20 something years ago, so probably 30 something, early 30s.

I: Ok, early 30s. And remind me when you were diagnosed with CKD?

P: Um, oh, probably about 2011 maybe, 10/11?

I: Ok.

P: It probably was before but I'm just estimating probably a little bit.

I: Ok. Alright, um, next question would be: what do you think when you hear the word exercise?

P: I need to.

I: You need to. So, anything else that comes to your mind?

P: Physical fitness.

I: Physical fitness.

P: Good health.

I: Good health.

P: Structured.

I: Structured.

P: That'd be it.

I: Ok. And when you say, "I need to", why do you say that?

P: Cause I don't really, I move, and you'll see I move a lot, but it's not structured.

I: Ok.

P: So, it's not really, it's not really exercise, if you know what I mean.

I: Yah.

P: Just participating [inaudible], which is fantasy probably.

I: Ok. Alright.

P: Like for example this week I'm a probably blow your mind and try out, but I'm not exercising at all.

I: Because?

P: Um, I probably won't even, well this week I got a lot going on, so it's like I'll just start, you know going. That's why I say I need structure, it's like if it's not structured, I'm not gonna do it. Yeah.

I: When you say going, are you saying you're walking a lot?

P: Uh, I'll be walking a lot. I volunteer a lot and it just requires like, I'm moving. Um, if you considered, like, movement of your body, a constant movement of your body exercise, I do that. But, you know, I don't consider it. [inaudible] I'm a walk, I'm constantly walking, just cause I'm hyperactive. So, that's why it's like I sit down maybe, five, ten minutes I'll be getting back up, and find something, you know do something, and it [inaudible], I'm just real hyper.

I: So, it's not like you’re going to a gym and working out, you're just, in your daily life, you're just moving a lot and that's kinda how you exercise?

P: Yeah.

I: Ok.

P: Why I don't, don't say exercise, I just move.

I: Just move. Ok.

P: I'm not exercising at all, that's movement, that to me, that's just movement. I'm not, you know.

I: Ok. Alright, thank you for that, let's move on. So, the next question is talking about, um, your current exercise routine, and so, I'm, do you have a current exercise routine?

[00:05:12.9]

P: No.

I: Ok, so, um, how about you just tell me about um, like your average day, cause it sounds like you walk a lot, so just tell me like um, you're volunteering, are you walking, like how far are you walking.

P: Well, it's like paces more so, it's like ok, um, like [inaudible], what no let's say this, I got to sleep in on Saturday. And um, actually two events on Saturday. One is a toy giveaway for um, kids, that I sponsor, so like it's at the Y, so I'll be at the Y, walking around. Potentially the whole time, walking, you know trying to get this, putting out this, putting out this fire, seeing this kid gets that, that, that, then I'm gonna leave there and I'm a come home. Then when I probably come home since I was up so early, I'll probably clean up the house, I'll be walking around cleaning up. And then I have to go to a friend’s uh, birthday party, so I'm gonna leave again. So, you know it's like that kinda activity.

I: Ok.

P: Oh I do stuff with the seniors, so I might um, have an event that I give out food for them, so that's like twice, that's twice a month, so when I'm there I'm walking around, talking to them, getting food, you know packing, stuff like that.

I: Ok.

P: It's just like, as far as like blueprint, it's hard for me to sit down too long, cause I feel stiff and stuff like that too cause I am getting toward an arthritic stage.

I: Ok. Alright, and so do you volunteer every day during the week or just?

P: Basically.

I: Basically, every day.

P: Just about, we would say, um, I will at least give you an hour probably every day unless I'm tired. If I'm tired, I have to take, you know because there is so much going on in my body, I have to take that time too, and that's one thing I do you know, take that time, that's why I would rather have structure. You know exercise, cause I think it would help me in that aspect.

I: Got it. Um, ok and so I'm guessing you, you drive to these places and then you walk, you walk around?

P: Yeah.

I: Ok. Alright. Ok, so how did your exercise routine change when you were diagnosed with kidney disease?

P: Hm, my pattern would never change with any diagnosis. My pattern changes with time, it started getting busier at work and stuff like that, it didn't stop me from doing anything.

I: Ok.

P: I just had that, just as far as like work and stuff, it was like I just would you know, I was a workaholic, so I just kind of like to be on, you know I just went on and kept.

I: Ok so, so work was the biggest, the biggest changer when it comes to your exercise?

P: Yeah, exactly. Exactly, cause they even tried to do it where we could work out for an hour, cause it's for [worldwide?] employees, but it didn't even work, cause we were working so much. And then you know at the end of the day, you trying to get out of there. [Laughter]

I: Um, when did you retire?

P: In 2013.

I: 2013. Ok. Ok, so. So now the next couple questions are gonna talk about the benefits of exercising, and so trying to understand like what you would perceive as a benefit of it. And so, what are some benefits of exercising in your opinion?

P: Living longer.

I: Ok.

P: More productive life. Healthier life.

I: Ok.

[00:10:05.0]

I: Could you go into more detail a little bit on healthier life, like what about exercise is healthy, in your opinion?

P: Um, as far as my breathing ability, since I have a, I have a heart condition, so it would help me in that aspect. I have, one of the issues I have with inclines, so um, it would help me with doing the inclines and stuff.

I: Ok.

P: And um, therefore helping my you know, as far as my heart beat better and you know that type of [inaudible]. And also, too I have arthritis, so that would help the aspect of um, my arthritis too, as well. I'd tone it down some for you to help me be able to deal with it more.

I: Ok. Um, so what motivates you to be physically active?

P: Right as of now, nothing. Um, but I would say the um, will to live, to be healthier, cause I have it in my head it's just, I got to, I again, I need structure, I need to slow down. Have like structure.

I: Ok.

P: If you hear some noise, I'm kind of cooking while I talk to you.

I: Yeah nope, no worries. As long as you’re not like blending up a smoothie or something.

P: No, no, no.

I: Um, so I kinda wanted to dive in to this a little bit more so, when you say structure, you're saying like, like a...

P: A structured activity, George you're here 11 o'clock to [inaudible], dah, dah, dah, dah, a trainer activity, let's say that.

I: A trainer activity.

P: That would be more so.

I: Ok, trainer activity, at a specific time of day, every day?

P: Exactly.

I: Ok.

P: Specific time, on the same day, all yeah, same time, same day of the week, every week, every month. [Laughter] Like that.

I: How many times a week would you be more willing to do?

P: Two to three.

I: Two to three.

P: Probably three.

I: Ok. Cool, thank you. Um, so how important do you think exercise is for patients with kidney disease?

P: Uh, I think it's important for everybody with health issues.

I: Ok.

P: I think especially at the point of dialysis or potential dialysis or what have you, I think it's all important because I'm told dialysis wears you out so, the stronger you are, the better.

I: Uh huh. Ok. And so, when you go to your doctor, does your doctor talk to you about exercise, um, and kidney disease management?

P: Yes, my doctor tells me [inaudible].

I: So, what exactly does your doctor say?

P: Um, it's up to you. If you want to live, you gotta listen to what I say. [Laughter] If not, you're gonna be on dialysis, that's what my doctor says. [Laughter]

I: So, is that in relation to exercise or just living a healthy life in general, like?

P: Living a healthy life in general.

I: Ok.

P: And not, um, again it would be, again I think can kind of tie that into structure too and not listening to, you know, someone per se that does not know. You know cause I was the one who would go on to the internet and all that dah, dah, dah. So, you need somebody who's involved in that, so.

I: And so, do they, does she give you any exercise advice like, work out this many times a week, or?

P: No.

I: No.

P: I would say no.

[00:15:00.4]

I: Ok. Next question is: what are some of the things that make it hard for you to exercise? And I know you touched upon this a little bit.

P: No structure.

I: Structure, no structure.

P: That's it. That's the only thing that keeps me from going.

I: Ok. I also know you talked about um, like, certain health issues, so like you have a heart condition and arthritis, would those.

P: I have a heart condition, I have arthritis, I'm HIV positive, um kidney disease, um, I said hypertension.

I: [Laughter] No, you didn't.

P: I have a lot going on.

I: Ok.

P: I have a lot, for a long time.

I: Yeah. Ok so, one sec. So, structure. What about time?

P: I'm sorry, say, what'd you say?

I: Um, since you retired, I'm assuming that time isn't the biggest factor?

P: No.

I: No, ok.

P: No. I can always find a time.

I: Um, ok. Ok, so next question is: one of the things we are considering, and you know this already, in our exercise program is group classes. How do you feel about exercising in groups, in general?

P: In group classes, oh it doesn't matter. That's fine. Now, I have no problem with group, um, will they be at the same pace, or you know, I would, if, with groups it's fine but it's like, still need individual, if I'm set at a pace that the other class is going at, I might get a little frustrated. You know what I mean? If everything, if I don't have the rules they have, ok you're getting on the treadmill, or you do this whatever for an hour or whatever, as long as it's no wait time like, if um, say for instance if somebody tells you ok you do ten pushups, everybody do ten pushups, if I finish before they get to three, I'm gonna get bored. You know what I mean?

I: Yes, so you want to kind of be challenged too?

P: Yes, yes, I have to cause if not I'll [inaudible]. And I'm also ADD. I didn't tell you that part. And dyslexic and then well, but um, I got it all under control.

I: Well, looks like you're doing well though. Um, ok so you have to be challenged, you need, you need your own, you need like some autonomy, right? Is that what you're saying like?

P: Yeah.

I: Ok.

P: Uh huh, exactly. I can be in the same room with them but, you know, with the other people, but again I've got to be able to do if I get, if you know, somebody else [inaudible] I've go to still be able to [go?].

I: Yeah, I mean that's fair. Um, let's see, what do you like about group classes?

P: Um, I'm a group person, it's just, it's more supportive.

I: More supportive.

P: You know? And that's the [inaudible] thing, you have people around that are trying to accomplish the same thing that you are, hopefully. Um, it all works well. And, no wimps and stuff.

I: No what?

P: Wimps.

I: Wimps like?

P: Yeah. Complaining.

I: No, complaining, ok.

P: Yeah, I can't take the complainers.

I: Are you deep frying stuff?

[00:19:59.5]

P: Um yeah, hold on let me [inaudible]. No not frying.

I: Not deep frying just?

P: I [started?] some bacon.

I: Ok.

P: Ok, did the volume go down now?

I: Um, yeah. It was just a little um.

P: Yeah, I had the [inaudible] up real high.

I: Yeah. That's good now. Um, ok so no complaining but these classes are more supportive in your opinion. Ok, um do you have any barriers to participating in a group class?

P: No.

I: Such as transportation or time? Ok.

P: No.

I: And you have a car?

P: Yes.

I: Ok. Ok, so, we touched upon this already but, so if we were to design an exercise program, um, what kind of activities would you like to do, um...

P: Ok, I need to work on inclines.

I: Inclines, so like, need to work on inclines.

P: Walk up. Walking up, yeah. Um, let's see what else? Um, well treadmills incline but treadmill focusing in on inclines.

I: Ok.

P: Let's say that cause that's one of my greatest things going up and down. Um, you know, going up, like up hills and stuff, I'm very slow at that. Um, building muscle.

I: Building muscle.

P: Building muscle, I'm a small person so I think it's time to, once I lost weight when I got sick, I lost 30 pounds, and that was in 2013, never been able to get it back. So, I want some weight out of this deal too.

I: Ok. So, you wanna gain weight.

P: I wanna gain weight out of this deal too.

I: Ok.

P: At least 10 pounds.

I: At least 10, ok.

P: And I eventually do have to go on dialysis, I ain't got nothing to [inaudible] my own body.

I: Can you say that again?

P: I'm so small. I said I'm so small they'll need my whole body in the [inaudible] if I have to do dialysis.

I: Ok. So, ok. So, in terms of location and time, in the mornings that's what you said right?

P: Um hm, yep.

I: And on the weekdays.

P: Uh huh.

I: Ok, um. So, what would you value most from this exercise program?

P: Good health.

I: Good health, ok, ok.

P: And I could, I would say also um, gaining more knowledge of um, my disease, that particular disease and how to um you know, kind of manage it through exercise.

I: Ok.

P: Pancakes [inaudible].

I: You're making pancakes too?

P: I'm sorry?

I: You're making pancakes too?

P: I know.

I: That sounds good.

P: [inaudible] [laughter]

I: Ok, would you want to learn more about like your specific disease while you exercise? After you exercise?

P: While.

I: While? Ok.

P: Yeah.

I: Ok. Um, how would you judge if this exercise program was successful?

P: Um, if ya'll had me exercising regularly. [laughter] If you had me exercising regularly and I feel better upon completion. I know it's not that long, but I feel, you know, I feel a difference.

[00:25:00.8]

I: Ok, um. And so how would you judge if the program was overall successful, so like everyone in the program, like how would you judge if that was successful or not?

P: Um, if everybody in the program leaves out with a new way of thinking. A more positive way of thinking and a more positive way of living their lives.

I: Um hm.

P: And they follow it in the follow-up, I'm not sure some of you all do so many month follow-up or something like that? And um, if they're still engaged.

I: Ok. Got it, so follow-up's important.

P: Yes, definitely.

I: Ok, got it.

P: And it's encouraging too cause it, they're gonna need you all to follow up and say "Johnny, what's going on? You working out Johnny?" You know. [Laughter]

I: Ok. Alright, so we got two more questions. Um the next question is: what is your experience with exercise instructors?

P: Um, and its group instructors or individual trainers? Cause my experience with...

I: Group.

P: Um, I like them if they're wound up.

I: You like them if they're wound up?

P: I like them if they're wound up and participating.

I: Ok.

P: Like by me being at the Y, like our instructors are like wound up. You know they're participating, they're encouraging, they're motivating, they're not just instructing.

I: Ok.

P: You know, they're actually participating in front of the group, you know?

I: Yeah, so they're pushing you.

P: Exactly.

I: So that's a good instructor to you.

P: Exactly.

I: Ok. Ok.

P: Not one of those that, when class is over, they're the first one to grab the bag and...

I: Ok. So, you want the exercise instructor to be committed and engaged in the exercise program?

P: Yeah, and motivated.

I: And motivated, ok. So, how helpful is goalsetting for you when exercising?

P: Say it again now.

I: How helpful is goalsetting for you when exercising?

P: Oh, yeah. Great. Yeah, it's necessary for me to have them.

I: Ok. And...

P: I have to always have something to reach for.

I: Ok.

P: A challenge. My life is, that's why basically how I live, by challenge, my life is a challenge. If I do something, I gotta do something else more, do something else better. You know what I mean?

I: Yeah.

P: But um, and I'm sorry to say, "you know what I mean", it's what I say to my sister, she says no I don't. [Laughter]

I: I do know what you mean though.

P: Ok.

I: So, what things would discourage you from exercising? Or like what things would the exercise instructor do that would discourage you from coming back, you know, from exercising? I know you said if they like seem like they're wanting to leave right away.

P: Yeah, not motivating, not assisting. You know, um, understanding, you know, the levels of the people in the class per se, you know, there's Johnny and Johnny can't really do it, you know too much, motivate him to do it but don't make him feel bad cause he's not doing it.

I: Ok, got it. So be like compassionate.

P: Exactly.

I: Ok. Do you know somebody named Johnny?

[00:29:55.1]

P: No, nuh uh. [Laughter] It's one of them kids Johnny you know what I mean, explain to the kids, for some reason I always use Johnny. I have a cousin named Johnny perhaps that's some reason. I guess when you younger you hear those books and they always have Johnny.

I: Yeah, they really do. Or like in those movies? Johnny!

P: Johnny!

I: Yeah. Alright so, we are on the last question. Um, this question is just gonna talk more about mobile phone apps. Um, can you tell me about your past or current experiences using a health app on your mobile phone?

P: Um, I had a slight one. I did two, like a water one, one day.

I: A water one?

P: They had the water one, cause you have them where you drink water all day and then um, what was it I did? Well I wore my tracker for a little while, um when I first got sick. Um, and there was some other and that I had that had like a heart with a light in it or something through it. That was years ago though but no, not really, let's say no, I haven't.

I: So, you said you used to use a Fitbit, right?

P: Um hm, I didn't use it that long I still have it.

I: Well so what, what happened why'd you stop using it?

P: Well I had actually gotten it for, to monitor my um, heart rate and pulse and stuff, when I first um, got sick. Cause I have like a, I have a low um, heart rate, my heart rate is low. And that's due to like the medication and stuff, my heart rate gets low in the 60s, you know, depending. And that's actually supposed to be like a runner’s heart rate, so I was trying to make sure that my heart rate and everything was staying constant for a while. Cause I was in and out the hospital, in and out, in and out, in and out. But um, when I finally mellowed off, it did too.

I: Ok. And so, then after you didn't need to track your heart rate that's when you stopped using the Fitbit?

P: When I felt like I didn't need to.

I: Ok.

P: [Laughter] Yeah, when I felt, not when I do what I should, when I felt like I should stop. Just should've kept wearing it.

I: Ok. Um, so what did you like about it, what didn't you like about it?

P: The Fitbit?

I: Yeah.

P: That's what, you know actually, that's what I was just thinking in my head, you all are doing really good, see this is saying that I really wanna participate, cause I have this on my arm. That's what the Fitbit was about. The constant on my arm.

I: Um hm, and you see it all the time, kinda thing?

P: Uh huh, and I want, I don't really, I can only wear jewelry for a little while. Cause everything seems to feel heavy on me after a while.

I: Ok.

P: But I'm a keep the Fitbit on, I've had it on since [Laughter]...

I: That's good.

P: I was looking at it, you know must really want to participate.

I: Um hm, ok. So, you like how it's always just there?

P: Yeah, it's yeah.

I: Ok. Um, let's see. Ok. Well I think that's about it, um, thank you again for agreeing to be interviewed and you have given me like a lot of helpful information that I know will be helpful in the future for other people. Um, is there anything else that you want to share with us, talk about more or clarify?

P: I had a question, are they gonna touch anywhere on nutrition anywhere?

I: Um, I think they may, um, but if not, we can definitely like incorporate that.

P: Because, well the thing about it is, is if they're not eating healthy, the exercise it's kinda like both of them go together so if they possibly could, cause I'm really helpful on the help, I mean the food so you know, knowledge wise, say we eating the right food, knowledge wise. I'm frying bacon, you know, just in that case, they're not eating healthy they're coming and trying to work out, they're stressing their bodies more.

I: Yeah, no that's definitely true and so we can look into that a little bit more.

P: Ok, alrighty.

I: But um, thank you again, George, appreciate it. Um, before you leave, um I'm gonna stop recording right now.

P: Ok.

[00:34:50.3]

**Audio File Name: E-07 12-20-2018.mp4**

**Recording Date: 12/20/18**

**Interviewer: AB**

**Transcriber: BB**

**Dates: 3/23, 3/26 2019**

**File Name: #7 CKD Interview Emory University (12-20-2018)**

[00:00:00]

I: So, this is CKD Study interview #7. Um, and so our first question for you um, is um, can you tell me about a time you were physically active that you really enjoyed?

P: Well um, let's see, probably up until I was around 50, after that it uh, it started slowing me down.

I: And so, what did you, what kind of exercise were you doing at 50?

P: Well you know actually I would go to, sometimes we would go to [church?], with a group of seniors and go to the Y, use the treadmill and walk on the uh, track.

I: Oh cool. So um, you would walk with other people?

P: With other people, sometimes uh, alone because you know, sometimes you don't find anybody, but you can just go and you know, do it yourself.

I: Yeah, ok so you would do a lot of walking or is there any other forms of exercise that you would do?

P: Um, no not really. Well, sometimes we uh, I was in a class where we had um, this elastic band you know, and I've done Zumba. I've done Zumba and...

I: Zumba, ok.

P: Yeah, and so you know, putting on the elastic band sometimes and uh, the uh, we used to have an exercise class where I live, once a month. And uh, I participated, it would be different things, you know, stretches and that kind of thing.

I: Ok. And so, this was when you were in your 50s?

P: Yeah and later on, once I moved to the senior community.

I: Ok so, um, ok, that's a good amount of information. Um, the next question is then asking you um, what do you think when you hear the word exercise?

P: Uh, [I hear running?] fast, I hear um, cause I need to do more of it.

I: Yeah.

P: Uh, it's uh yeah. It's uh, because I've gotten, you know, I sit around too much here lately, and I need to do something uh, to get more exercise and that's really why I joined the study because, cause I could get uh, some kinda routine [inaudible] alone cause a lot of times things alone you won't do it.

I: Ok so, um, so right now you think, whenever you hear it you think that you need to do more exercise. Is there anything else that comes to mind when you hear the word exercise?

P: Um, let me see, uh, I don't know uh, a lot, I think sometimes it takes a lot of effort to do it.

I: What was that, sorry?

P: A lot of effort to do it.

I: Oh, a lot of effort. Ok.

P: [inaudible] and uh, effort. Exercise on a regular basis, uh huh.

I: Um hm. Ok, yeah that, I think that's pretty common. Um so, ok, the next question is asking you about your current exercise routines. So...

P: Uh, ok. Go ahead.

I: Do you have a current exercise routine?

P: No, I don't.

I: Ok. Um, so in, since you don't have one, um, in what ways are you like, able to be physically active during your week? Um, and so that could like include like, do you go walking during the week, um, do you go to the grocery store and do you walk around, do you um...

P: Oh, well yeah, I go to the grocery store, the other thing is uh, sometimes like the first week after I came for my initial, for my orientation, after that I couldn't walk it off because I, sometimes I have this flare up of arthritis in the right foot.

I: Oh no.

P: And I can't put my shoe on so, but it'll [inaudible] until it calms down.

[00:05:00.4]

P: So that kinda just, that kinda stops me sometimes.

I: Ok, the arthritis in your right foot.

P: Uh huh.

I: Ok, and so, that's kind of a barrier um, but you did say that you do go walking when you go grocery shopping or um, do you shop, do you like do just like shopping at the mall, or?

P: Well, I mostly go grocery shopping. Sometimes every [random day?] I go to the mall.

I: Ok.

P: I’ve never been the person to hangout in the mall, I just, you know, if I go to the mall, I know what I'm going for, I go get it and I leave.

I: Got it, got it. And so, are there any other times that you just normally walk in your week um, things that you do?

P: Um well, uh, sometimes, you know, if the weather is, it’s not too bad, I like walking outside, you know.

I: Walk outside, ok.

P: Right, I like walking outside and um, if the weathers not to bad and sometimes we have people that be on a walk and um, they've opened, they uh, a street, I haven't been over there yet, but they have a senior program, a exercise program for seniors.

I: Um hm.

P: Uh, I'm going over there, that's one of my um, resolutions. I don't make resolutions cause I never keep them. Uh, [laughter from both] so next week I'm going over and register.

I: Ok.

P: Real fast. They have a walking track, they have water aerobics, I don't do water aerobics, I don't do swimming pools.

I: You don't do swimming pools, ok.

P: But uh, they have chair exercise and they have the track.

I: Ok.

P: And uh, I think they have a fitness instructor over there.

I: So, you're going to enroll in that?

P: Go and join, I'm gonna join, yeah, I'm gonna join that senior group over there.

I: Ok, cool. Um, alright, and so, let's see, next question is: how did your exercise routine change when you were diagnosed with kidney disease, if at all?

P: Well um, I think it did in the fact that, and I'm not sure it's just because the kidney disease so that's, I've had several other medical problems all at once. I got congestive heart failure and diabetes.

I: Ok.

P: And kidney disease all at the same time.

I: Oh, wow.

P: And sometimes, the medications, and I have high blood pressure, and sometimes the medications, you know, make you really tired.

I: Um hm.

P: And um, so um, having all that going on, I also have a uh, I'm anemic.

I: Ok.

P: So, I have to take iron tablets and stuff like that.

I: Ok.

P: And a lot of times I just have to force myself to go do whatever it is I'm gonna do.

I: Um hm.

P: Yeah.

I: Ok, um, so it wasn't just the kidney disease, it was like uh, a multiple, a multitude of factors?

P: Right.

I: Ok.

P: But the medicine, my heart failure and I don't have any problem with that anymore. As a matter of fact, I haven't seen my cardiologist for a year, she says my heart is in pretty good shape now.

I: Awesome.

P: And my uh, my diabetes is under excellent control.

I: Ok.

P: My A1C went down from 8 to 5.3 or something like that.

I: Oh, that's really good.

P: Yeah, so it's, it's better, you know, yeah.

I: Alright, so I'm glad to hear that you're doing better. And so, um, maybe some of those barriers will not be as bad anymore. Um...

P: Right.

I: Ok. So, the next question um, is gonna talk more about what are your, what are the, like what are some of the benefits of exercising, in your opinion?

P: Uh, some of the benefits of exercising in my opinion is uh, I think that I will, I'm hoping that I will sleep better. I will probably, I have problems sleeping too.

I: Ok.

P: I hope I will be able to sleep longer, you know, I wake up in the middle of the night and, and um, I have terrible, terrible habits sleeping, yeah.

I: Ok.

P: And I'm thinking that if I exercise, it should help me.

I: Um hm.

[00:09:54.0]

P: Uh, I wanna say be tired, more tired, maybe that's what I need to make, to wear myself out and then I can sleep better, I don't know.

I: Um hm.

P: Uh huh.

I: Ok, so um, that's one of the benefits of exercise, is to sleep better. Anything else in your, in your opinion?

P: I'm sorry?

I: Are, do you, are there any other um, benefits to exercising in your mind?

P: Of course, I'll lose weight, I'm sure.

I: Ok, lose weight.

P: Uh huh.

I: Anything else?

P: Hm, no I think that's, as far as I am concerned, I think that's two major things right there.

I: Ok.

P: Uh huh.

I: Yeah, perfect. Um, let's see. And so, one second, sorry. What normally motivates you to be physically active?

P: Well um, just I have, I have in mind that I should move more often.

I: Ok.

P: I think it may, it'll probably help me uh, I'm pretty sure that's probably what's gonna motivate me, but um, for, I've always had jobs where you sit down, you know.

I: Uh huh.

P: Sedentary, so that kind of uh, creates a problem, you know.

I: Ok, so.

P: So, I need to, yeah. I think, I think my breathing may improve too.

I: Ok.

P: So, yeah.

I: Ok. Um, so the next question is: how important is exercise for patients with kidney disease, in your opinion?

P: Um, I really, I really hadn't given it much thought, you know, until it came up and I saw the thing about joining the study. I said "Well I'd like to understand that too.", because uh, I'm not sure, it'll probably um, it'll probably uh, help, in that uh, I don't know enough about the kidney functioning itself, you don't know how much it'll improve or whatever, by exercising. [Phone disconnects]

I: One second, the phone got disconnected, I will call back. [Busy tone] So at 12 minutes or 12 minutes 30 seconds uh, interview got disconnected. I will try to redo this, figure it out. I'm not gonna pause it, so as to keep it in one file, but I'm going to keep calling. [Phone rings and participant answers]

P: Hello?

I: Hey Ms. Name, sorry about that. Something happened there we got cut off, but...

P: Ok.

I: Um, we are back on track.

P: Ok.

I: Um, sorry, were you saying something, it just cut off, so I wasn't able to catch what you said.

P: Oh, I was saying that I uh, you asked me about how important was it to exercise for people with kidney disease and I said I really didn't know because I was...

I: Yes.

P: That's one reason I joined the study, to find out, you know.

I: Um hm.

P: I don't know enough about how the disease progress, what, what are the, will the exercise help it, or I don't know, wait and see.

I: Ok, so wait and see approach, ok. Um, and so, when um, what was it, sorry. When uh, you go to your doctor, does your doctor talk to you about exercise um, or give you exercise advice?

P: No, not really.

I: No.

P: Nuh uh.

I: Do they mention anything about physical activity?

P: Well, you know, honestly, I think he asked me about it, but um, no direct instructions. Like I said, I saw the flyer in his office and that's how I got involved in this.

[00:15:03.6]

I: Um hm.

P: So, uh, oh, I don't know if it's, I was curious to find out if it's gonna help, so that's why I joined the study really.

I: Ok.

P: Uh huh.

I: Got it. Um, ok so, what are some things that make it hard for you to exercise? I know earlier you were talking about you sometimes have arthritis in your foot and it flares up. Are there any other kind of barriers that you deal with like um, time, or um, anything else?

P: No, I just need to take the time, I've got plenty of time.

I: Ok.

P: I just need to be doing more, I just need to do my active, you know, than I am now.

I: Ok. And so, would you say um, that you were mentioning your health barriers like previously, like with your diabetes and your heart um, heart stuff, but is that currently a barrier still? Or is it just, it's just the...

P: Oh no, it's actually, no I don't think it's a barrier still.

I: Ok.

P: I'm, I saw my cardiologist a year ago, make an appointment, she told me to come back in a year. So, I have no, I don't have chest pains or any kind of problem with that, yeah.

I: Ok, alright. Um, what would make it easier for you to overcome um, your arthritis um, or that one health barrier you were talking about, you have anything in mind?

P: Um, no uh, I, you know, I think I, I look forward to having superficial advice on that because yeah.

I: Ok. Alright so, now the next couple questions are gonna talk about um, what your ideal program would look like. And so, one of the things that we are considering in our exercise program is group classes. How do you feel about group classes in general?

P: I, I think I would like that. I would like group classes like, I think it would motivate me.

I: Ok.

P: Yeah.

I: Anything else you like, you would like about group classes besides motivation?

P: Uh, just being around other people, I think. Uh, yeah, that helps. Uh, people who uh, all having the same problem or, you know, can encourage each other, support, that kinda thing.

I: So, what are some things that you would dislike about group classes, if any?

P: Uh, let's see, none that I can think of at the moment.

I: Ok. Um, we can come back to that in a bit. Um, and so, um, are there any barriers to you, or for you in terms of like transportation? Is that a barrier that you deal with, in terms of like finding, if we were to host these sessions in um, a specific area, is it harder for you to get to places or do you have a mode of transportation?

P: Well I take public transportation, so I don't, I don't have a car, I don't drive.

I: Ok.

P: So, I take public transportation.

I: Ok.

P: And uh, that was the other thing that I was told, it's gonna be at Emory Midtown. And where I live, the bus stops where I live. Comes to [inaudible] avenue and Peachtree, right. Yeah, and otherwise I'll take if I, sometimes I take Lyft.

I: Sometimes you take Lyft, ok.

P: Uh huh.

I: So, there's ways around transportation.

P: Right, uh huh.

I: Ok. Um, and so, we're also trying to gain input on how we should design these classes to be most convenient and appealing to you. And so, what kind of activities would you like to do? Um, I know you said you walked before, would you want to continue to walk or would you wanna try different things?

P: I would, I would try different things, you know [inaudible] just cause I don't know anything about, yeah, and then the trainer, I guess. We can try, I'm willing to try something different.

[00:19:59.1]

I: Ok.

P: Yeah.

I: But if um, but in your mind like you wouldn't mind just like walking, doing that kind of exercise, cardio?

P: Oh yeah, that would be fine, uh huh.

I: Ok. And so, we kinda touched upon this too, but just to reiterate, for time, you said the best time for you would be mornings, right?

P: Right.

I: And um, would you say like early morning, like closer to afternoon?

P: You know, maybe somewhere in the middle.

I: In the middle so like, like a 9am or a 10am?

P: Um, maybe 10am, but whatever time, I'm willing to you know, uh, come, whatever time.

I: Ok.

P: That's gonna be [hell?], I'm not gonna, I'm not gonna be a stickler about that, but...

I: Ok.

P: Uh huh.

I: Um, and so, what act or, what would you value most from an exercise program like this?

P: Would I value most? I'm guessing uh, that I would definitely, probably feel better.

I: Um hm.

P: Uh, uh physically and mentally I guess, I would feel better having you know, coming to an exercise class and knowing that I'm doing something for myself, yeah, to benefit myself so, uh, I'm kinda thinking that might be...

I: What you would value the most?

P: Uh huh.

I: Ok. Um, how would you judge if the exercise program was successful? And, for you, so first like individually, but also like program-wide.

P: How would I judge? Um, hm, I guess, considering the things that I said like I might be able to sleep better, or even feel better uh, and any kind of change, any kind of good change, you know, in my life because of it, I think that would be it.

I: Ok. So, any kind of positive change, that's how you would judge if it was successful. Do you have any specifics on like what good change would mean for you?

P: Uh, like I said my uh, a good change would be uh, probably more, I might even, you know, I might even have more stamina. I might be able to uh, and truly like I said, maybe get, maybe able to sleep better.

I: Ok, yes.

P: Yeah.

I: Alright, and so what about like a program success, how would you judge if the, the entire program was successful for everyone?

P: Uh, uh let's see, I don't know uh...

I: Ok.

P: Yeah, maybe we'll...

I: Yeah, that's fine if you don't know um...

P: Yeah.

I: Ok, well I guess we'll see right?

P: Ok.

I: Um, and so we have two more questions. This question is asking you about: what is your experience with exercise instructors? So, back like when you did exercise more, did you have a group exercise instructor?

P: Yeah, we had a group exercise instructor here at the complex where I live.

I: Ok. And so, so what about the instructor did you like um, like what qualities of the instructor would you look for in somebody in a exercise program?

P: Well you know, first of all, anytime, if you get an instructor that's not judgmental, you know, cause I've been around people who think, with an instructor who is judgmental that, and you know, they kind of, they [inaudible] and they want everybody to make [inaudible]. If a instructor is um, really interested and paying attention to each of the individuals, because we're not all alike. I don't like the cookie-cutter thing, where everybody's just alike and you, and everybody should be able to do the same thing, so that's not gonna work for me. So um, uh, yeah.

I: So, you want, you would want an instructor that would help you individually, like, like work with your needs and your, your level of ability?

[00:25:05.9]

P: Oh yeah, or at least, or at least um, recognize, yeah, that we're, that we're gonna be different.

I: Ok.

P: And uh, yeah.

I: Um, ok, anything else that an exercise instructor could do to help you be more willing and excited about exercising, or the...

P: If um, yeah, if I had an instructor who help, if there's certain, if you have questions,[and they keep don't understand?] and you really need somebody who can uh, explain things to you and understand, you know, that uh, and be very patient, you know. And be patient, cause you get to be my age, you need people with patience. Uh, like I said, understand that I'm not gonna, I'm not gonna have the same energy or the same uh, uh, let's see what I wanna call it, um, I'm not gonna have the same energy or the same uh, let's see what I'm trying to say...

I: Ok. Take your time.

P: Uh, yeah, that I'm not gonna have, that I'm not gonna have the same energy and probably not the uh, what's the term I'm looking for? Um...

I: Like attitude? Um...

P: Yeah, that'll work, that somebody younger than me.

I: Oh, I see, I see.

P: Yeah, it makes, it makes a difference, you know. So uh, as long as they understand and realize that we're in a group and everybody in the group is not gonna be working at the same pace and not have the same uh, energy level [as they did before?].

I: Ok.

P: Uh huh.

I: Ok. Um, so, how helpful is goalsetting for you when exercising? So, I know you...

P: Huh?

I: ...said you wanted to sleep, sleep better, but if you were to like think of a goal, like a, a goal right now, like with numbers, with like measurements, like would that be helpful for you when you're exercising?

P: Uh, I'm pretty sure, I'm pretty sure I'll be, I should have a goal, to have goals in mind, cause something to work towards, you know.

I: Yes.

P: If I'm just exercising for the sake of exercising, look and see whether the results or whatever, go like that.

I: Ok. That's great. Um, and so we're on our last question. Thank you so much for everything so far. And so, as you know we are already using um, a mobile phone app as part of our program, the Garmin...

P: Uh huh, yeah, I'm having fun with that.

I: You are? You enjoy it?

P: [Laughs] Well I had, ok first of all, I had to get a new phone. I really did because the, my old phone didn't have enough storage and all that kinda stuff on it, these apps, right?

I: Um hm.

P: And then I had to figure out, so I go on the Garmin website and you know, to figure out what I'm doing and sometimes I forget because I have to be near the phone to sync the, to sync the device right?

I: Uh huh.

P: Ok, so sometimes I miss the mark, sometimes I don't get it so, I think I've got that worked out. I've started to sync it every morning and so Garmin says, "Don't sync it too often, it'll wear out the battery." I said, "Well you know, I didn't, I'm told to sync it often." So, I'm not quite sure about that part, but I'm getting there. I, now I understand how it works.

I: Ok.

P: Yeah.

I: So, are you having any difficulties um, everything seems to be working fine?

P: Right, I'm, yeah, I think I finally got the hang of this.

I: Ok. Perfect, that's really good news.

P: Uh huh.

I: Um, so yeah that was the last question. Um, is there anything else that you wanted to talk with me about or share with us about your experiences or any clarifications you need?

P: No, not right now, I'm doing pretty good, I think.

I: Ok. That's great to hear. Um, thank you so much again for your time.

P: Oh, you're welcome.

I: Give me one sec, I'm gonna stop recording right now.

P: Ok.

[00:30:06.7]

**Audio File Name: E-08 1-03-2019.mp4**

**Recording Date: 1/3/19**

**Interviewer: AB**

**Transcriber: BB**

**Dates: 3/23, 3/26 2019**

**File Name: #8 CKD Interview Emory University (1-03-2019)**

[00:00:00]

I: Alright, so, this is interview 8 of the Exercise is Medicine CKD study. Um, so Ms. Name?

P: Um hm, yes?

I: Could you tell me about a time you were physically active that you really enjoyed?

P: Um, I really enjoyed, was uh, 2014.

I: 2014, ok.

P: Um hm, 2014.

I: And what about, what did you do in 2014?

P: In 2014, I, I walked, I uh, started walking at 6 o'clock in the morning and I walked for an hour. And then I come home, and I fix my breakfast which consist of oatmeal and some apple cut up in it and uh, then I would go to the Y. I had, I would have a 9 o'clock uh, class at the Y, which I would do spinning.

I: Spinning class, ok.

P: Spinning, yes. Then after that, I would go to the pool, and be in the pool for an hour. And then after that, I would have a 45-minute stretch that I did, um, because I was always catching cramps. So, I, they wanted me to do spinning after class, but I couldn't spin after class cause they didn't have no class, the next class would be 1 o'clock. And then I start um, I went downstairs to the fitness room and start working on my um, my chest and my arms and my leg and my core.

I: Um hm.

P: I did that um, cause I went to the Y every day.

I: Every day, ok.

P: Every day, I went Monday through Friday.

I: Ok.

P: But I didn't, I didn't work on my, my um, muscles and stuff, but three times a week. Cause they told me I need to rest in between.

I: Um hm.

P: Let the body rest cause I would get sore, and uh, let it rest but I could continue on doing my water, so I got in the water every day.

I: Ok, so...

P: And then, I...

I: So, you really, so it sounds like you did a lot of activity like, like you did a lot of different types of activity, all in the same day. And then you said you were working out every day, is that correct?

P: I would work out every day, I would get in the pool every day.

I: Ok. And so...

P: Every day and, but I spin, I spin three days a week. I worked on machines three days a week. I spinned three days a week, and I was in the water every day.

I: Ok, and you really enjoyed this, you had fun?

P: I really enjoyed it cause once I saw the weight coming off and people was giving me compliments and, and I just felt good. I just felt good but what happened to me, is my feet. My feet started burning.

I: Oh ok.

P: I could uh, start walking and they would just, within ten minutes both my feets would be burning. It started out with one, then I bought some Dr. [Names]’s, which wasn't nothing but a waste of money. And uh, I don't care what kind of sneaker I bought, it didn't help, they would burn. And now that I finally get back and walking now uh, I don't walk as far because of the rain, and I'm not trying to catch no cold or nothing. Um, but I try to walk in the house and going in every room in my house.

I: Ok.

P: It's working. And then, and my feet start burning when I use the elliptical now, so I slow it down, and that's it.

I: Ok. Um, so that's a great answer. Um, for the next question I'm gonna ask you about um, what do you think when you hear the word exercise?

P: I, I think for me, is, is motivation for me, in a way because like I said, I'm 67 years old. I, you begin to lose your muscle mass, your bones begin to bother you, and I think the more that I do, the better I'll be. I'll be, you know to be, keep all this stuff moving.

I: Um hm.

[00:05:00.6]

P: Because if I, I found this out too, if you don't keep it up, you lose it. You lose it, and I'm trying to um, I'm trying to live. I'm trying to live. I'm trying to get myself back, my mind back, uh, because I, I lost my husband. And it's, it really set me back. And I'm not like, I'm one of those people that when they get upset and things go wrong uh, I eat.

I: Ok.

P: I go from, I go from vegetables, mean I have nice vegetables, a nice salad, and I'm on sweets. And I eat my sweets first.

I: Um hm.

P: You know, and then I, I eat the vegetables, but half the time it's the sweets and I don't really want the vegetables. And I'm trying to, there's stuff that I never had, never tasted before. Like um, broccoli, cauliflower, I just start eating spinach.

I: Um, wow.

P: But, but a cauliflower and asparagus um, what else I just, I don't eat uh, um, I just started eating beets, um, some other stuff that I don't eat.

I: Um hm. So, you, so you've been, so you've been eating healthier lately...

P: ...and them little balls, that they say cabbage or brussel sprout uh, I never had uh, hm they're always talking about it, is green, avocados, it's called avocado?

I: Yeah.

P: Yeah, I never had that. I tried the um, mango. I never had a um, it's like a fruit uh, it's big, a mango.

I: Ok, got it. So, when you think of the word exercise, you just think of being more healthy, is that correct?

P: Just being healthy, just being healthy, you know.

I: Ok. And so, um, currently are you exercising?

P: I am exercising in the bed.

I: In the bed. So, can...

P: I try, you know, those, when I first talked to you and you was telling me about the exercise, and I did the exercise with the guy with his hand in the back and raising the other end. Oh, I did four of them and twice and my back hurt, I had to stop that, stop that, I stopped that. And...

I: So, could you tell me about what you currently do? So...

P: I currently um, lift my legs up in the bed. Then I put them, I put my feets together and lift them that way. I turn on my side and, and like uh, grab my legs up and now one at a time, I gotta bend and I'm just bringing it up. Trying to get some of the uh, meat I got in between, you know. And then I turn over and do the opposite side and I'll um, try to lift up in the bed by, by turning my body, my stomach to the, to the bed and lift my legs up. I work on my arms, I bend them and, back and forth with my arm, and on top of, on the side of my head, just bringing my arm in and out.

I: Um hm. So, you do...

P: I be trying to hit the whole body, but I don't.

I: Ok.

P: Um, I'm trying to learn how to breathe in and out of my nose cause I can hold my stomach in, but I breathe through my mouth instead of doing the opposite way.

I: Um hm, ok. And so, do you also do like, some sort of cardio, like walking or biking?

P: I was, like I said, I walk through the house.

I: You walk through the house.

P: Just walking, yeah, I just walk through the house right now. You know, because it's been raining, and I was um, walking from one hill to another hill, but since the rain got harder, I just do what I can in the bed. And then, I'm a changing, changing a closet, the winter clothes go to the back and the summer clothes come to the front. So, I'm doing some of that. I'm cleaning out my garage, cause it's running that over, and um, I tried to get myself a um, to give to the churches uh, for the needy. I'm trying to do some of that, so they can give some winter clothes too uh, yeah.

[00:10:17.6]

I: So, you walk around the house, you walk around when you go to church, um and then you also do...

P: Huh?

I: So, you walk around at the house and you walk around when you go to church and help out there. And then, and you do a lot of just like, body stretching and like lifting in bed.

P: Oh, I have to stretch this body cause it cramps up, my whole body cramps up. And then they was giving me um, uh, the medicine but it's not doing me any good. Just like they was, they give me um, oh I can't even think of it, but it's for fibromyalgia and all it did was swell me up. So, I couldn't, couldn't take that no more. So, but...

I: So then, so earlier you were talking about how in 2014 you were very, very active, and so, um, did your exercise routine change when you were diagnosed with kidney disease, or was it more, was it a different reason?

P: It was a different reason. Uh, I didn't even know I had kidney disease until Dr. um, oh I can't even think her name, [Name?], [Name?], she recommend me to Dr. uh, to Dr. [Name]. And I didn't know what kinda doctor he was until he told me. And then, every time I would go my cranium, is it cranium, um, it was higher, and nobody tells me what do I supposed to do to keep it down. And I called him cause he, he had sent me this paper and telling me how my, you know, I'm at 39 percent and now how can I stop this? So, uh, I notice that, well in my husband's family, they got it, they got it bad. So, one of them lost weight, and when he lost weight, he came, he was on dialysis, he came off dialysis. He don't take no kinda pill, he just watches what he weight, watches what he eats and walk. So, I'm saying if he came off diabetes, it cause, I've never known nobody to come off diabetes, I mean come off um, what you call that, that machine that they have to go through?

I: The blood sugar one?

P: Nuh uh, the um, horn that they um, take your blood, or take your something out of you, dialysis.

I: Oh yeah, dialysis, yes.

P: Yeah, I've never known anyone to come off of dialysis. Now, I had a niece that was uh, a diabetic. Well she uh, had um, she started with um, juvenile diabetes. And then, they had found her a kidney, but I don't know if anybody was really paying attention to her heart. And when we took her down there, uh, she told em, she asked the surgeon how long he's, he's been doing it. And she, he asked, he told her, he said "You'll be about my tenth one." And she told him, I know I'm getting off the subject, but she told him that he wasn't [inaudible] to do the surgery. And he said, "Why?", she said "Cause you’re not." She died.

I: Oh no, I'm sorry.

P: She got a nerve hitten wrong and all her body started falling.

I: Oh, well let's hope that's, that's not gonna happen to you or anything.

P: No, I felt bad that [inaudible] I pray for that though. It [remind] you know? It makes me stay motivated, cause you know, I don't know if they told you, but I had a hard time filling those papers out. And um, uh, and then my blood pressure was up so high, so I been praying about all of that and getting me motivated for this, I'm ready for this.

I: That's perfect. That's what, that's what we want to hear. And so...

P: I'm ready for this and as soon as it start, the better it is gonna be for me.

I: Yeah, it'll start soon, it'll start soon. Um, so now we're gonna move on to our next question, which is kinda what we talked about earlier, but could you name me like, some benefits of exercise in your opinion? Just like real, real quick, just like um, what are some benefits of exercising in your opinion?

[00:15:12.0]

P: It's, it's for your bones, it's for your muscle, it's for your heart, you know, it's for your heart. It's for you to be active, the, the more you're active, the better you are. You know, cause as you get older, things creep in, you never, you never realize it you know, but things will creep in. Your knees get bad, your back get bad, you know. Also, just, just, just to stay healthy. Be motivated, you know.

I: And so, would you say that um, you're motivated to be healthy and that's why you want to be physically active?

P: I guess, uh, I wanna be, I want to be.

I: Are there any other motivations for you to be...

P: Are there what?

I: Are there any other things that motivate you to be physically active?

P: Yeah, my grandbabies. [Laughs] Yeah, my grandbabies, they make me wanna be more active. You know, cause they like to go to the park and stuff and, and um, I wanna be active with them. I wanna be able to hold they hands cause I can't pick them up now, now hold they hands and, and say we're gonna do this, and we read a lot and um, we spelling out numbers now. We getting on numbers and stuff, but yeah.

I: Ok. So, grandkids, ok. And so, the next question is um, how important is exercise for patients with kidney disease? Um, do you know...

P: Well, no I don't, but I um, consider myself having a uh, cause I don't know what, what you don't supposed to have, what causes um, kidney disease. What you don't supposed to do, what you do supposed to do, nobody ever, even that guy, never broke it down, you know. So what I try to do, what I do, I drink grapefruit juice, I'm off of salt, I'm uh, off of uh, fatty foods and I try now, now, this, these last two weeks I have not been, I'm, these last two weeks I was not um, right. I, you know, because of the holidays and I'm going different places and stuff so I, I wasn't working out, I wasn't thinking, I was thinking about eating. And I know I have put on some weight since I first had my uh, when they first gave me my, my uh, thing and stuff. And, well right now, I wanna work on um, my kidneys, and I wanna know do blood pressure have anything to do with it because um, my blood pressure was up. And then, I'm getting these headaches, so I'm, I'm putting all this stuff, I don't know if it's stuff with kidneys or not. I really don't know what kidneys is all about. I know kidneys, it comes from the blood pressure, I do know, I do know that, that, but I don't know, it say like once they change my blood pressure, it made my heart rate go down. So, Dr., Dr. um, Name changed that, but I don't know how it is now.

I: Ok. And did your doctor talk to you about exercise specifically?

P: No.

I: No, they didn't give you any advice or anything on how to be more active?

P: No, not that I can remember.

I: Ok. Alright. And so, I know we touched upon this earlier um, with your feet, but what are some things that make it hard for you to exercise? And this could be like physical, it could be time, it could be like, uh, motivation, for you, so like for you yeah, what makes it hard for you to exercise?

[00:19:47.6]

P: Make it hard for me to exercise is my knees, my feet, and, and, and to get upset. I would put it off, and put it off, and put it off, and next thing you know it's another day. But, if I'm, if I'm motivated, I'll go outside and walk. It would be a little bit earlier than this, it's before dark, and I got my uh, golf stick, golf club and I'm ready, go and scare the animals, and, and I'm ready to walk.

I: Ok.

P: Uh, I, I'm not, I don't think I'm hard to get motivated once I get, get started about two or three days, I'm, I'm on the go. Cause I would walk on seven days, seven days I would walk. I would get up at four, I had to go to church, get out, walk, come home, take a bath, get ready to go to church, go to church, come home and then, for what, I mostly eat out, sometimes by myself. So, I go to Piccadilly’s somewhere that I can get some vegetables, and uh, come home. Now my goal this time is to work out in the water twice, twice a day. That's what my goal is today.

I: Ok.

P: I get it off during the morning time, what I done ate uh, I'm eating salads at lunch and I eat um, probably uh, in the evening time, just the vegetables. Just getting that vegetable plate and divide it for two days, eat the same thing for two days.

I: Ok.

P: And somebody would, if potassium, is potassium, what is potassium? I know this wasn't your question, but I wanna know because somebody asked me was I doing this thing for my potassium.

I: Um, so uh, you know potassium’s in bananas, right?

P: You are right, but I don't like them.

I: You don't like uh, bananas?

P: I don't like bananas. I grew up with bananas. That was my daddy's snack at night. You gotta, and there was eight of us, and he made everybody eat a banana, ugh. [Laughter from both]

I: Yeah, so I don't know if you wanna know about like, the specific chemical things, but it is very, it's like a very important thing um, important chemical in your body. I would, I would ask your doctor for more like, specific information, but um, there is a reason why they always ask you.

P: Ok, ok.

I: Um...

P: And mostly alright, I just, I just started taking them, and then she told me today that that wasn't enough. That I'm, I take the pills, but she says it's just not enough, you need over a thousand. And I'm eating a hundred, and I'm thinking I'm, I'm thinking I'm doing something.

I: I would ask um, your doctor how like she would, like if she had any recommendations on ways you can get more potassium, maybe it's, maybe it is you have to eat it. So um, but um, just to get back to the question um, so your knees make it hard for you to exercise um, but, and you sometimes feel unmotivated, but when you do finally get started, you’re ready.

P: I'm ready, I, I, I, I fight the pain. I will fight the pain to get, cause I'm ready to go.

I: Um hm, and so what would make it, sorry what were you gonna say?

P: I would say uh, that's why I always carry my swimming clothes. I always have a bag in my trunk of my car. So, whenever it hit me, I'll go. Like I'll go to the grocery store, Walmart, Sam's, and just walk up and down the aisle, just walk. Cause I can lean, or when the knee get to bothering, I can lean on the uh, the uh, what is it, what is, the buggy uh, I can lean on that. But to get my speed up, I can get my speed up and then when I get my speed up, that's when my knees wanna hurt. So, I have to walk and get me a buggy and then get back to getting it. And then I have left the buggy and just started back walking. If it's uh, if I slow down, the pain, the ease of, of slow down, and then I can pick it back up again. And I go up and down every aisle, every corner in the store, I go up and down.

[00:25:05.1]

I: Ok.

P: Yeah, I do that.

I: Ok.

P: Cause I like, I like it, I like when I lose weight, I like it. I have been struggling for a while, what I, well yeah for a while, I have been struggling, I get it down and like I say uh, something disaster, I have on or something, and then I just go to eat.

I: Ok. So eating is your coping mechanism kinda?

P: Yes, yes. Cause I don't smoke cigarettes, I don't drink that much you know, so I don't have nothing else to cope it, but eating.

I: Ok.

P: And then my thing is too, when I get through eating, I go to sleep.

I: Hm, ok. So, so what do you think would make it easier for you to overcome this barrier of...

P: Well I think, to me, is getting my eating in control, where I don't eat at night. I have to have my meals um, what I usually do, I have to have my meals no later than 5 o'clock. If I feel hungry, I've gotta have something, I try to have some fruit around the house, grapes, apples um, now the halos are out uh, uh clementine out, I mean I have that in the house, I have that in the house. I, sometimes I go overboard with that, instead of having one tangerine you know, they so small, I may have two. But my thing is candy. Candy, a bag of popcorn, um, and I've kinda slacked off on the ice cream now, ice cream don't taste that good to me now. Uh, uh, but that's my biggest problem.

I: Ok. So...

P: Once I get something that I like, I go overboard with it.

I: Ok. So...

P: Cause I would buy popcorn, they knew when I came to the store what I was coming to the store for, and that's bad, that's bad.

I: So, do you think if you um, do you think that if you were able to um, address your eating behavior, it would make it easier for you to exercise?

P: I know it would!

I: Ok.

P: Oh, yes! Yes.

I: Ok. So, that's another motivator, ok.

P: Yeah, I [inaudible], that motivates me, because I said I have to eat at a certain time. I can work at, I can go back and work at, I can walk up and down the street you know, in the neighborhood. Just walk cause it motivated me, to do something, not to you know, not to just lay here and watch the TV.

I: Um hm.

P: You know, and sometimes when I can't sleep, I get up and, and wash a load of clothes, dry them clothes, put them up, you know. I can't, when my son was over here a while ago and he said, "I'll be over here this weekend to mop your floors." You know, I can't, I can't do any of that because of my knees, my knees. And my dad never did believe in women's mopping, you know, he didn't believe in it us mopping, us girls mopping. And then, my husband, he did it too, he didn't, well your daddy done spoil you so I can't stop it.

I: Um, that's funny. Ok, Ms. Name, um, so thank you for that. We're gonna move on to more specific questions about the actual exercise program. And so, one of the things that we are considering is a group class. And so, how do you feel about group classes in general. So, like what do you like about them and what do you dislike about them?

P: Well, I like group exercise, the point I don't like is what I'm, I'm doing. Uh, if I don't know how to do it, everybody in the class is on the same leg and I'm on the wrong leg. They uh, jump and I, I'm not jumping when they jump. They're so important, I don't like about exercise, but if I know what I'm doing, and I'm keeping up with the class, I'm fine. I am fine. I love cause that, even those people that you're around, they motivate you, cause you looking at them, oh she losing that weight, she losing that weight, you know. He looking good, and then we started discussing how much weight I lost, oh, that made me come on now, I gotta catch up, I ain't losing that much, and I know it's gonna start off slow.

[00:30:09.2]

I: Um hm.

P: I know it's gonna start off slow, but I know I can pick it up. I had, I can pick it up and cause people doesn't believe that I'm 67 years old. And I said yes, I said my birthday is in October, I just had my 67th birthday, and I'm trying to live. That's my goal, is to live. In order to live you've got to move, you gotta do something. It's not gone, it's not gonna, it wasn't, it was easy to put on, but it's hard to get off. So, I know I have to work in order to get it off. And I have to get my taste buds changed and everything to do this, I'm ready for this. God is ready for me to be like this, he don't want me like this, he want me lean and fine, this is my temple. Ok what's the next one.

I: Um...

P: I'm just telling you how I feel, this is how I feel about the situation.

I: I appreciate it and I'm glad you're ready cause um...

P: Oh, I'm ready, I can't wait. It's the 12th right?

I: I think so.

P: Ok. You're not sure either, well I need to, I need somebody to make sure, I'm on, I'm on time.

I: Yeah, we will, we will, we'll do that. Everyone kinda, coming back from vacation now so.

P: Yeah, yeah.

I: But just uh, look out for some emails. Um, and so, the next question is um, you kinda touched upon it, but what kinds of activities or exercise activities would you want to do um, like...

P: Well uh, I, you know, I, I wanna do spinning, I definitely, and...

I: Spinning, ok.

P: Do ya'll do line, I mean some line dance?

I: Line dance? Um, I know what it is, I know what it is, I don't do it but...

P: What you mean you don't do it?

I: I don't...

P: Well you gonna have to get in there, you have to get in there!

I: You'll have to show me.

P: Huh?

I: You'll have to show me.

P: Ok, ok well just gotta go on YouTube and it'll tell you everything.

I: So, spinning, line dancing, what about...

P: Um hm, I uh...

I: Other activities.

P: Huh?

I: What about any other activities? What else would you be interested in?

P: Well, I was doing yoga.

I: Yoga?

P: Yeah, I was, and the doctor told me to stop, cause I had messed up my, just as I had learned, the jackknife, and that's when you put your hand and you got your legs up in the air, and you, just as I learned it, I messed my back up.

I: Oh no.

P: So, he told me, he told me not to do that. And I, I was losing weight off of, off of yoga, too. I had, I had that fast [tour?] but I didn't do but 30 minutes of that class. And another class I really wanna learn, but I'm too heavy for it now, is the barre. You ever heard of the barre?

I: The barre?

P: Uh huh.

I: I think I have, yeah.

P: Ok, yeah, that's another one that, that I really, cause it's got a lot of stretching in it.

I: Ok, and so would you like to stretch a lot, is that one thing you wanna, wanna prioritize?

P: Yeah, cause like I said, I have a lot of cramps. My toes, the bottom of my, my foot, my calf on my leg, my whole body cramps, my hands, but I'm ready.

I: You ready.

P: I'm ready.

I: Ok.

P: I'm ready.

I: And so, in the exercise program, let's say you were, you're on it, what would you value most from it?

P: The most I'd value from it is learning, learning how to eat right and to do my, do my exercise.

I: Ok.

P: That's, that's what, that's what I'm, I wanna learn, that's what I wanna be, I'll stay motivated for uh, I like trying new things, whatever. Uh, whoever must try this, I'm gonna try it too.

I: Ok.

P: You know. I'm gonna try it too.

I: Perfect.

P: And like I said, I think other people can motivate you too, not just your teacher, your instructor, just looking at the other people do it, you know.

I: Ok.

P: And picking up their speed and stuff like that.

[00:34:59.9]

I: Um hm. How would, how would you judge if the program was successful?

I: I just tell it how it is. Everybody should try it. You know, you never know what you can do if you don't try.

I: Um hm, and so...

P: And I say I'm gonna try, I want everybody gone know, gone be successful, so if I want to be the one being showed off. [Laughter from both] Ya'll gone be bragging on Ms. Name.

I: Oh, I'm sure.

P: [Laughter] Ya'll gone, she came in and had a little slow, but look, look at her now.

I: Yeah, that's good. So in the, you were also talking about earlier how you would want to lose weight um, and you also want to be able to like, have less cramping in your legs, so would you have any other goals for like, the program, like any other goals you would reach for?

P: Yes, I would reach to continue on exercising, in the class, in the class, because, ok at the Y, they don't, they don't motivate me. I do a lot of stuff by myself, except for my spinning and my uh, uh, oh now I can't think of the word, elliptical. Because you get on them yourself and you time yourself and all that, but see I need motivation first. And, and when I'm cycling the teacher get off and she checking how much, how fast you going and stuff like that, and she be, she be noticing how fast I um, how picked up my speed. Now I done lost it again uh, but I picked it up real fast and had no problem with my knees or nothing, but I think, to me, weight messes with your knees. I think concrete messes with your feet, because you're on it all day. If you up, you're on it all day, no matter where you go, you on concrete, even if it's your house, cause your foundation is concrete. You got carpet but you got concrete under there. Cause you got plush carpet, you know, listen, but I don't have no plush carpet, but um, I, I, I actually believe those two things would help me.

I: Ok. And so, I know you also mentioned that when you were at the Y, you would often just work out by yourself and you weren't...

P: Yeah.

I: So, we are gonna be using exercise instructors for this program and so, what could the exercise instructor do to make you feel more motivated to hang out, or to work out and excited about exercising?

P: Ok, music.

I: Music? Ok.

P: You got to have music. I mean you can have, you can have country music, and you can have pop, you can have it, but long as it's time to slow down, that's when the slow music gotta come on. But when you, when you working after you done warmed up and got yourself started, you ready for that gas music. You ready for it, cause it's gone keep you up, it's gone keep that heart rate up until it's time to slow it down, cause you gotta slow everything down, you gotta stretch and everything. And that's what I want my instructors to be able to do.

I: Ok.

P: You know, we get, at the Y, we, we up on the bike and then we sit back down, you up and down, up and down, you know, and then she slows it back down. She'll tell you, "Ok now gonna slow it down, we gone bring your heart rate down a little bit, and then we gone pump it back up." You know, so that's, that's what I'm looking for in this, this, what I would like for this class to do.

I: Ok.

P: You know, you know, and I'm sure the instructor knows all this, you know, know all this in order to get the people motivated and wanna come. I'm, I'm excited to get to her class, you know, excited for her to walk through that door and say "Good evening, ya'll ready? Good morning, ya'll ready? Let's get ready! Let's get this fat on off of us! She dropping, she might drop."

[00:40:02.3]

[Laughter from both]

P: Let my fat drop off of me.

I: That's awesome.

P: I'm tired of carrying it, I'm tired of carrying it, you know. Like I said my knees is getting bad, I won't be able to dance. Yeah, ok.

I: So, you, so definitely the instructor has to use music um, and they also have to be motivating and wanting, they would have to, they want...

P: I want her to, to be, to let me look like her. I don't wanna be her, I just want the body shape.

I: Ok.

P: I want the body shape, cause my BM done, what is it, body, what is it? BM...

I: I. BMI.

P: Yeah, BMI. I want it to be where it's supposed to be. I want my waistline to be where it's supposed to be. My core, I want everything, like what she done work for and got. And still working to hold on to what she got, you know. And I just wanna learn how to do exercise right and to eat right. It go hand in hand. You eating, I mean you working out, but you overeating. You over [crunching?] what you doing. What I'm doing rather, cause I'll be sweating off some, then when I get home uh, if I don't stop by something, I'm going to get some candy, get my energy back up, you know. So, I, I...

I: And so, what are some things that the exercise instructor could do that would make you discouraged to exercise, like what are some things you don't want them to do?

P: Well, if something that I didn't want to do, I would just stand there and do something else. Says that she's uh, um, what's that thing, when you go over it um, hm, I can't even think of it. But like you lift your legs up, what is that thing called, it's, it's uh...

I: Elliptical or...

P: Not the elliptical, this is, this is you working the body now uh, and you, you got a stool like, and you jumping over the stool, you, you counting your legs, one, two, three, then you cross over and do the left leg, and you cross over and do the right leg, you jump over. I can't think of it, but um, and if I can't get the hang of it, I'll just uh, shoot I'm looking at mine right now and I can't think of it uh, I just do another exercise. I just wouldn't stand there.

I: So, if an exercise is too difficult or...

P: Yeah, yeah if it's too difficult and I can't, I just do something else, until I catch on to what she doing.

I: Ok.

P: Til I catch on to what she doing. She, she may be going over it, and I can't go over it, so I just walk over, til I can jump over it. You know, I'm not just gone stand there, I'm definitely not just gone stand there, you know. I may have to stand there til I catch what she doing, but then, and I'm, I'm um, uh, marching. I'm doing something, until I catch on to what she doing.

I: Got it. Ok so, it sounds like you would want the exercise instructor to be like, not too difficult, exercises won't be too difficult um...

P: Well I know she gotta make it difficult to get our shred and stuff, it's just that I may not be able to do it the way she doing it at first. And then, it may be where I have to be one on one with her for a few days, you know. She has to get off, say that she has to get off the spinner and come to me. And she says this is what I want you to do. Or I may go, go to the, come to the class and just to talk to her so she can show me. You know, that's, that's what, that's what, and if she can't come and show me that day, then I'll find out what day she can show me.

I: Ok.

P: [Laughter] You know, I wanna, I wanna be able to talk to, you know, to every, every instructor that I have that I can't do that right now, but let me watch you for a while and let me do my thing, cause I will continue on peddling.

[00:45:00.7]

I: Um hm.

P: If I can't stand up when she says stand up, and we, and we uh, turn, turn your uh, thing up, just, yeah when you spinning, turn it up. And I might be able to turn mine up as far as they going. Like they go up to 10, and I'm still at 7, but it may be because of my knees, I don't know. But I, I go as high, when she say turn, I turn.

I: Got it. Ok. Ok, and so Ms. Name, we're on the last question now, um, this questions just gonna talk more about, it's more about the app. And so, um, what do you like about exercising apps and what don't you like?

P: App, oh long as I can understand it, on the apps, like I told um, I'm not good with the, with the um, uh, the laptop.

I: Ok.

P: I'm sorry, I'm not good with that uh, matter of fact my uh, modem has, when it, it's the modem, something done went out. And they gotta send, they sending one tomorrow. And uh, he was saying "Momma you gone be able to do it". Cause my laptop is out, and uh, they surprised me and got me a uh, tablet, they got me a tablet. But, it's still in the box, cause I don't know how to put it together.

[Laughter from both]

I: Sounds like you should call them.

P: But I could, huh?

I: Sounds like you gotta call them.

P: Yeah [Laughter], but I went to school for, for a little while, but it was just two [congregations?]. Cause every time I'd, I done had it, I didn't, I don't have it. You know, I don't have it.

I: What about on your phone though?

P: Huh?

I: Your phone? So, are you comfortable with it on your phone?

P: Sometimes I am and sometimes I'm not, cause this, this phone'll jump off, it'll cut off on you, it'll do everything on you, you know. So, sometimes I'm not and sometimes it gets me frustrated, you know. Cause I tried to go back to those um, those exercises to see, what's his name? The one that do the uh, exercise on the um, for you all?

I: Ahad?

P: Ahad, ok, yeah. I tried to get back to his classes, but now I know how to do that, I told my son, I said, "I know it's on here, I just can't find it," and then I found it.

I: I'm glad you found it.

P: Cause I, I tried to have him put it where they all was together, you know, all together. So that's what I be trying to get him to do, he did that. I got my modem coming so, I know how to put it together.

I: So as long as the app is easy to use, you would continue to use it?

P: Ok.

I: Ok.

P: To be motivated, I mean continue, that ya'll know I'm moving.

I: Yes, ok.

P: Um hm, ok, ok, that'll work.

I: Ok, alright Ms. Name, well that was the last question. Um, I really appreciate you taking your time out of your day.

P: Well I appreciate you calling me.

I: Yeah, of course, of course. Um, I'm gonna stop recording right now.

[00:48:36.8]

**Audio File Name: E-09 1-4-2019.m4a**

**Recording Date: 1/4/2019**

**Interviewer: AB**

**Transcriber: BB**

**Dates: 4/14/2019**

**File Name: #9 CKD Interview Emory University (1-4-2019)**

[00:00:00]

I: This is interview number 9 Exercise is Medicine CKD Study. So, our first question is: can you tell me about a time you were physically active that you really enjoyed?

P: Uh, actually I've been physically active a good, a good portion of my, my life. You know, there may be uh, times you know, like a few weeks or months in between the times that I was active, but um, pretty much of my, especially my adult life, I tried to be active. Because I realize that exercise is very important, I don't care what age you are, it's very important and we, you know, we're meant to be active, you know, and not uh, you know, and not active. And so, uh, right now uh, I, I walk uh, the first thing in the morning and I get up about six and start walking about six-thirty. I have a, a friend, a neighbor of mine that we, during the summers, we actually walk outside together in the morning and, and uh, since it's cold now, we are able to walk inside the building we live in, you know, senior citizens building, and we are able, it's long and big enough that we can actually walk, you know, for an hour in here. So, I do that, you know, and that's how, how I get a lot of my walking in. I'm also a member of um, of Planet Fitness. I haven't been going there the way, you know, that I had planned on, but I planned on starting this year, you know, getting in there. Plus, I, I'm a member of a senior citizens uh, here in Atlanta, one of the senior citizens building and, and uh, I'm registered this semester, starting the 14th of January, to go to uh, the exercising. So, I'll be taking a aerobic exercise on Monday, Wednesday, Friday for an hour. You know, and I just, I really enjoy to, the answer to your question and I'm sorry I didn't answer right away, but to answer your question, I really enjoy walking every morning, you know, with my neighbor, you know, we talk and have fun, you know, out walking together every morning. So, I really, I really like that, you know, I depend on her and she depend on me, you know, and we, enjoy getting together, you know, and I, I enjoy doing it because I have somebody to do it with. Plus, I have somebody that I can be accountable to and she can be accountable to me. So that's the time that I truly enjoy walking, and like I said it's where I get my steps in a lot of days now.

I: Awesome, I'm glad you have a partner, an exercise partner that's always, that's always good. Um, but yeah, thank you for that. Um, the next question I have for you is: what do you think when you hear the word exercise?

P: I'm sorry would you repeat that question?

I: Um, the question is: what do you think when you hear the word exercise?

P: Oh, I think about uh, the actual, what comes to my mind is that, you know, exercise is very healthy for us. Exercise is something that we all should do, you know, be involved with, you know, what is, walking, or what is um, you know, doing other types of exercise, you know, um, like uh, you know, um, like some other different ways to exercise other than just walking. But um, when I think of exercise I, basically what I think about is that's something that we all should be doing and that's something that, that, you know, you should do, which is a part of helping keeping, keeping you healthy.

I: Um hm, yes, that's very important. Um, anything else that comes to mind when you hear the word exercise?

P: Exercise. Um, I just, when I think, when you say exercise, I think about the various exercises, you know, people can do it and like to do it, when I hear the word exercise. And I just, actually when I hear the word exercise, I just, I just think the word healthy comes to my mind. You know, something you can do to, to keep healthy, to keep yourself healthy. You know, along with eating properly.

I: Um hm, perfect, ok. So, our next couple questions are gonna talk more about your specific physical activity exercise routines. And so, um, I know earlier you were saying that you go on walks with one of your friends, um, and so, how often do you go on walks and do you know how long, like how, how...

[00:05:06.2]

P: Yes.

I: Ok.

P: We walk at least one hour, sometimes it's more, sometimes it's an hour and thirty minutes. It could be an hour and fifteen minutes, but uh, at least an hour. We don't stop before we know we've walked an hour, we used to walk from, from six-thirty to uh, to seven, seven-thirty, but at least an hour.

I: At least an hour.

P: You know, and, and we do it, we do it, you know, neither one of us have anything else to do. We generally do it from Monday through, through Saturday.

I: Wow, ok. So, you guys walk a lot together, pretty much every day?

P: Yeah we walk, we walk a lot and, and some, of course, some days um, you know, like, especially during this holiday season, I haven't been able to walk, you know, an hour every day, but I try to get in a certain amount of steps, but I don't always get in as many steps and I don't always meet my goal. You know, but most of the time I try to at least, most the time I think I reach my goal in a day’s time, but all of it may not be, you know, the actual walking that I do in the morning, cause I really consider that my exercise, but, you know, if I get um, 8,000 steps in, you know, from walking uh, with her every morning, you know, then the rest of the day I'm gonna get some steps in. So, those are the ones, you know, there just getting in because I am moving.

I: Um hm. And so, I know you were saying that you um, you have a pass to Planet Fitness and also a senior center, um, do you...

P: I'm sorry, actually, actually I'm having problems see like uh, I'm not hearing you. I, I hear you and I understand you but uh, it's just seeming not to be loud enough sometimes. I'm struggling to hear you.

I: Ok. Yeah, so you're on speaker um, but I'll, I'll try to speak louder ok?

P: Ok, I don't, uh, I actually have uh, a Bluetooth on.

I: Oh, ok. So, I'll, I'll speak a little louder, is this, can you hear this? My voice?

P: Yeah, I can hear you, ok actually what I'm gonna do is just take the Bluetooth off and see if it's better.

I: Ok.

P: Ok, go ahead and talk now.

I: Ok, so, can you hear me better?

P: Hold on just a minute.

I: Ok.

P: Ok, are you still there now?

I: Yes, can you hear me?

P: Ok, yeah, I hear you pretty good now.

I: Pretty good? Alright, just let me know again if it's not loud enough and I'll just raise my voice a little bit.

P: Ok, ok.

I: Um, so, the question I asked was um, I know you also have like, a pass to Planet Fitness, or you're gonna get one and one with the senior center. So, do you do other, any other exercises besides walking or is that the main thing you do?

P: That's the main thing I do right now. And uh, and um, the, the one that the senior center, I didn't get in the, the last semester, but uh, this, it starts again on the 14th of this month. So, I am in this semester, so I didn't walk last semester, but uh, I mean I didn't do my aerobics last semester, but I will be doing it this semester, starting the 14th of February. And uh, and I had not been going to Planet Fit, I would just go maybe once in a while, you know, so, we always make the new year resolution so, you know, if I go Monday and Wednesday there, I plan on going to Planet Fitness for uh, the um, you know, the weight, weight training exercise.

I: Ok.

P: Uh, and get the aerobics in, the senior citizen, then get the weight training at Planet Fitness.

I: Ok. And so, that's all in the future, you said the 14th?

P: Future.

I: Ok.

P: Yup, yes.

I: Ok. Um, so that's great that you have that planned. Um, so this question, so I, the next question I'm gonna ask you is um, how did your exercise routine change when you were diagnosed with kidney disease?

[00:09:38.2]

P: Ok. What I oh, it's so long ago since I was diagnosed with kidney disease, but it didn't, it didn't immediately, well, well actually I've been uh, my friend and I, we've been walking, we're going into the seventh year of walking, you know, every morning. So, I'm, I'm not sure how long ago it was, but uh, you know, when I first was diagnosed, I can't remember if I was walking at that time, but eventually I did. I don't know if my exercise immediately started changing after the diagnosis, but I do know eventually, I started walking. And I was probably walking because I used to walk by myself and then I saw this group that was walking every morning, so I said, here where I live, so I said well, I'm gonna start walking with them, but uh, eventually everybody broke off except my neighbor and I, that are still walking, we the only two that are still walking. But also, I would say that uh, probably I was already doing a little walking, you know, and uh, if, I've continued that walk.

I: So, being diagnosed with kidney disease didn't really change your routine much, would you say?

P: Uh, it did not, I will say it did not.

I: Ok.

P: Because that, you know, first, when I was first diagnosed, I didn't, didn't understand the true meaning of what was going on with my body and, and the doctor at that time told me that, you know, that, you know, that it sounded like I wasn't gonna get any better or uh, or this is something that was gonna be going on the rest of my life. So, it, so I didn't think there was anything I could do to change the results of the diagnosis, you know, so I, I probably didn't do anything, but I just, and when I started walking, I didn't think about, you know, it helping kidney disease or having any effect on kidney disease. I just knew that, you know, I was, needed to be doing some type of exercise, you know, regardless, you know, what I had kidney disease, but that was my purpose for walking and plus, I, my biggest motivation for walking and exercise for me is to lose a little weight.

I: Um hm, to lose a little weight, ok.

P: Uh huh, to lose weight.

I: Ok, and so, that's a good segway into our next question, which is um, what are some of the benefits of exercising in your opinion?

P: As I said previously, you know, for my health and, and to lose weight and to feel better, you know, get rid of stress, and so on.

I: Ok. And so, um, being more healthy and losing weight can be considered some motivations to be physically active. And so, are there any other motivators for you to be physically active, besides the ones that you've already mentioned?

P: Ok, no other that I can think of at the moment, you know, for me now.

I: Um hm, they could be like, health related or they could also be like a personal benefit to you.

P: Yeah, um hm.

I: Ok. And so, we can move on to the next question, which is um, in your opinion, how important is exercise for patients with kidney disease?

P: Uh, well, you know, um, one of the reasons that I decided to enter this program is because I thought that it, that I could get, I didn't know for sure, but I thought it was a possibility that doing exercise, you know, could, in some kinda way help my um, kidney disease um, but, you know, like I say, I don't really know for sure, but I thought um, eventually, you know, [inaudible] I could find out what really does help, but I do know that, you know, exercise is beneficial, you know, just for your health in general, yes.

I: Ok. And so, did your doctor ever talk to you about exercise um, or kidney, for your kidney disease management?

P: Uh, I don't think so, I don't think uh, I don't recall, you know, having any conversations concerning, you know, exercising.

I: Did they, did they tell you, you had to be like, physically active for a specific amount of time or?

P: Um, you know, before there, there's a positive effect on the kidney disease, is that, is that what you're asking? No, I don't remember, recall having that conversation either.

I: Ok. So, you haven't had any conversations with your physician about exercise at all?

P: That's correct.

I: Ok. Alright. Um, and so, the next question is gonna touch upon things that make it hard for you to exercise like, some barriers that you might um, have to deal with that um, make it less likely for you to exercise. So, could you talk a little bit about some things that make it hard for you to be physically active?

[00:15:06.9]

P: Right, ok. Ok, uh, I don't uh, you know, I may sound sick uh, and I have been uh, uh, you know, sick uh, down to the point where I couldn't even walk. Uh, and I don't, I don't have any ideas even now, what would that, what was the problem, the reason. Uh, um, it could've been uh, something like uh, uh, what is it that old people have, the therapies? Uh, when they, anyway um, uh, I don't really, now and in the past, but there have been points that I was sick for a few months and, and couldn't walk, but um, but uh, there's nothing really right now that uh, would cause me not to exercise. The only I have to say is that some mornings I really don't feel like getting up and exercising. I'm talking about, you know, I might not be feeling just too good, you know, if it, you know, it's not like sleepy or anything like that, it, it's just I don't feel, I just don't feel good, but I found out that if I get up and walk anyway, before I get through walking, before that I was up, I'm gonna feel better. And I don't, and generally, by 12 cause I'm, since I start walking about six-thirty, um, when I'm about 12, you know, I'm feeling, feeling much better than I did when I first got up. So, a lot of times, that's my motivation for getting all up and doing it anyway. I know I need to do it. And then I know that I'm gonna feel better, you know, eventually in doing that thing. And so, that's, that's the only thing that I feel, but there's nothing physical right now, that would cause me not to be able to exercise. And then some, now in the past I've had, you know, at some point in time, I've had gout and of course, you know, I can't even walk at that time. So, that causes me not to be able to exercise, but, you know, that happens, it's every once in a while.

I: Um hm, ok. So now there's pretty much no physical health barrier. Um, would you say time is a barrier, I know you, you are retired so you must have more time than most people, but is time still a barrier?

P: Time is not really a barrier for me, so since I'm retired it, then I choose to get up early and do it, if that's, that's why I get up early and do it because I can be consistent. If I get up early, you know, and early in the mornings to let, you know, let that be the first thing that I do in the mornings. You know, I can, I can do it because if I try to do it later on in the day, you know, I'm usually involved in other things, you know, and I'm not able to be consistent doing it.

I: Um hm, ok, perfect. Um, so the next couple questions are gonna talk more about the specific exercise program and what um, what you would like about it, what you would dislike, what kind of activities you would want to do. And so, um, one thing that we are considering, and you know this already, is that our exercise program would be a group-based class. And so, how do you feel about group exercise classes in general?

P: Ok, in general, uh, I, I like group exercises, more fun to be, to exercise with someone else than it is to do it by myself. And uh, so that's one of the reasons that I chose, you know, to go to the Monday, Wednesday, Friday group class, you know, at the senior citizen building, and uh, of course, you know, their exercise is conducive to, you know, people my age and uh, whereas when I go to Planet Fitness, you know, the, most of the people there is, you know, they are younger people. And uh, and that might be one of the reasons that I, you know, can't uh, maybe don't go as much as I should or whatever. You know, uh, pay for the plan there, what I'm saying is I, well my, actually my insurance pay sends uh, silver sneaker uh, they pay. So, uh, but anyway uh, group is, group is fine with me. But um, you know, it is, I like it better with uh, people in my age bracket because, you know, we all dial back and, you know, can do about the same thing and whereas the younger group of people, you know, it's, you know, they, they're gonna be much faster and much more energetic than I am, and they can do much more, you know, than I could, you know, can at my age.

[00:20:08.9]

I: Ok. And so, besides like, having like, like a disproportionate amount of young people in your exercise class versus people your age um, are there any other things that you dislike about group classes?

P: Uh, no I, I, I, well although the uh, senior citizen group exercise that I go to, there are a lot more women than there is men, but I think I feel a little bit more comfortable around men, than I do around women.

I: Around women, ok.

P: So, that might be another thing, you know, and not to say that I really, really hate it, but I just feel more comfortable than women, you know.

I: Ok. Yeah, no, that's fair. Um, and so, how do you feel about a, an exercise class created specifically for people with CKD?

P: Ok, I think that's very interesting because you got other people, you know, that all have the same thing that you have. So uh, I don't, I would be ok about that because we all have, have something in common. As a matter of fact, you know, when you have kidney disease, you can't look at a person and tell they got kidney disease, so when I, you know, uh, first had my first interview up there uh, at Emory and I saw a couple of other men, one of them was a older man and the other one was a younger man, but he was a big, strong-looking, tall man, you know, I was just surprised cause, you know, that he had kidney disease. So, I said to him, I said, "Well, you know, you can't always look at somebody and tell they have kidney disease." He said, "Yeah, man." All strong-looking and all that, but yes, I have kidney disease. You know, so it's just, to me it would be interesting, you know. And also, you know, find um, find out, you know, how other people are living with it and how they are getting along with kidney disease, cause I don't know of anybody else, personally, that has kidney disease, but I don't think a lot of people out there, but I don't know that they had it. And probably a lot of people don't know that I have it.

I: Hm, ok. So, it'd be interesting to have a class with people, where you have this in common with, CKD?

P: Right, right.

I: Ok.

P: I think it would be interesting that, that way, you know, you'd make them learn something from others, you know.

I: Yeah, no, definitely. Um, and so, one thing that I didn't touch upon in the earlier questions was um, is transportation a barrier for you, in terms of like getting to and from places or?

P: Transportation is not a barrier for me, you know, because I have a, I have a car. Um, but I was also thinking that I was kinda glad that I got in the group that, you know, was able to exercise, and didn't have to go up there uh, uh, because I, I can't uh, the reason I like the groups, the one that I'm in is because they, they're very close, very close. Actually, they all, except for the senior, it's about huh, it takes me about five, five, six, seven minutes to get there, but it's uh, it's very, you could walk it if you wanted to, but you may not want to. And the Planet Fitness is definitely within walking distance, you know, where I live so, I kinda like to go to places that are close by where I live.

I: Got it.

P: You know, Emory, Emory's not, like it's not very far away from me, but it's not uh, like really close either. And then, and I have, I used to go through downtown and there's a lot of traffic and I would hate dealing with that, going and coming. And that's, that's what I would have to deal with, the traffic.

I: Ok. Ok, so it's, it's not necessarily a barrier, but you do like places that are closer to you.

P: Right, I prefer the ones that are closer.

I: Ok. Alright, um, so the next question's gonna talk specifically about the kinds of activities you would like to do in these classes. And so, I know you said you walk a lot, and so, um, including walking, like what are some kinds of activities that you would like to do that'd be most convenient and appealing to you?

[00:24:51.1]

P: Um, ok about, um, pretty much most of my life I've done aerobics, you know, and um, and I have done yoga at one point or another, but it, but like I say, I'd pretty much be interested in losing weight and yoga is not gonna lose me a whole lot, you know, too much weight uh, if any. Um, trying to think of, and I have a uh, let's see I did the type where, you know, you do, do it at a fast pace and then you, and then you do it at a slower pace, and then you do it back up, I forget exactly what you call that, then you do it really fast again. That was, that was interesting, you know, especially when I first started doing it, I don't have the ability to do it now with my age. [Laughter from both] Um, I'm trying to think what else I have ever done. Um, I probably done one or two other things, but I can't recall right now, what else I've done, but now the uh, senior citizen place I go to, they have everything you could think of, they got it there. You know, and they do have the um, you know, the pool, helping people swim, there. And actually, where I live they, we have a health and fitness room and I used to go there all the time, but um, since I've been going to the other place, if I go there, but one of the reasons here they, where I live they, they had only a limited amount of machines, you know, very limited. Whereas the other places I go they, you know, have a lot of, especially at Planet Fitness, you know, they got a lot of machines and so on, so.

I: Ok. So, a wide range of things you would like to do um, and so, you kinda touched upon this earlier, but what would you value most from an exercise program?

P: Ok, I would value uh, having a personal plan for, for me.

I: Ok.

P: You know, and I would uh huh, a personal plan for me.

I: So, like a plan that you would work with, with um, like your instructor or something?

P: Uh, yeah, so, you know, sometimes they, they'll uh, you know uh, like Planet Fitness, they um, the trainer, the trainer would, you know, show you the exercises, you know, that are good for you or that, that will accomplish what you wanted to accomplish. And then they, then they leave you on your own to do those exercises every day, and if you, you know, have a problem or troubles, you know, they're there to, you know, help you with that.

I: Ok.

P: So, I think that's, that's helpful.

I: Ok. Um, we can definitely do that um, and so, I know you were saying that like, you would like to lose weight um, that's a goal. And so, in addition to that, how would you judge if the exercise program was successful for you?

P: Ok, I would um, judge it by, you know, seeing if it was first of all uh, safe, you know, are they, you know, um, just making sure that, that I was doing it uh, properly, you know, to be safe. And, and if it accomplished what I wanted to accomplish.

I: Got it.

P: You know, considered to be successful.

I: Um hm. And so, would you, if you were gonna look at it from like, a whole program so, not just individually, but like, everyone that's within the program, that is exercising with you like, um, how would you judge if it was successful for everyone?

P: Ok, uh, well I would uh, say it was successful if uh, if the majority of the people in, you know, accomplish what they wanted to accomplish. And, well, you know, that it uh, to me then it would be successful.

I: Ok. Um, and so, the second to last question is: what is your experience with exercise instructors and what do you like about them and what don't you like about them?

P: I like when they uh, when they are firm, you know, and they, you know, don't let us get away with not doing what we should be doing. You know, cause [inaudible] some people are not as firm and strict as others. You know, I don't want them to be too strict, but strict enough, you know, like really firm, making sure because they generally know what's really good for you and how much you have to do, you know, in order to accomplish what you're trying to accomplish.

I: Um hm, so you want them to be, keep you accountable kind of thing?

[00:29:59.4]

P: I didn't get that.

I: So, you want them to keep you like accountable, but not be too strict.

P: Right, right, right, right.

I: Ok.

P: Uh, let me speak to that question you asked just before, you know, what would I consider successful, is just still involving kidney disease, you know, cause that's just really the, sort of the focus, how it's affecting your kidneys. I would, I would consider it successful if it was one that [inaudible] or did, you know, make some improvement uh, you know, a lot of improvement in your kidney function.

I: Um, ok. Alright, no, that's, that's very important, so, um, so, what would you, are there, is there anything else that you would dislike or like about an exercise instructor? Something you would want him to do um, anything else?

P: [Inaudible] again where I'm not understanding something, what you're saying.

I: Ok, so um, in regards to the exercise instructor, is there, is there anything else that you would be um, that you would like them to do like?

P: Uh, that if, if they, if they had uh, since they are an exercise instructor, if they had any knowledge of uh, you know, of kidney, kidney disease, I think it would be very helpful if they, you know, would give us, you know, what information they could concerning, you know, the kidney system, you know we're focusing pretty much [inaudible] is focused, you know, on your kidneys. Of all people with kidney disease, that they could give us information that is really gonna improve our kidneys, you know, especially, I mean just anything. You know, even to eat, down to eating. You know, what we should be eating and what we should not be eating, to me that would be very helpful. That's one of the things that I'm really, you know, uh, I think I was told that the program, that um, eventually we will begin to, I may be mistaken, but I thought I heard them say we would, we would eventually get to the eating part of it. So, we'll, we're not there yet, so I feel like I have the exercise down pat, but I don't feel like I have the eating part down pat as I should.

I: Ok. So, having an exercise instructor with, who could help you more with, with more than just exercising. So, with your eating, with more health-related information.

P: Right.

I: Ok. And so, um, what things would discourage you from exercising? In the class.

P: If I, ok, you mean actually coming to the exercise class?

I: Or yeah, or just like, participating in it like, while you're participating in it kinda thing.

P: What would discourage me from participating in it?

I: Um hm.

P: Uh, if I wasn't getting any type of results.

I: Ok.

P: Uh, that would discourage me um, from continuing to come to exercise class. Um, that's the only thing I can think of at the moment that would really discourage me.

I: Ok.

P: If I'm not getting the results uh, you know, or what they say was gonna happen didn't happen.

I: Um hm. Ok, I'll keep that in mind. Um, and so, we have a couple more questions. Um, this one's about goalsetting, so, how helpful is goalsetting for you when you exercise?

P: I think goalsetting would be very, very important, I feel like, because I've experienced that, " What I said goes" or [inaudible] and then really try to follow those goals, you know, that's what's been most helpful to me. You know, and so, because when you don't set goals, you know, you just kinda get, you know, you don't have anything to go by or you just kinda like, can't do anything, you know, [inaudible] do it, you know, when you don't set goals. So, I think goalsetting is very, very important and just so, I think it could be a part of your success, you know, to set goals first, and then try to carry out those goals.

I: Got it, yeah, that's very important. And so, the last question um, I was gonna ask you is just about your experience with mobile phone apps and mobile phone exercise apps. And so, what are some things that you like about them and, that would uh, encourage you to use them and what are some things you don't like about them?

[00:35:08.5]

P: Ok. Well uh, the things I do like about them, like, what I like so far about them is I can uh, you know, look up, you know, my uh, Garmin uh, app and see, you know, you know, what I've accomplished and, you know, if I uh, and I could look a lot of times and see if I, how much more I have to go to uh, accomplish my goal for that day. And uh, so, I like being able to tell, you know, to see my progress. Um, and uh, let's see what I don't like about them? Uh, well I'll tell you one thing that's going on that I kinda uh, didn't like um, that, this probably doesn't have a whole lot to do with uh, the exercise part of it, is uh, when you like, it has a goal for your sleeping now. I don't know how important that is, for what we are doing, to our program, but, you know, uh, I, I'm very seldom meeting my goals for sleep is concerned. But one thing I noticed that uh, it doesn't do [inaudible] a lot of nights. Uh, I fall off asleep some days about seven or eight o'clock, and then of course I wake up and then I end up not going to bed until eleven, but it, but it'll show my sleep from eleven til five in the morning or six in the morning when I get up. [Inaudible] whatever time I get up, it shows that, but it doesn't show, at least I don't think it shows the, the length of time like, if I was asleep from, from seven to nine, it doesn't seem to show that part of when I was asleep. It doesn't add that to it. I kinda don't like that.

I: Got it. So, something that would, would um, record all your sleep that you've had...

P: Right.

I: Ok.

P: Right, cause I think, I think, I'm not positive, but I think that Fitbit used to show all of it.

I: Hm, ok.

P: You know, but this, this one, that it shows what time I went to bed and what time I woke up and that's it. You know, but I had those two hours nap before I actually went to bed.

I: Um hm, ok. Well that's important to note and maybe we can look at that um, as we go forward. But um anyways Ms. Name that was the last question. And so, um, I just wanted to thank you for all the helpful information you gave me and is there anything else you want to share with us before we um, stop the interview?

P: Uh, let's see. Uh, I don't, I don't know how important this is for exercise or uh, anything else that we'll try to do, try out during the research, but um, oh, I think about three to four years ago, I was uh, diagnosed with uh, I'm not sure if I'm saying it correctly, but sleep deprivation or sleep uh, but anyway I end up having to be on a C-Pap machine.

I: Ok.

P: And uh, and I, and I stayed on it, [inaudible] for the insurance to pay me for it, then once that was over with, you know, I didn't sleep on it anymore. And so, uh, I um, just recently started back sleeping with it because I kinda thought well maybe, you know, this could help my [inaudible] uh, you know they got me, well actually what made me start sleeping back with it is my son had came and [inaudible] at me and said "Mama, do you know when you're asleep, you know, that you're breathing kinda funny, and uh, you might need to go to the doctor." [Laughter from both] You know, to get that checked out and so I said, "Well actually I'm supposed to be sleeping on a C-Pap machine", but I really don't like to sleep under that thing, I can't stand it. But anyway, I decided, I made a decision to start back sleeping under it and um, so I'm sure my breathing is probably better, but it seem to affect me as far as exercise is concerned, but I don't, I don't know if it's affecting me some other type of way. So, you know, but I just decided to just start back using it anyway, so I didn't [inaudible] since I made the decision to start back. Uh, so that's the only other thing that I wanted to share with you. And I just actually thought about it, you know, when you talked about, when you talked about did I have any health problems that would cause me, you know, not to exercise and all that. I didn't think about that, you know, at the time we were talking about it, so I thought I'd mention it now.

[00:39:54.2]

I: Ok, yeah, no that's definitely important and um, I would say um, once we do start exercising, doing the program, um, just relay that to the instructor and be like "I have, I have to deal with this, and so, is there anything, any tips you can give me or um, sorts of advice?" And see if they know anything, but yeah, I think that's definitely important so thank you for sharing that.

P: Right, ok.

I: And so, yeah, so that, that's it for all the questions I have and um, I know that um, you'll be starting soon and hopefully the experiences um, you shared with us today will help make exercise easier for you and also for other people with kidney disease. And so, um, if you have nothing else to say, I'm gonna stop the recording here.

[00:40:54.0]

**Audio File Name: E-10 1-7-2019.mp4**

**Recording Date: 1/7/19**

**Interviewer: AB**

**Transcriber: BB**

**Dates: 4/12, 4/13 2019**

**File Name: #10 CKD Interview Emory University (1-7-2019)**

[00:00:00]

I: So, this is interview 10 of CKD study. Um, and so Ms. Name, can you tell me about a time you were physically active that you really enjoyed?

P: Uh, I'm physically active all the time. I walk, I do uh, Zumba, and I also lift.

I: And you also lift?

P: Yes, so I'm extremely active.

I: Ok, that's good.

P: If my lupus allows.

I: By, sorry, what was that?

P: I said when my lupus allows, I'm extremely active.

I: Ok. Um, and so what about being physically active do you enjoy?

P: Just being able to do it. [Laughter from both] Um, hm.

I: Anything else?

P: Being able to get out there and actually move around. Activity in general is just good for you, it feels good, it makes the body feel good, it controls weight.

I: Ok, so you, you like the way it makes you feel?

P: Uh huh.

I: Ok. And so, when someone says the word exercise to you, what do you think of?

P: Positive.

I: Positive, ok.

P: Positive, healthy.

I: Positive and healthy, anything else?

P: No that's about it.

I: Ok.

P: I don't know, I may not be answering your questions very well, because it's just something I always do. It's normal, like it's not, it's not uh, a chore for me to go and work out.

I: Ok. No yeah, no, there is no right or wrong way to answer the questions and these are just introductory ones, so um, since you do exercise a lot, um, I hope you will be able to provide me a little more information on our next question. Which is um, asking you to um, talk about your current exercise routine and specifically like frequency, the types of exercise you do, and just provide as much in-depth detail as to how you work out. And so, from what you've said so far, you work out a lot, and so, if you...

P: Uh huh, I had stopped for a little while because my blood pressure was up and uh, I kept getting dizzy and stuff, so I just started working out again.

I: Um hm.

P: And how I do that is I do cardio. So right now, every day, I go for an hour to walk on the machine or to the park, and then about after a month of me doing that, I'll feel a little more comfortable, my uh, stamina will be back up. And then I will go to boot camp and I normally go to boot camp two or three times a week. I have a trainer that I go see a few times, an actual uh, boot camp that he runs, and I do that. And I have two fake hips and some other issues, so he does things that work for me.

I: Ok.

P: That I could do. Like, he has a stand-up program, but then he adjusts it based on what I need.

I: Ok. That's awesome, I'm glad you have someone that works with you. Um, could you explain a little, a little more what like, kinda exercises you do at boot camp?

P: Um, I do a lot of lifting, I don't do any jumping, running, or jogging, cause I can't do any of that with my hips. And we really focused a lot on the lifting, and because I had [polymyositis?] in a particular [inaudible] in my arms, he focuses, focuses a lot on me lifting uh, for my arms and my back and my upper body to strengthen it.

I: Ok. So, a lot of strength training?

P: Yes, a lot of strength training.

I: Ok. Is this your usual amount of physical activity?

P: Yes.

I: Ok. So, how, I may have missed this, but how many days a week do you normally um, exercise?

P: Um, I try to do at least two or three days a week.

I: Ok.

P: I am really on the ball, and if I have a lot of energy then I'll do at least four. I really decide that based on just how I'm feeling. So, I may work out a lot and then there's going to be times where I don't work out at all, and then there'll be times that I'll work out in between that. I really just base that on how I'm feeling with my lupus.

[00:05:05.5]

I: Ok. And so, I know you've said you've, so you have lupus, right?

P: Um hm.

I: And so, um, I'm curious to see like, in, like in addition to like CKD like, how did your exercise routine change when you were diagnosed with these diseases?

P: Well I was diagnosed with lupus all the way in 2001.

I: Oh, ok.

P: Um, yeah, I've always been an exerciser. So again, it really just focuses on how I'm developing.

I: Ok.

P: And how much I will exercise.

I: Ok. And so, when you were diagnosed with CKD did anything change or?

P: I almost died.

I: Oh. [Laughter from both] But you're here now.

P: I did not know I had lupus, I got rushed to the hospital because my kidneys failed.

I: Oh wow. And so, because of the lupus, is that, you also have kidney disease now?

P: Yes, I have kidney disease due to lupus. I have lupus [inaudible].

I: Ok. Well, I'm sorry to hear that but I'm very glad that we're having this interview now, so...

P: [Laughter] So am I.

I: Um, so the next couple questions are gonna just talk about your, the perceived, or your perceived benefits of exercising. And I know you talked about this a little bit earlier, but um, could you talk about some of the health benefits that you have experienced in exercising and other, other benefits?

P: The biggest health benefit for me is um, helping to control my lupus. So yeah, that um, that's the biggest health benefit, controlling my lupus uh, semi-controlling my weight when I'm on all of the different medications that I've had to take over the years. Uh, I had both of my hips replaced so I did a lot of rehab, so exercising helped a lot with rehabbing.

I: Ok.

P: And um, [inaudible] uh, I'm sorry, I'm talking to you and doing this [inaudible].

I: It's ok.

P: Um, um, what was the question again?

I: So, what are some of the benefits of exercising? And I, you...

P: Oh, ok.

I: And you said a lot already, um, are there any other benefits that you can think of that aren't health related?

P: Um, you know it gives, it gives good piece of mind. A lot of times when I exercise, I meditate while I'm doing it, so it's good for relieving stress. It's good for meditation um, clears the mind, helps with sleep. On days that I work out, I sleep better.

I: Um hm. Yeah, perfect thank you.

P: You're welcome.

I: And so, the next question is asking how important is exercise for patients with kidney disease?

P: For myself it's been very important. Um, I just really feel that it has helped me keep things under control, because it's keeping me active, it's keeping me focused. Um, I know that if my kidneys have improved, [inaudible] diagnosed. And I actually have to get another kidney [inaudible] in February, so I can see where they are now, cause my last [inaudible] but um, in general, I just think being active, period. Helps the body improve, build stamina [inaudible], build the stamina, um, build up the body uh, helps to [inaudible].

I: Uh huh. And so, um, when, does your doctor talk to you about exercise or kidney, like, give you exercise advice on how to manage your kidney disease?

[00:10:04.2]

P: They did not but that's only because I've already been proactive about that and I already do exercises and stuff so [inaudible].

I: Ok. So since...

P: But it is definitely something that's discussed more than in the past.

I: That's it, that's good, ok. And so, what kind of advice, when they do give you advice, what kind of advice do they give you?

P: Um, basically they just say uh, they just question me about my activity level and then once, once I tell them how active I am, then it's really not a conversation anymore.

I: Ok. They're like, "Oh, good job" kinda thing?

P: Yeah, yeah but also, when I was first diagnosed um, doctors never discussed being active at all. So, it's definitely a change on the doctor's part, understanding how important uh, being active is to [inaudible] if you're able to be active. Cause everybody [inaudible] not able to be active.

I: Ok. And so, I, I know you, for the next question, I know you were saying that you have two um, was it artificial hips or a hip replacements?

P: Yes. Um hm, I have two.

I: And so, and you said that makes it hard for you to, to walk, right? Or to do some like...

P: Uh, no, not, it definitely [inaudible] walk, cause I rehab, and I walk fine, unless I'm tired. I can't run, jog, or jump.

I: Run, jog, or jump, ok.

P: Correct, cause I can't put any pressure on [inaudible] pressure on my hips because I want them to last as long as possible and also because, because I have to [inaudible], if I do feel like it causes pain in my joints, which causes me not to feel well, which can cause swelling, and then I have lupus issues, so I have to be very particular about when I [inaudible] and how I do it.

I: Ok. And so, besides health barriers, are there, is there anything else that makes it hard for you to exercise, such as like time or...

P: Um, one of the other things is when you’re dealing, when I'm dealing with lupus, it uh, affects the joints. So, I have to be very careful of certain exercises I do so it doesn't stretch it out, over-stretch my [inaudible].

I: Ok.

P: Or cause myself pain, so I have to be very cognizant of certain exercises that I do.

I: Ok. And from what you've told me, it sounds like you're very motivated to exercise, but are there times where it um, it's harder for you to exercise because of motivation or...

P: Oh yeah, it gets difficult at times and that's, sometimes I just don't feel well, sometimes I just don't feel like being bothered. Just like with, with anything, you have to self-motivate, you have to constantly[inaudible] the benefits are to you, doing what you’re doing. You always have to focus on benefits, and understand what the benefits are, so for me, working out means having better, better life uh, having more energy, not being overweight from medication, not uh, being a little more stress-free.

I: Um hm, perfect. Um, yeah, I definitely feel those things when I exercise too. So um, and so for the next couple questions we're gonna talk specifically about the program. And so, we are considering um, you already know this, we are gonna create a program that's group-based, and so, what do you like about group classes and what don't you like about group classes?

P: Uh, from what I do know, I do group classes but I [inaudible] like the trainer gives me stuff to do, and I do that, but I do stuff at my own pace. So, it just depends on the levels of ability of the other people in the group.

I: Um hm, ok.

P: Like I work in groups, but I don't work in groups, if that makes sense.

I: Yes, so you would like to go at your own pace?

[00:14:55.7]

P: Yeah, like I can basically, this is what, how my trainer does it. He has a routine for everybody for the day, for that day, but everybody works at their own pace, cause everybody's not at the same level. We can all do the same routine or as I said, he adjusts stuff for me, but everybody goes at their own pace. As long as they get [inaudible].

I: Got it. Yeah, ok. And so, um, how do you feel about an exercise class created especially for people with kidney disease?

P: I think it's a very good idea.

I: Um hm. And so, do you think it would make a group class more, better, would it be, make it more difficult?

P: Um, it is all gonna depend on the capabilities of each person. And it's also going to depend on the person who is teaching the class and them actually doing [inaudible] and being able to work with people at different levels.

I: So being able to work with people at different levels. Do you have...

P: Uh huh, because when you're dealing with people with chronic illnesses, everybody's not going to be able to do the same thing. So, say they have a particular exercise in mind for everybody to do, and everybody can't do that exercise. Then they have to already have some alternative involved that are going to work the same muscle groups or whatever, in order for everybody to actively be involved.

I: Um hm, oh yeah, definitely. Um, and so, so for um, the program are, do you have any transportation barriers?

P: I do not no. [Talking with someone else]

I: So, no transportation barriers, ok.

P: No none at all.

I: Alright. And so, would you... [Participant speaking with someone else]

P: I'm sorry, go ahead.

I: Um, no worries. And so, I know for your current boot camp class, you do a lot of strength training, and so, would that be the kinds of activities you would also like to do, or would you like to do uh, different kinds as well?

P: Um, I would consider doing anything and trying it once, but I would probably focus on the strength training. I'm always open to new ideas, [Talking with someone else].

I: Got it. And so, what would you value most from the exercise program?

P: Uh, huh, that's an interesting question cause I could change from week to week. Um, the most important thing I would say, would be the calm that it brings me. Exercise relieves stress for me, so that's really the most important thing I'm looking for when um, I'm exercising.

I: Ok, so the calm that it brings you, reducing stress that's what you would value from it.

P: Um hm.

I: Ok.

P: Because the, because uh, the less stressed I am, the better off, the better I feel. And the less likely I am to get sick.

I: Got it. And so, how would you judge if the exercise program was successful?

P: Um, for myself, again it's gonna be based on how it makes me feel, if it continues to, to help me um, control my lupus. Um, if it keeps me in shape, not even necessarily losing weight, but you know, keep me from, from gaining weight. Um, just in general uh, a basic overall feeling of feeling good.

I: Ok, and how would you judge if the program was successful in its entirety? So not just individually, but um, for everybody.

P: Any program that has people get out and be active is successful.

I: Um hm, ok. And so, just, just by having the program, it's a success already?

[00:20:01.3]

P: Um hm, just by having the program and, and giving people the opportunity to have some place to go, and also teaching them how to work out and what they can and can't do. A lot of times people don't work out because they have no idea how to get started.

I: Um hm, so this is like a good starting point for some people?

P: Yes, and, and a lot of people know they need to work out, they just, they just don't know what, what to do, how to do it.

I: Um hm, ok yeah, I know, I agree with that. And so, you have a exercise instructor now um, so what, what do you like about the exercise instructor and what don't you like about them, or like, maybe not your specific exercise instructor, but just in general, like a good exercise instructor would do what?

P: In general, they'd listen. And particularly when you have a chronic illness, they listen, because I could go to class [inaudible] do everything and [inaudible] the extra. And then two weeks later, I can come back and not be able to do anything, and most trainers don't understand that concept because they're trained that your supposed to make gains every single week and that does not work for people who have any type of chronic illness because once you can do [inaudible] how you feel. So, the most important part is having a trainer who listens and who understands when, when um, a person really can do something and when they really can't. And understanding when they need to change the exercise to different things, and they also have to understand that pain is not gain. They have a chronic illness, a person has a chronic illness and still in pain, there's a problem.

I: Ok, and what things would discourage you from exercising?

P: If I don't like the person.

I: If you don't like, if they don't do the things that you just mentioned?

P: Right, if I, if I tell them that I can't do something and they keep reiterating that that's what they want me to do or they continue to try to get me to do it, or if I tell them I'm in pain and they don't listen.

I: Ok. Yeah, our exercise...

P: The thing is I'm very forward about it, someone else who was new at it might be afraid to say that there's a problem.

I: Ok, perfect, that's, that's really good to know. Um, and so, we're at the last question, and this question is just gonna talk about mobile phone apps. And so, have you used the mobile, like a health app on your mobile phone before?

P: Um, I have, I don't like them.

I: What don't you like about them?

P: They just, because I have the issues that I have, [talking to someone else] they just didn't really benefit me.

I: Ok. And so, which ones did you use in the past?

P: Because, um I've actually used, I don't even know the names of them, but just ones that other people have. Um, the only one, the only one I [inaudible] that I stick with is the uh, not the Garmin, the other one.

I: The Fitbit?

P: Say that again?

I: The Fitbit?

P: Yes, the Fitbit, yeah, but sometimes it messes up.

I: Ok. And so, are you currently using an app?

P: I am not, I just do what I, I just do what I do.

I: Ok. And so, is there any way that you can think of that um, would make it easier for you if we had you use apps?

P: [Inaudible] app, because the most apps, apps are made for people that are healthy.

I: Yeah.

P: So, a lot of the stuff that they put on those apps, you, you can't do. [Laughter]

I: Um hm.

P: That's the biggest problem with apps, there's no true app that's made for someone that has a disability or health issue, they're all made for healthy people.

I: Ok. And so, if the app was just used to track your activity, it wasn't one that gave you specific kinds of exercises to do, it just measured your steps and measured your sleep, is that something that would be easier?

[00:24:53.4]

P: Um, it would be easier for most people, but in the long-term it's not gonna really benefit them because at some point you have to add on other stuff. I think the biggest thing is it means to, [talking to someone else]. I'm sorry, um, at some point you have to add on exercises, the problem with, I guess the best way to set it up, if you're working on a specific body part, you need to offer four or five different exercises for the person to do. Instead of just saying we're working on upper arms and this is what we're going to do, because everybody's not going to be able to do that.

I: Ok. Got it, well...

P: More choices.

I: More choices, ok, choice is key. Perfect, well Ms. Name, that is the last question, and so, thank you so much for all the helpful information you've gave me um...

P: You're welcome.

I: Is there anything else that you want to share with us before we end the interview?

P: Um, nope that's pretty much everything, but if in the future you need to call back to [inaudible], let me know, I'll be more than happy to help out.

I: I, I really appreciate that, uh, and yes, we will contact you soon. I think that we're about to start up this program um, sometime this month.

P: Ok.

I: Um...

P: Yeah, they're, I'm supposed to be coming in on the uh...

[00:26:39.5]

**Audio File Name: E-11 1-29-2019.m4a**

**Recording Date: 1/29/2019**

**Interviewer: AB**

**Transcriber: BB**

**Dates: 4/19/2019**

**File Name: #11 CKD Interview Emory University (1-29-2019)**

[00:00:00]

I: CKD interview number 11. So, could you tell me a time when you were physically active that you really enjoyed?

P: Well, the time I remember is about, I guess would be about ten years ago. I was in the um, this optimal living program that Emory had, and it led into, once you finished optimal living, you could do the Heartwise program. Do you know about that? It, it...

I: Could you talk more about it?

P: What?

I: Could you talk more about it?

P: Sure, it's a, it's, I think it's the American Cardiology Association, cause it's at different hospitals.

I: Ok.

P: And they have exercise physiologists, I think they have a nutritionist on staff, and you're supposed to go three days a week, but for some reason they let me go four and five days a week. And you have a little plan and you have different um, [messes?] of exercise like, there was a little track that you could walk around and there were treadmills and there were bikes, I don't think there were recumbent bikes. I don't remember. And there were also weights, I never liked the weights, I know they're really good for you, but I never liked them. You know, the machines, I don't think I would mind free weights, but, you know, just little hand weights, but I hate those machines.

I: Ok.

P: I know they're, I know they help you, I know they help you a lot, but I hate them.

I: Ok.

P: Um, even though I had been trained, you know, but it was hard for me to remember how to do each one, I don't know if it's my age or what. And, you know, I'm just telling you the last acceptable exercise program I was in.

I: Ok.

P: Because, I also swam a lot when I was younger, but I barely remember it, you know? So, is that ok?

I: Um hm, yes that's perfect. Um, and so, what kinds of activities did you enjoy during that program?

P: Um, I really liked the bike and I liked the treadmill and I liked walking around the track.

I: Ok.

P: And um, I think that was what I liked. And I liked the fact that I was younger then, ten years ago, and it was an older group of people in the main and some of them were really old. And I loved seeing, even though I wasn't necessarily part of the community, I loved seeing the community.

I: Ok.

P: I loved seeing the older people, some of these people would walk in on a walker and get on a bike. And it was just uplifting.

I: Um hm, very cool. Um, alright yeah, that was a great answer, thank you. Next question is: what do you think when you hear the word exercise?

P: I think I need to do it ugh, that's what I think right now.

I: That you need to...

P: I need to do it ugh, ugh like, ugh I don't want to do it.

I: Oh...

P: But I know if I do it, I will feel better and it's just getting going.

I: Ok.

P: I haven't had a hard time getting going in this program, and really one of the reasons is the weather. The weather has been, I was going to walk, cause walking's so, you know, convenient, and I've moved this way, so it has great places to walk. But, it rains so much in December and then when it wasn't raining it was so cold, I was afraid to go out there, but just the other day, well just this weekend, my sisters were here and I knew that, and in the bottom of the building is a little exercise room. And it has a treadmill and a bike, it has some free weights and it, anyway, it's very small, but I thought I need to learn to use, and, and I talked with [Venicia?] about this, I'd forgotten how to use a treadmill. I'd forgotten how to get started on it, you know? And I imagine if I would just google "treadmill", I can get the basics down and it would come back to me, and that way I wouldn't have to worry about the weather.

I: Um hm.

P: And then, when I get in better shape, I won't be as afraid to walk away from the, from [soon?]. You know what I mean?

I: Yes, I do. What...

P: [Inaudible] right now.

[00:05:00.1]

I: Yeah, weather definitely makes a difference so, thank you for sharing that. Um, and so...

P: And thank you for thanking me.

I: Yeah, you're welcome. Um, so, the next couple questions are gonna talk about your specific um, physical activity behaviors, so like what you're currently doing. And so, could you tell me about your current exercise routine?

P: I really don't have one Alan, I, I, I have, I can't find, I...

I: You do walk though right?

P: I wanted to get started on walking, but I didn't. One of the things that I really noticed lately is I need to stretch more.

I: Ok.

P: I'm um, I get frozen, I don't know how to explain it. If I sit too long, my joints get frozen. And I know, like in, I sleep a lot and, in the morning, I need to stretch before I get out of bed. And those are things I could just look up on my own, I don't need a trainer to do that, but I don't know if you can understand it, being young. But something happens to your muscles, they like get um, stuck, I don't know how to explain it, but I know I would feel better and I know I would feel more secure if I stretched.

I: Ok.

P: And uh, anyway.

I: And so, how often, like currently, do you stretch in the mornings?

P: I just started doing it in the mornings, just for about five minutes, and then as the, in the bed, and as the day goes on, I'll touch my toes, you know, and just hang, have my head hang down. Um, but it's very haphazard.

I: Ok.

P: [Inaudible] the program.

I: Ok. And so, you would like to do more stretching um, I know you said earlier, you walk a lot depending, or you walk depending on the weather. And so, currently like, in the past week, how many times do you think you've walked?

P: None, that's what I meant.

I: Ok.

P: Because of the weather, I haven't really started walking.

I: Ok.

P: Do you see? It was so, and part of it is I've had a lot going on. I sold my old condo, I'm still settling into this condo, and I probably shouldn't have taken part in the program, because I, I don't have the time to do it, but I know I need to be doing something. But I hope I don't blow the curves.

I: Oh, I'm sure you won't, don't worry about that. Um...

P: But um, I am going, I am planning to look up how to use that treadmill at the little gym in the building, cause I'm lucky that I live in the building. The other thing Alan that I need, I don't know if this is appropriate, the other thing I keep thinking of is if you're older you have to get a supplemental medical policy for your Medicare and mine has this thing called silver sneakers and I can go to so many gyms, you wouldn't believe, for free. And what I'm thinking about doing, is going to one of those and you can hire a trainer there. There's one in Decatur, little, used to be called Dekalb General, but I feel like those exercise physiologists will be um, I don't wanna get like an LA Fitness muscle person, I just want someone that, there were exercise physiologists in that Heartwise program and I really respected them. They worked um, I, I, I, I imagine you know what I'm talking about. There's a trainer for young, healthy people, but an exercise physiologist is almost like a physical therapist. They know how, the limits of different age groups.

I: Um hm, ok. That's, that's good to know and later in the interview I'll be asking you more about like, the specific types of exercise instructors that you would prefer. And so, um, we can go into more detail about that later. Um, and so, back on your current exercise routine um, prior to when you were diagnosed with kidney disease, was your exercise routine um, different? Um, was it easier to exercise? Um.

[00:09:52.7]

P: You know, I think it was. I know that sounds like a big um, cop out, but I've only been diagnosed for five years. I think I had it um, long before I was diagnosed, but when I was in that Heartwise program, I really loved exercising. And it was before I was diagnosed.

I: Ok.

P: And I told you why I liked it.

I: Yes, you did.

P: It was, it was all about, but then Emory shut the program down, and I'm still mad at Emory about it.

I: Um ok.

P: They moved it, I don't know if it's appropriate to tell you that, but they moved it to St. Joe's. And I live across from the law school, so asking me to go to St. Joe's three days a week is like taking a day trip.

I: Um hm.

P: And, and it, I'm hopeful they'll look at this new Dekalb Medical Center and get something going on this part of town. I mean this is Emory's core right here, not St. Joe's.

I: Um hm.

P: Anyway, that's just my opinion, I should shut up.

I: No, no, no, no. I welcome all your opinions ok?

P: Ok.

I: Um, can I ask you um, when that program got shut down?

P: I think it was about five years ago, I can't be sure about any dates, but I think it was about five years ago. And it just was all of a sudden, and it was in the employee gym, the employee gym is at 1525 Clifton, at the top, it was just a lovely, small little gym. They didn't feel exposed or anything. And they said they needed the space, I guess for the employees, but it was, I mean I've been at Emory, I was a college student, I worked in the medical school for a long, long time, it was just, the way they did it was so typical Emory. Just like all of a sudden, we don't, you know, one of guys in the program who was younger said "The sad part is some of these people may die because of not having this program." They couldn't go up to St. Joe's and that community was keeping them going. You know, what I talked about is, even though I wasn't part of it, just seeing it was so powerful, and um, and it was like well they didn't care. You know, they don't care, it's, anyway, I love Emory, but I hate them. [Laughter from both] I mainly love it, but that was really, they showed no concern for the health of the people.

I: Um hm, well that's unfortunate. Um...

P: It is unfortunate, it's very unfortunate.

I: Um hm, well we will keep that in mind. Um, and so moving on to the next couple questions, they're gonna talk more about um, exercising in general. And so, in your opinion, what are some of the benefits of exercising?

P: Well, physically, you get energy from exercising. And the business about needing to stretch, I think you're more coordinated when you exercise. And I think, you know, every time an older person goes to the doctor, they ask if you've fallen in the last six months, I think Medicare requires it. And I think exercising prevents falls because you're stronger, and [Venicia?] actually told me fear of falling is part of the reason fall. So, I'm not sure they should be asking us if we fall because I, and I do have a fear of falling, but that's another issue. The other part of exercise that I think is important for me is the mental benefits. I, I have, can't say I'm depressed, I was diagnosed one time with dysthymia, which is like low-grade depression. You know, and I was, it helps more with that, more than, I've taken antidepressants and they don't, they don't really help with dysthymia, but exercise does help. It helps reduce stress.

I: Perfect, and so, would you say those benefits motivate you to be physically active or is there another motivator in your life?

P: I think they would be my motivator, I'm just not listening to them right now.

I: Um, ok. Um, would there be anything else that would motivate you to be physically active?

[00:14:52.6]

P: I think having that group again, that community that I saw before. A community of like, people like me. You know, for me to go to LA Fitness, which I can go to for free, being overweight and older, is like what? Do you understand what I mean by that?

I: Yeah, cause....

P: So, having this community of older, like-minded people, you know, and some of them may be stronger than you are and some of them may be less strong, but the community, community is really important.

I: Um hm, perfect. And so, I mean that's kinda what our program is about, right? So, it's, I'm glad...

P: No, I don't see the community, I mean when I went down there for my second set of measurements, there was a lady in there and I should, anyway she, she was on a walker, but she really wanted to exercise. And I was like, "Wow, she's gotta walk for six minutes on a walker." It was really hard for her, and so, I told the guy, "I don't think I'm gonna do as well I did the first time, Ahad." I said, "I don't think I'm gonna do as well as I did the first time." Cause I was tired, and my shoes weren't right, and he said I did better. I mean, he, he was going from memory, but I think it was I thought, "Well if she can do it on a walker, I can do it with two legs." You know, that I don't need a walker, and that's the benefit of community, but in this study, I don't get the benefit of community, that was just a fluke thing.

I: Ok. Um, so once, once the actual sessions like, start, hopefully you'll start feeling more part of, or seeing the community more. Um...

P: No, I'm not in that group.

I: Oh yeah.

P: I'm in the motivation group.

I: Ok.

P: Which is a joke, frankly. I mean [Venecia's?] nice, but I don't get the benefit of community.

I: Ok. Ok, so we'll keep that in mind. Um, the next question is gonna ask you more about how important exercise is for patients with kidney disease. And so, has your doctor ever talked to you about exercise and kidney disease management?

P: He has. He um, he mainly talks to me about the need to lose weight.

I: Um hm.

P: He talks to me about exercise, but um, mainly he talks, anyway, he's a good doctor.

I: Um hm. And so, what kind of exercise advice does he give you or does he?

P: Well I can't remember something, I think he said that I should get a trainer. Cause I can join the Emory, the big gym at Emory, you know, whatever it's called, Woodpec or whatever. Is that what it's called?

I: Um hm.

P: [Inaudible] it's called, I can join there, and he said the trainers there are pretty good. I can hire a trainer, that's what I remember.

I: Ok.

P: We don't talk about exercise every time.

I: Ok. Alright, and so, next question kinda talks more about exercising, but in this instance, what are some of the things that make it hard for you to exercise? And I know you've touched upon this a little bit, so.

P: [Laughter] This right now is the weather.

I: The weather.

P: The other thing is, I go through these periods and I've been in one for about the last six weeks, where I can sleep 12 hours a day. Where I am just so tired, I've never talked to Dr. Name about it, because it's not all the time. It's just these stretches that I'll just, I think it's maybe an escape, I'm not sure. You know, I know this sounds silly, but I moved out of this house that I'd lived in for thirty years, this [inaudible] home into this one-bedroom place and I still, it's been really traumatic to get rid of stuff for thirty years. And I still, even though I sold the other place, I'm still looking at containers here that I need to go through. It, that sounds, I know a young person may not understand, but it, it's psychologically difficult to go through your life like that, you kinda don't want to. Once you start it's ok, but anyway that's been my main focus for a year, more than a year.

[00:19:58.9]

I: Ok. And so, now that you've moved into a new place, do you feel like it will be less of a barrier now or?

P: Yes, if I finish off these containers, I still have to go through it. I just feel like I need to get that done before I have a life. I know it's fixed on finalism, but it's, it's the truth.

I: Um hm. Alright, and so is there anything else that makes it hard for you to exercise, besides those things you've mentioned so far?

P: Um, for me it's always been just getting started and, and I don't know why I can't get started. Cause we talked about how I know it's beneficial, but I don't know. But this I know, right now that I feel like I've gotta get these boxes done. But do you know that term fictional finalism? It, it's a psychological term where you think everything will be fine when something is final, but it's not really true. [Laughter] Cause it's fictional, but anyway, it's a trick [inaudible].

I: Ok, and so those are, that's one thing that you're dealing with now.

P: Yes.

I: Ok.

P: I know that sounds petty, but for me it's not.

I: Um hm. Ok, well let's hope...

P: I hope I'm not boring you.

I: No, no, no, no, no, I'm just trying to formulate a good response. Um, in terms of just um, dealing with that barrier, I hope you do get it um, completed um, cause if that's what it takes to um, make you exercise more, then um, it'll be beneficial. And so...

P: Well I deactivated my Facebook account, so I can't waste time that way.

I: Hm, ok. Yeah, I need to do that too. [Laughter]

P: I think it's good to do every now and then. It's like a retreat, cause especially at my age Facebook is such a waste of time. I mean I like it, but it's a waste, you know, I can waste a lot of time. Yeah, that's one of my barriers to exercise in general, is I tend to waste time. Which, when you get older, you realize that wasting your time is wasting your life. I mean it's true for you too, but it, it's, you’re not as likely to do so at your age. You have school, you know, you have definite things you are required to do, but when you get older, you, you can't waste time cause you don't have that much.

I: Um hm, yeah, definitely. Um...

P: And I'm bad about wasting time, I've been bad about wasting time my whole life. I don't know what that's about. I had kidney disease as a child, I had kidney disease in the 5th grade, and I almost died. Um, it was called acute glomerulonephritis, and sometimes I think, I mean I was out of school for a year almost, nine, seven months. I'd have a tutor to keep up, even though it was 5th grade just cause of the laws, you know. [Inaudible] just had to go to school and I think in a way, that had more of an impact on me than I'm, than I know. And now, they found out that kidney disease in adulthood, can be caused by kidney disease as a child. I mean, that's not the only cause, but you know. I mean, so I think I'm kinda mad about that, you know.

I: Ok, yeah, I wouldn't, I would be too, so.

P: Oh good.

I: Ok, Ms. [Name], we're gonna move on to the final aspect of the interview. Um, these questions are gonna talk about specific um, program components that um, we would implement in an exercise program. And so, I know that you aren't in the exercise um, arm of the study um, but the information you do give us um, about your preferred exercise program will help us kind of um, create like, a more comprehensive one in the future. And so, um, I just want you to keep that in mind um, with these questions. And so, the first question is: how do you feel about group exercise classes? And I know you talked about this earlier too, how you like community in terms of um, an exercise program, but, so yeah what do you like, and do you dislike about group classes?

[00:25:07.6]

P: Ok, I like group classes. When I was young, when I was like in my thirties, I was a member of a gym that had, you know, it was the James Bond era, that kind of stuff, before leotards was in. And it was great, you know, to be around the old, I like that. And then, in this, I forgot to mention that in that Heartwise program, they also had classes. Stretching classes, um, regular classes that different people taught, and I loved that because that was when you also saw the community. So yeah, I liked that.

I: Ok.

P: I like people. I have to preface that because I don't think I would like the class, either a real mixed group, but I don't want a class for like 65 years old, I would want those classes I was in when I was thirty.

I: Hm, ok. So, things have changed since then?

P: Um hm.

I: Ok. And so, if you were...

P: I want like people.

I: You, you want what, what was that sorry?

P: I want the people in the class to be kinda like me. I don't want young, fit people in the classes and overweight older persons, you know?

I: Um hm, yeah, I get it. And so, that's one thing you would dislike about a group class if, if the class wasn't cohesive, kind of?

P: Well if it were a class of kidney patients, I would be happy, you know? Like there's some, some, I wouldn't be happy going to, I don't even know if the LA Fitness has classes, but I know I wouldn't want to go to an LA Fitness class.

I: Ok.

P: Because it'd be all these young, fit people and I would feel intimidated.

I: Um hm, ok. Um, yeah, I think that's very important. And so, can you think of anything else that you would dislike about group classes?

P: Maybe that I couldn't be free about the time, you know, I'd have to get somebody else to schedule.

I: Hm, I see. It wouldn't be like individualized?

P: No, actually if the class is small enough, you can get enough attention. You know what I'm, it's just, I don't know how to explain that, but I'm, I'm going back to that Heartwise program in my mind.

I: Ok.

P: And, if, in those classes there'd be maybe, sometimes there'd be 20 people and you could still get attention if you needed the attention.

I: Ok. Alright, we'll keep that in mind. Um, in terms of barriers to participating, I know you talked a couple, or talked about a couple already, but is transportation something that um, inhibits you from participating in a group class?

P: No, not yet.

I: Ok. So, you do have transportation, whether it's you driving or taking public transit?

P: Yes, I mean I have to buy a new car and I don't want to, but I, I still drive, if that's what you mean.

I: Ok, yes.

P: I don't like driving in Atlanta as much as I used to, but you have to do it.

I: Ok. So, next question is: how should we design these classes to be most convenient and appealing to you? And so, um, you talked about a couple of the activities that you would like, you said you like um, walking, biking, and then stretching was something that you also were interested in um, in pursuing. And so, were there any other kinds of activities that you would like to do in an exercise program?

P: Yes, I would like some classes on balance.

I: Balance, ok.

P: Strength and balance cause that's the real issue. Um, the other thing is, I've taken these um, classes called Ageless Grace, which you can take for free with Silver Sneakers, and their chair exercises, and you can still get good exercise in a chair. And at some point, you can stand up if you want to, but, and their real small, but I like those too, chair exercises.

I: Ok. And so, you do all the exercises sitting down?

P: Yes, they're designed, this thing called Ageless Grace is designed, I don't really completely understand it, but it's designed, the exercises are designed to stimulate different areas from a seated position.

[00:30:00.3]

I: Um hm.

P: And a lot of um, it's good for Parkinson's patients and it, and older people.

I: Ok.

P: It's called Ageless Grace.

I: Perfect um, we'll have to look into that program to see if there's anything we can pull from it. And so, um, what would you value most from an exercise program?

P: Um, just stimulation.

I: Ok.

P: Physical strength, and like I said that community if it's the right community.

I: Um hm.

P: I really wish, I've said this before, but I really wish Emory would reinstigate that Heartwise program in this area of town. Either it be employed in, or now that they have this relationship with Dekalb General, they should look at that center.

I: Um hm.

P: It's an incredible health, medical, wellness center they have out there.

I: Ok, and remind me...

P: I guess that speaks specific to the study to think about just Emory, but...

I: Remind me where you live again?

P: But that Heartwise program is really good and you have so much that's good for heart and good for kidney.

I: Um hm, ok. Um, we're definitely gonna have to look into that program again and then see if there are things that we can pull from it um, because it sounds like it was really beneficial to you and others, so. Um, the next question is: how would you judge if the exercise program was successful?

P: Whether I lost weight, number one.

I: Um hm.

P: Whether I mentally felt better and whether I felt stronger and more, and less, had less fear of falling.

I: Ok.

P: I guess for losing weight wouldn't be priority, it would be those other things.

I: Um hm.

P: Just know that exercise doesn't always make you lose weight. Exercise does, however, make you more aware of what you're eating, and you might lose weight from that.

I: Ok. And so, if a program was able to at least do all those things or one of those things, you would consider that successful?

P: Yes.

I: Ok. And so, what about if you like, what would it take for you to um, say the entire exercise program was successful, not just for you but for like, everyone involved?

P: Oh, I guess whether people participated. I know that sounds kinda basic, but, you know, if you have a program that makes people want to come, and that's set. It goes back again to that community feeling.

I: Um hm.

P: Community in our society is more important even then it was five years ago, I think. But, you know, I know that not everybody with kidney disease is elderly, but a lot of them are. And, um, isolation is a big deal, I'm not quite there where I'm isolated yet, but I can see how easy it is to become isolated when you're older. When you're not working, that's one of the reasons why I want to work again, just to thwart any kind of isolation.

I: Um hm, ok. And so, as long as people continue to want to go to the program um, want to exercise, that would be a success in your opinion, is that correct?

P: Yes, and part of the reason I say that Alan, is I know that kidney disease really can't be um, like my, my GFR number might vary, but it'll, I'll never be, I'll never be without kidney disease. You know, it's kinda, it's chronic, it's, it doesn't go away, you know, you can't exercise it away.

I: Um hm.

P: So, the, that's why you can't have the goal of getting rid of kidney disease.

I: Um hm.

P: You have to have these other things that make it successful, and it would be participation and community and feeling better.

I: Ok.

P: It can make you feel better but it's not going to get rid of your kidney disease, or maybe I'm wrong, but I don't, from what I understand it won't do that.

[00:35:02.1]

I: Ok. Yes, no that's uh, very, very important, so. Um, so we have a couple more questions um, this next question is gonna go back to um, your experience with exercise instructors and what you liked about them and what you disliked about them.

P: Ok.

I: And so, what are some of those things that you liked about the instructor and what are some things that you didn't?

P: Well, I liked, I liked the individual attention of the instructor to learning to use the machines. I mean, and by the machines, I mean the treadmill and the bike and stuff like that. And in that program, one of the exercise physiologists would be available just to check in with different people once they had learned the machine. And they would take our blood pressure at the beginning of the session and at the end, but during the exercise, they would come and check in on you. Like I also mentioned, the um, I think I just have a mental block about those weight machines. I never could, no matter how much they worked with me on them, I couldn't remember them, from time to time. And then, in a class setting, as opposed to those treadmills and things like that, I like an exercise instructor who's positive and maybe has some humor rolled in and also has the ability to look and see if someone's not doing something right. And the, and to be able to say, "Hey, let me come over here and help you with this."

I: Ok.

P: Even while the class is going on. Just positivity and, I can't think of a exercise instructor, oh yeah, I did, I did go to this exercise class at the senior center about a year ago. And the woman was like a drill sergeant and I didn't like her.

I: Ok.

P: It was a big class and she was like, I don't know how to, she was just like a P.E. teacher in the 9th grade, you know?

I: Oh, I see, I see.

P: Yeah and I didn't like that at all, it made me feel bad.

I: Ok so, was it like very, very structured and um...

P: Very structured, a waste of time, I mean I was able to keep up, but it was just, it was a class of seniors and I just felt too, like the army. I felt like I was in the army.

I: Um hm.

P: Basic training.

I: [Laughter] Basic training. Wow, sounds, sounds very intense.

P: Not that I've ever done that, but you know what I mean.

I: Yes, I do. Um so, less intense of a program, less intense of an instructor, somebody that is positive and encouraging and will also help you improve your form. Um...

P: Right, aware, well this woman wasn't aware of anybody in the class.

I: Ok so...

P: Which is [inaudible].

I: So, what...

P: [inaudible]

I: Ok. And so, an instructor with some awareness of who's in the class and level in the class.

P: Right and have some feeling.

I: Ok. Have feeling, got it.

P: And I guess in that case it was a, like I said it was a big class, it was probably 30 people and maybe 30 is too many.

I: Um hm, ok.

P: Maybe 20 is a better number, I don't know, I don't know.

I: And so, are there anything else, or is there anything else that you can think of that would um, discourage you from exercising, in terms of uh, the exercise instructor?

P: Um, I'm trying to think of bad instructors throughout my life. I really, I, I, she was the only one that I thought was bad.

I: Ok.

P: Most of them had some redeeming qualities, I, I don't know. I...

I: No, that's perfectly fine, perfectly fine. Alrighty...

P: [Inaudible] come to find out he's a, um, trainer is with the exercise group and hire him. I know I can't do that now, but I'm gonna ask Dr. Name someday, if I can do that.

I: Ok, yeah, more power to you. And so, the last...

[00:40:01.5]

P: And I know who did the [inaudible].

I: Oh, yeah. Most definitely. And so, the last question is gonna talk about the mobile phone app portion of the program. And I know you're familiar with it already, and so, could you just tell me more about your current experience with the app and what you like and do not like?

P: Um, at first, I, I get, I stay up late and um, I um, I didn't have the time set right for my sleeping, uh oh wait, I'm trying to do something, I'm sorry.

I: It's ok.

P: But I didn't have the time straight for my, you know, it has a window of time that you're supposed to sleep. So, once I learned to adjust that to more of my habits.

I: Um, ok.

P: [Just force it?] And I liked seeing my sleep time, but one of the things that kinda freaks me out is, I'm in a deep sleep more than most people. And I think, "Oh no, I hope I don't have sleep apnea." But I think sleep apnea would make you not have a deep sleep.

I: Um hm.

P: Anyway, I like seeing that, I like seeing my steps. Cause even on days, you know, cause I don't really exercise, and I don't know how they set my total steps, it's not ten thousand, which is what I know it needs to be. I think it's like seven thousand or six-fifty, six thousand five hundred, and I can probably change that, I just haven't done a lot of study on it.

I: Um hm.

P: But, um, I um, I like seeing how, like the other day I stayed up all night getting ready for company to come cause of, you know, I'm in this new place, and I just, just was in my little one bedroom condo and by noon, I had hit my goal and by the end of the day, I had walked eleven thousand steps.

I: Wow.

P: So, I like [inaudible] steps.

I: Ok.

P: Um, even though they're [inaudible] high enough and the sleep, but I, Ahad told me I didn't sync for a long time. And I, I just forgot.

I: Um hm.

P: So, I'm trying to sleep more.

I: Ok. And so, um, overall it seems like you do like the app, but there are some things that are, that might still confuse you um, are, is there anything else about the app or the Garmin device that you like or dislike?

P: Um, I've forgotten about it most of the time, I don't think it's very attractive, but I don't think any of these things are very attractive.

I: Um hm.

P: I, you know, I just...

I: In terms of how they look?

P: Yeah, yeah, but I don't like, I don't like wearing necklaces, I don't really like wearing watches, but I have liked having a watch.

I: Ok.

P: You know, because you start using your iPhone as a watch and it's not the same as wearing a watch.

I: Um hm, ok.

P: To know what time it is.

I: Um hm. And so, alright. Is there anything else um, that was the last question, was there anything else that you wanted to talk about or, about our discussion today?

P: No, I think it's been a good discussion and I appreciate, I think you have a good energy style and I hope I didn't talk too much.

I: No, not at all, not at all. We encourage you to talk more just cause it um, benefits uh, it gives us just more information to work with so, um, thank you for um...

P: I really do hope, I hope something comes from this study, long-term.

I: Yes, me too. And so, now I'm going to stop the recording.

[00:44:20.1]

Audio file name: 6-5-18PC2.mp4

Recording date : 6/5/2018

Interviewer: LLD

Place/Time: Stanford University - Santa Clara Valley

Transcriber: AB

Dates: 7/20/2018

Word File Name: CKDInterviewStanford1

* Recording begins midway through interview

P: They held my job open for 2 years, and then they offered me a job at Stanford University but I go, I can barely walk to the corner and back. I won't be able to do.. cause you know when you are a security guard, you are running, you have a radio on you, you're getting calls every 5 minutes and you have to go here and there and [Interviewer steps in]

I: So you were very active when you were working, but how about now.. now that you are not working, how, how are you physically active this week?

P: Um.. well.

I: Can you tell me a time that you were active this week? What is your exercise routine that you did this week?

P: I mop, and um. [Interviewer steps in]

I: So you did some chores.

P: Chores, yeah you know, I throw my garbage um, mostly just walking and I stop, I stop going up and down the stairs.. because of my knee.. so after that I just.. I haven't really been doing that much because my legs [emphasis], I dont know. It's my knee, and I've noticed that when I go to step up a curb, my leg really hurts you know, I don't want to end up falling down and hurting myself worse so I just like kinda take it. I've been taking it really easy. [Participant Laughs]

I: Can you tell me some benefits of exercising?

P: Um, I believe it makes you feel better... helps your heart rate, strengthens your muscles.. you, you can move quicker..

I: Any personal benefits?

P: Feel better about yourself... I think your mind is clearer...

I: Anything else that you can tell me why exercise gives you a positive benefit?

P: I don't know, I just really, you know, you know, usually you lose **Inaudible... you know.

I: What motivates you to be physically active?

P: I don't know, I want to live longer, I want to be able to do something with my grandkids. Because before, I would take them to the park and run with them and everything and now I just, you know. Now when I go see my daughter I take my walker because I figure we go somewhere but she goes no mommy, we don't need to go out **Inaudible. How come you don't want to go anywhere? And she goes, Mom, I can tell your pain. I go yeah, but I have to move [emphasis] but she just says no.. we will just stay home. See, that's kind of disappointing because you know I used to go to the park with them, she'd go jogging, and I'd go out there with the kids and whatever and now it's like, it just, when you can't move, it's like your life stops.. it's like.

I: So it's like that motivates you to be more physically active.

P: Yes.. yes.

I: Umm, how important is exercise for patients with kidney disease?

P: I think it is very important, because.. the disease robs you of a lot of strength.. but I think.. you have to keep moving because it builds up the muscles in your body. You'll be able to walk better. Because if you don't walk, you won't be able to walk pretty soon. And I don't want to get to that point, you know.. I want to be able to walk and that's what I've been able thinking, well lord if I get on dialysis, I want to still be able to walk. [Emphasis] Because you know, because I see other people who wind up in wheelchairs and stuff. But I believe it is their choice because to me, I have a high tolerance for pain and I think a lot of people they.. just not to go to the pain of moving.. they rather.. use a wheelchair or.. use one of those scooters you know. But I don't want to do that. I want to walk.

I: Has your doctor talked you about exercise, and.. kidney disease management?

P: They told me about the nutrition, but exercise... [stops sentence]

I: What has he or she told you?

P: Well I told her, you know, I was going to the **Inaudible park for a while. I was filing for somebody.. and the.. I did that for about two months. And the school year, I was active with the kids.. you know.

I: But has your doctor mentioned anything..?

P: No.. No.

I: About exercise management?

P: No..

I: So no advice from your physician..

P: (Mutters no), but she says I am doing pretty good so.. considering.. [Interviewer steps in]

0:05:00.8

I: But you mention..., you mention nutrition.. so she's.. your doctor is..

P: Yeah, I saw a nutritionist and they just gave me a paper of what I could eat.. what and everything in moderation, you know. But when I don't have a lot of food, I you know like, bread, tuna sandwiches, and I know.. that a lot of bread is not good. [Long pause] And usually when I do buy, I eat a lot of cucumbers, watermelon, zucchini..

I: How often do you see your doctor about nutrition?

P: I think I saw her once. And uh... that was like a couple years ago I guess.

I: Is it with doctor.. at here at Santa Clara Valley.

P: It was when Doctor Young was my doctor.

I: What are some of the things that make it hard for you to exercise?

P: [Thinking] I think uh.. because my vascular problem.. like when I walk on a carpeted floor, it's not so bad, but when I walk on the cement, I really feel it.

I: So it's some health barriers.. that. Any other health barriers that you.. encounter? Or how you feel unmotivated?

P: Sometimes I feel tired.. and I am not dragging.. it's just I feel tired... excuse me [Sounds like she yawned] And then, I think, so that I have my, my nights and my days.. and switch my hours, sleeping hours. Because I like the mornings, but lot of times, if I like I have a bible study late at night.. and uh.. I go to bed late so what I do. I wind up going to bed late. I wind up getting up late. So I wind staying up late and getting up late so... you know.

I: What would make it easier for you to overcome your health barriers and um.. schedule.

P: I don't know, I just want to be able to move that way at least I can go and.. have enough strength and stuff to go on the bus to buy my groceries because one of the problems, I have to go on the bus cause you gotta have a cart, you gotta fill up the cart and pull it on the bus. My other strength is not what it use to be. I use to be very strong but now you know.. it's just weakness..

I: So you wanna.. strengthen.

P: Yes.. I need to strengthen my legs and my upper body to.. to be able. [Increase volume of voice] I still do stuff for myself, but not like, you know.. before I think.. I would pick up 60 pounds with no effort but now I think that not even 20 is very hard. So I need to, I just want to strengthen my body so I can move better. [Emphasis]

I: What else would make it easier for you uh.. exercise?

P: I think being in motivation... like being with another person, together.

I: Like in a group?

P: Yes, it motivates you to move, your encouraged more, more support... (Long Pause)

I: Anything else that would make it easier to overcome those barriers?

P: **Inaudible... routine.. I would probably because I am the type of person, once I get into a routine, I like stick to it. [Laughter] I do everything the same, almost everyday. I don't know why I am like that but I do. But, I guess, that's what would be, a group, cause you know uh.. I know a lot of people, I interact with a lot of people through the church. I even counsel people. Uh, a lot of people call me, and pray for them, encourage them.. trust god, positive thinking. You know I tell them, to not accept the negativity, think positive, speak positive. I believe that is what really helps you overcome a lot of things. I believe you can resist it. Because, you know the battles in the mind. Cause a lot of people, they start thinking all this negative stuff, and there down. And then a lot of people once they allow that to come in, they can't get back up. [0:10:03.7]

And I don't believe that a person should have to take psych pills to get better. I know a lot of people at the church because we deal with a lot of people, homeless people, people that are mentally ill, and a lot of the people are taking these pills but it is not doing anything for them. It is just kind of covering the problem. They are still where they are at but uh they are like in a fog and uh..

I: So one of the things we are doing here is considering an exercise program as a group class. How do you feel about group exercise classes in general?

P: I never really uh... well when I was in school, we did exercise out there in the yard or whatever. But I've never really.. you know.. done exercises and the one time me and my sister and my neice. I don't know if you remember that guy Simon, what was his name, a little short guy...

I: Richard Simmons?

P: He had curly hair.

I: Richard Simmons.

P: Yes [Emphasis][Laughter]! One time, they bought the video Dance to the.... the oldies whenver. They were laughing and laughing it was so funny.

I: So how do you feel about these group classes?

P: You know, I don't know. I've never done it. I, I liked to try and see you know, you know. Um.. I also had a niece, she's never been very thin but she worked out at 24 hour fitness as an instuctor. And um.. I did it sometime. She came to the church and helped the ladies and did it for a while. It was okay. Cause you know.. she had a program where.. it was like... where you were really good health, and then when you were just starting, just some moves you can do to build up to what they were doing. And it was interesting. I liked it. The music was good, it was motivating. Before I was younger, I used to dance a lot.

I: In groups classes? Or group settings?

P: No, just called dancing [Laughter]

I: So how do you feel about, um... exercise created.. for people with kidney disease?

P: I think it helps you, like I said. I see a lot of people, they are in wheelchairs. Sometimes I wonder you know, maybe if, if they had help you know.. some kind of therapy or something that would help them build up their muscles, they could still walk.

I: So you feel that it's uh.. very important?

P: And i think it's.. it's.. it depends on the person. Like I said, a lot of people can't take the pain so they would rather just not even move. Just, just, you know. But not me. I'll fight until God takes me home. [Laughter]

I: Do you have any barriers.. to participating in an exercise class?

P: No.. probably just getting there. And probably just..

I: Transportation?

P: Yeah.. and the, Cause I, I don't really. You know, I'm not really doing anything, I'm free you know. I'm not in that scope program right now.

I: Any other barriers besides transportation that may... um.. be [cut off]

P: Well as far as I know, the only thing that I do is go to my doctors appointments.

I: So there could be conflicts..

P: I go to church, yeah you know. I go to church but all the things in church are on the weekends or in the evening. And doctors appointments, I usually get them in the morning. But if I the exercise program in the morning, I'll see my doctor in the afternoon.

I: In the afternoon, okay. How should we design these classes to be more appealing to you and most convenient. Is there like any.. type of activity that you enjoy..? Or.. And

P: I think the music. I think music helps people move.

I: So you would like.. um.. it to be more like.. music based..

P: Yeah.. not. I've heard something called Zumba but I don't think I could move like that. [Laughter]

I: Is there any type of activities with music that you enjoy?

0:14:59.6

P: I do reading... I read a lot. Um..

I: But as far as like physical activity? Um.. is it like um.. anything?

P: I always wanted to get me one of those big tricycles.. but I haven't because they are expensive.

I: How about location? Would it be most convenient? How should we design these courses, these classes for you.

P: Uh.. I know, I've heard of that center here. I wouldn't mind coming here if I can get here.

I: Either here or that center right...? And then that time, what would be most convenient for you?

P: Probably in the morning.

I: Okay. What would you value most about this exercise program?

P: What do you mean?

I: Um.. what would you value, like what do you want to get out of it..?

P: I just want to be strengthen, to be able to move better and uh... to be more active. I want to build up the strength of my legs, I would be all over the place on the bus. Believe me.

I: [Laughter] How would you judge if the exercise was successful?

P: By.. to be able to move.. uh better, quicker, walk longer.

I: And you find that uh.. a successful..

P: Moving helps you burning calories and you, and helps your body get smaller. [Laughter] Um.. my nephew, he was always heavy. He wasn't real real big but he was.. he was heavy and his girlfriend. And uh, when they got together, they both gained a lot of weight.. She was uh... she was a skinny little thing and before you know it, they gained a lot of weight. And then they got into uh... some program they saw on TV and it was a real uh.. how do you say uh.. advance exercise. But what my nephew did, they started going bike riding to prepare for that exercise program. And.. it was like.. his girlfriend came to see me one day and shes coming towards the door and I saw her through the window when I was staying with my friend. And I go, who's that girl? And then I saw her and I go like Oh my god! It's Maya. She look like she lost 100 pounds. Let me show you her pictures before and after. If you don't mind.

I: Oh yeah, we can do that. Let me ask you a few more questions before we um. What is your experience with exercise instructors?

P: I never had one.

I: So you never had one, okay. If you were attending an exercise class, what could the exercise instructor do to help you be more willing and excited about exercising? What can the uh..instructor do to help you?

P: I don't know maybe like, you know.. not uh... I don't know like.. I guess wouldn't embarrass me if I couldn't do something. You know. Or... just encourage me if it's something hard for me to do at first but will encourage me to keep going to.. to reach my goal. You know. Like I said, I never really you know.. been in an exercise group, or the gym or anything you know. Cause I always figured why should I pay for the gym when just walking and doing stuff. You're exercising you know. And like I said, I never went to the gym cause before I use, when I was a lot younger, I used to do a lot of dancing. But you know, as I got older, had my kids and stuff, I started gaining weight and stuff. You know.. like I said I was never really thin, but it's like.. I uh, at one time I gained a lot a lot of weight but then I lost it. And then I gained it, and then I lost it.

I: How helpful is your goal setting for you when you exercise? When you do exercise. How important is it?

0:20:02.4

P: Oh I do it.. until I get tired. Until.. you know.. I'm just like, my legs really start hurting.

I: So.. what things would discourage you? From exercising?

P: Oh like I said, mostly like uh.. like lot of times when it was raining and stuff I wouldn't go out. Like with Kidney Disease, I guess the anemia that I have, I'm always cold.

I: Okay so the weather?

P: Yeah the weather or like the other day, I wanted to go out but it was just so hot. It was just no, I'm not going out. Mostly that is what it would be you know. But otherwise.

I: Any other things that can discourage you from exercising?

P: No.. just like or if I get.. like like I said for a time, I was cramping so bad. I mean, I could just move my leg and I would just.. my calves.. were just.. I mean it was just... so excruciating it wasn't funny. And then up here, my hamstring. I was just in a lot of pain... all the time. And they offered by (narcos) but I don't believe in taking that stuff.

I: We are also considering using a mobile phone app as part of our program like I mentioned to you earlier. Can you tell me about your past or current experience about using health applications on your phone? Do you have any?

P: I have no experience.

I: Okay so no experience okay.

P: Um.. And I didn't grow up with computers, all the stuff didn't come out until I was already like.. you know, up here. [Laughter]

I: So how do you track your activity? Is it, do you track any of your, do you have a log or anything?

P: No, no.

I: Okay. And uh.. if. So if you don't have an app on your phone, have you downloaded some apps previously on your phone?

P: Yeah, I know how to do it..** Inaudible

I: How are you? Are you.. umm experienced on the computer or have any..?

P: I'm very.. I don't have wifi in my house. And uh.. I... I'm very.. not to saavy on the computer. I like about 20 years ago, I was going to a class, but I got real real sick and I just never went back. I went. I.. the cost was so far along, I just didn't go back. And that was a time, it was after 9/11. I had gotten laid off. I was uh... I was having trouble finding a job, and I was living in my car.. and uh. So I started going to this computer class. I went to a church to talk to somebody to see if I could uh.. help me get a job. And they told me about the computer pass class.. So I started going to the computer class and like two months into the class, I got real sick. I wound up in the hospital. I just didn't wind up going back. But then I went back to the same, and they had an agency there and that is when I.. got, I found out about security. I took the test to get the guard card and I started security so.. I never followed up on the computer. You know, like very basic, you know. I've thought about maybe I should go try to get into some computer class but then I go like, how would I get there. I think the main thing with me is transportation.

I: Okay, this is all the information. This is great, the way.. you've given me a lot of helpful information that.. will help us. Is there anything you want us to share with me? Or talk to me about? Or clarify to me something about it.

P: No, I'm fine. I'm not depressed or nothing. You know. Like I say, I get around you know. Like I am not sitting in my living studio. I go out. I go out with friends. I travel.

I: That's great.

P: If I can get.. if I can get along. But if you know.. a lot of people if you are disabled, really don't want to bother with you. You know what I am saying? You slow them down. Like my duaghter, she's.. 35 but she's very thin. She's always kept herself very fit. She jogs. They go, her and the girls go horseback riding. You know and whatever. And sometimes when I go down there the girls want to go have Carls Jr. [Changes voice to Imitate child?] No. I don't want no girls, nobody in my house with diabetes. [laughter] So you know, she keeps everybody fit, you know. So uh... and you know like, I went down a couple months ago and I had hurt me knee. And so, you know when I got there, she has stairs, and then she bought this truck. And oh my god, it was so high! And I told her, I don't know if I am going to be able to get in to your truck. She picked me up at the train station. And I go, she goes, why? I hurt my knee. I can't get up there. My leg is just... I told her, I told her I can walk. I'm have pain. But when I actually have to put all my weight on my leg, I can't do it. So uh.. she was like, oh well I guess were not going to do anything because you can't get on the truck and then if you can't get on the truck and then.. She has, lives in a two story house and going up and down those stairs you know, holding on to the banister.. helped me.

0:26:16.2

End of Interview

Audio File Name: 6-22-18DA.mp4

Recording Date: 6/22/2018

Interviewer: LLD

Transcriber: AB

Dates: 7/23/2018, 7/24/2018

File Name: #2 CKD Interview Stanford (6-22-2018)

0:00:00.0

I: Date of Visit June 22nd 2018. Study ID: 43198-016

**START OF INTERVIEW

I: I want to learn a little bit about you. So uh, if you can just tell me about a time when you physically active that you really enjoyed? Can you tell me about a time that you were physically active that you really enjoyed.

P: Physically active.. Probably when I was in my thirties.. so.. I would exercise uh probably 3 or 4 times a week. I mean you know, that's when I.. I guess I didn't let myself go.

I: What kind of exercises that.. that you enjoyed?

P: Oh, it was yoga, for a while so. But, most of them too are muscle building.

I: That would also...

P: Yeah muscle building. We would actually do some hiking. Um...

I: Would you do that additionally then? With the yoga? You would do that on the weekends?

P: Yeah.. on the weekends yeah.

I: Okay. So..

P: **Inaudible [Long Pause, Participant starts crying] I guess when my sister passed away..

I: I'm sorry...

P: Maybe, it just.. seems... [deep breath]

I: When did she pass away? I'm sorry...

P: [Clears throat] I think 2007 [or 2002]

I: And did she do the exercises with you?

P: Uhh no.. but.. after she passed away, it just.. sort of... downhill. And everything.. Like nothing mattered or.. I mean, everything... [cough] Life goes on so... I guess after that, it's just... not worth uh doing something or you know. Maybe because I, umm we were there to take care of her kids too so uhh and stuff.

I: Oh yeah.. so that really affected..

P: The lifestyle just changed and stuff so.. it felt like somebody is missing..

I: Yeah... I'm sorry.. Let me get some napkins... Kleenex. I will be right back. [Leaves room]

0:04:39.3

**Returns to room

0:05:00.2

P: Thank you. I didn't realize **inaudible When you asked me about it... I guess, it just.. That was in the back of mind and stuff. Maybe it dawned on me. Those were the days.

I: Those were the days you were physically active, yeah.

P: Physically active, I guess happy.. and you know. So.

I: What do you think about when you hear about the word exercise? Can you give me some thoughts about how you feel when you exercise?

P: [Deep Breath] It's like uh, I want to do it. But because of uh.. how I've become, I mean sometimes its uh.. I have a hard time moving. [Clears Throat] That um.. I'd like to do it but... sometimes it's. Maybe because when I had that, my tire [or tired?].. it just gave me.. um not enough um... not enough strength. I was like I am tired every time and so... But, I, something that I wanna do, is exercise. I wanna do it but.. Every time I do it, I think I every time I did it, my bones were (activate)

I: So it's painful.

P: And stuff, I'd have to wait again and stuff. That, that, that kills me and so... It's just like, I'm there, I'm going to do it again and stuff. So hopefully that doesn't happen anymore. But what I did was I bought, this sit cycle [laughter] and I haven't been doing it for a while so..

I: Okay so, what are your current exercise routine right now?

P: Nothing right now.. [laughter] but I try to do that uh.. sit cycle, for at least 15 minute or 30 minute. Sometimes I sit down.

I: So it's a sit cycle, you do it for 30...

P: 15-30 minutes

I: 15-30 minutes.. and do you do it um.. how long.. like every day? Or.. a couple times a week?

P: Probably a couple times a week.

I: Like 2 or 3?

P: 2.

I: 2 times a week okay. And where do you do this sit cycle at? Do you do it..

P: At home. Yeah, it's in my room. So.. [mumbling] your watching TV.

I: You do it in your home, in your room while you are watching TV. Okay.. So is this your usual amount of physical activity?

P: I think it was a problem that I had before with... I didn't know. My doctor said that because you sweat, and because I am diabetic, I am prone to UTI. And sometimes, my UTI would kick in. So it hurts my.. [inaudible]. Those were some of the problems I have too.

I: Oh.. with the.. with the sweat.

P: That's what she said. Because every time I try.. doing uh.. sort of extensive.. more than uh, heavy exercises.. that's when it kicks in.

I: Oh I see. Okay. So... so this is your normal usual workout is.. the sit cycle. Um.. can you describe a week when you were active? Like what would you do? Like, when you were active, what were.. your exercise routines?

P: Well uh.. in the evening we'd go to YMCA. Uh.. probably uh.. MWF [ Monday Wednesday Friday] or twice a week. And then. And the weekends, we'd go hiking by [Alum] Rock.

I: And how often is the YMCA? Like.. you would do that?

P: Weekly..

I: Weekly, okay. And for how, was it, how long, like 1 hour or..

P: We were there for probably an hour or two. Because we go in a group and stuff so.

I: How were you physically active this week?

P: Uh.. not so much because of my.. so I just walk.

I: So you walk. Where do you walk?

P: Oh just walking, the office and stuff.

0:10:00.4

I: Oh, okay so you walk...

P: I, I, there is a fairgrounds so sometimes we would walk around the fairgrounds.

I: How many times this week did you exercise or.. when you were walking?

P: I couldn't do my bicycle because of my... so. Walking would be probably once a week. It's hard to.

I: So how did you exercise change when you were diagnosed chronic kidney disease?

P: Uh, well it didn't really change that much.

I: It didn't change, okay.

P: I was still.. Because when I started that chronic... [long pause] I guess after when I was diagnosed with diabetes, that's when I, it slowly..

I: That was um, 5.. 5 years ago right?

P: Yeah:

I: But when, so you, was your exercise the same when you were diagnosed with kidney disease?

P: Mhm, I would yeah.

I: It was the same.

P: It was the same.

I: So you still do the walking?

P: I still do the walking.

I: And the.. okay. [pause] So.. what are some of the benefits of exercise for you?

P: Oh. I lose a lot of weight. Mmmm..

I: Any health..?

P: My strength.

I: Your strength.

P: And now, you know it's hard to move. I'm not building that much muscle so..

I: Any personal benefits?

P: [laughter] personal benefit? Self esteem. It's, it's, [shut]. Self esteem is not that good. So.. That's one of the non benefits. Uh... Confidence... [Long pause] You look good.

I: So what motivates [cut off]

P: You.. you enjoy trying out clothes. [laughter] Now a days.. ooh no, no, no. [Emphasis] But life goes on.

I: What motivates you to be.. physically active? What motivates you?

P: My grand nephew. That.. He loves going to park so I go with him to the park. I'm just enjoying him.

I: So how important is exercise?

P: Very important. But uh, it's something that I really have to work on and stuff. That's why.. maybe this could help. [laughter]

I: Has your doctor talked to you about exercise?

P: Yes he did

I: And uh... kidney disease management? So.. has your doctor talked to you about this? What has she told you?

P: Oh right now?

I: Yeah, what were her recommendations? Or any advice that she was giving you?

P: Like regarding exercise?

I: Yeah.

P: Like just the normal.. exercise and stuff.

I: Like, how many times? Did she like say.. that it is good to exercise once a week? or twice a week?

P: Well, she didn't touch on exercise yet so... when we met again. But Dr.[Name?], yeah, definitely wanted me to.. you know.

I: Start exercising?

P: And lose weight.

I: So she's been monitoring that and giving you advice. Yeah.. with your current nephrology, nephrologist Dr. [Name?] You haven't met, have you met with her?

P: We met already but uh... she wasn't more on the exercise yet and stuff so. Because we were just starting and stuff.

I: Yeah.. okay. So what are some of the things that make it hard for you to exercise? Can you tell me that?

0:14:58.9

P: Cause at the end of the day, I am so tired, that.. I just want to rest. Put up my feet and stuff. On the weekends, I.. work.

I: Oh so you work on the weekends too?

P: Yeah. And I just taking care of.. everybody at home. My sister, my mom.

I: So as far as health barriers, does that make you.. hard for you to exercise? Any health barriers? Or is it time? Is it time that makes you...

P: Time. Yeah. Time and uh... I think just more time.

I: More tired.

P: It's just I need to rest first but the time I get rest... [laughter] so many... oh time to sleep. [ laughter]

I: Do you feel unmotivated at times?

P: Probably.

I: What would make it easier? For you to overcome these barriers?

P: **Deep breath. I don't know. That's what I am trying to figure out. That's why any chance that I get that, you know when you told me about this study. Maybe I can be motivated with this and stuff. I mean, anything.. [Long pause, flipping of pages]

I: So one of the consideration, were considering in this study is group classes. How do you feel about group exercise in general?

P: It's okay with me. Sometimes maybe that will give motivation. If there is a group.

I: So you like um, you like group classes.

P: Yeah.

I: How do you feel about exercise classes that are created for people with kidney disease?

P: Oh, I want to try it. I mean, if it's something that will help me prevent.. the progression of the disease, why not.

I: So.. uh any barriers to participating.. in these classes?

P: No. The only thing is uh.. how I can travel to that place?

I: How about transportation and time?

P: Well if I am, I drive so that's fine. I'm fine with that. With right now.

I: What is it that you? Oh, so you broke

P: I broke my, my foot. The foot not the ankle. So.. if that's fix by. I mean if this is fixed by the time we start, I have no problem. I can go any time.

I: So.. okay how should we design these classes to be most convenient for you? And more appealing to you? [laughter]

P: More food [laughter] Uh, as long as uh, it's not really very hard, I think, I can, I can. I mean it is something that I uh, but if it's gonna ... um. What is it called this... uh. Start making me feel so.. so pumped up that I can't breath anymore.. I quit. Because I have asthma too so. But, it's not that. I've got the pump and stuff. I don't want that to be a deterrent but. Uh, it's something that, you know I.. I've done exercises before.. without the help.

I: So location.. So is there specific types of activities that..

P: Well anything that I would probably be able to handle. Not..

I: Like for instance, like what kind of exercise is that, that you would be interested in.

P: Maybe uh, muscle building.

I: Okay, something with weights.

P: Something with weights. Walking. Um... those, those climbing stuff, that's not.

I: So like a stair, stair master.

0:19:59.6

P: Eliptic [Elliptical]

I: Eliptic, okay.

P: I can do that stuff. I mean, on the lower pace. I've done that with um... when I was young. [laughter] and vibrant.

I: You still are! [Laughter] How about location and time? What would be most convenient with you?

P: I think if it's near my uh.. residence. No problem. So if the location is probably.. The most probable would be uh... 30 miles away.

I: How about time? Time of the day? What is the most appealing to you? Most convenient?

P: Probably in the afternoon.

I: Afternoon. So after work or?

P: After work.

I: So like times after 5?

P: After 5. At least I don't have to worry about going back to work. [Laughter]

I: What would you value most about an exercise program?

P: What would I value. Well, uh the results. Uh.. I mean, I am happy with my [fif teen years?] If I lose weight, Oh! That would be great.

I: Anything else? That might be of value for this..?

P: If it could give me confidence. Yeah.

I: How would you judge if the exercise was successful?

P: Well if I lose weight. [laughter] All the results I got. All of the.. I mean that will give me self esteem.

I: So personal success, so if you lose...

P: weight

I: And if you feel good about yourself. That will determine your, the success of the program. Okay um.

P: My objective is actually, was to, make better of myself. [pause]

I: Okay. What is your experience with exercise instructors?

P: Um...I think we had one only once. Yes. I have no problem with that. ** Inaudible. Give me more... added tips. They'll be good. I guess if I am doing it wrong, I wanna know. [Laughter]

I: Yes. Yes. Yeah so they give you some advice right?

P: Like, am I doing it right? So..

I: So.. if you were attending an exercise class, the exercise, what could the exercise instructor do to help you be more willing to exercise? And excited about exercise? What would the instructor, what would you like the instructor to do?

P: Well more, probably be upbeat.. Um I like, feel that everybody is connected and stuff. Uh... what do you call this? I mean how would you call it... More excitement, in the instructions... Probably with a sense of humor. At least it is not boring.

I: What would discourage you from exercising?

P: Uh.. well if the instructor is no good. Well if you are not connecting with them. Uh.. and if it's so hard for me..

I: Like if it's.. like?

P: Too strenuous.

I: Strenuous. Okay.

0:24:57.8

I: Anything else that would discourage you from exercising?

P: [long pause] Well... I'd say uh.. maybe if the instructor is boring. [laughter] And if it's uh... what do you call it. The way she's doing it, the routines are not.. it's so hard, we can't catch up. He's just focused on the group. I'd say I quit.

I: That's when you say..

P: [laughter] If he just focuses on one group, and the rest is, is not. I would be frustrated with that.

I: So you, you would like the instructor to be more connected with the whole entire group. Yeah, not just with certain people.

P: Yeah.

I: So we are considering using a mobile phone app as part of our program. Can you tell me about your current or past experiences using a health app. Do you have one on your phone?

P: I think I do. The, I think the one that's walking.

I: So, so you have exercise apps?

P: The iRunner.

I: Okay, the runner. Okay. Well do you like it? Or do you not like it? Give me thoughts about it.

P: Well... I haven't been using it [laughter] I think it's okay. But, uh it's something that you, you record how many calories and stuff you know. I am fine with it. That's one of the motivations too like you know. Oh, 337 calories! That's good! It tells you the distance.

I: So is there anything you don't like about that app?

P: Uh... I haven't tried it really. I used this for walking. I think uh, I mean...

I: So you are still using that app when you walk?

P: Yeah..

I: So you are good... [laughter. Countdown in background 3.. 2...1 I think the participant shows the interviewer the app] So you are currently using the app, you are familiar with the app, you have no problem using technology

P: No, no, no, no.

I: Okay, well that's good! Great. This is great information. Is there anything you want to share with us? Or anything that you want some clarification.

P: Uh.. nothing.

I: Okay I am going to end this.

**END OF INTERVIEW

P: Audio File Name: 6-22-17 ah.mp4

Recording Date: 6/22/2018

Interviewer: LLD

Transcriber: AB

Dates: 7/30/2018

File Name: #3 CKD Interview Stanford (6-22-2018)

*Start of Interview

I: Study ID Number 43198-11- Interview Date June 22nd, 2018

[Pre-Interview Discussion]

**[0:01:18.9]**

I: So, I wanted to ask you a few questions. Can you tell me time you were physically active that you really enjoyed?

P: A time that I was physically active that... well to be honest with you, up until I was probably uh... [long pause] the time I was about 34-35, I was actually very physically active. I played a lot of softball, played a lot of basketball, then I tore my Achilles tendon playing softball.. in the mid-eighties, which is like my early, my mid-thirties. And after I tore my Achilles, I did that, really kind of put a damper on my physical activity. Um, I still um had a pretty heavy, up until about um.. I'd say four years ago. I had a pretty heavy regimen of walking. I used to walk probably uh... 3 or 4 miles a day. Uh, at least. Before that even more because I live for a long time, I live in Evergreen Foothills. I don't know if you know Evergreen at all but uh.. you know where Clyne Park is? You know where Ruby and Tully is? Up in the foothills there?

I: Yeah

P: I lived really on the corner of Ruby and Tully, basically. So, I would go up Tully [Morreal?] which is what the street is. Go up Clyne, and then go up in the park there, prospect park, and walk most of that park. And that's a pretty good walk because it is all uphill. Even when you get in Prospect park, there is a lot of up hills. So, I was pretty heavy on that regimen until.. I moved from there in 2012 and then moved where I lived now, which is pretty flat, I would walk from where I live now which is uh... up to you know I live near [St. Clair?] University. So, I would walk from where I live now up to [St. Clair] train station and back on a daily basis. And that is probably, two miles um. And that really in some ways what, I don't know if you know all this history but.. What happened was, in 2015 I started to have shortness of breath right? All of sudden, where I could just walk. No problem, I could that walk in a New York minute, right? And I can power walking with no problem. I started to have real problems making that. I mean, that's where I first noticed I was having some real problems. Because all of sudden, something that was pretty easy to do was probably 15-20-minute walk became an hour-long exercise. And a lot of times, I would have to stop.. and just rest. And um, it was in you know, that I really realized my blood pressure got really out of control. I came in here in January of 2016. My blood pressure 242/70 something. I mean it was crazy. I was hospitalized for about a week. **[0:05:01.2]** And um, they did a bunch of stuff to try to work with me, to get my blood pressure controlled. At that [Emphasis] that all of these issues I am dealing with now. The heart failure, the kidney problems, all those were kind of discovered during that period of time. That's really where I began, when I first started seeing doctors [Sussana, Nathan] in the patch clinic during that period of time. So yeah, I mean I was pretty active. To answer your question, I was pretty active. And even now, I try to.. and even before you called me in the study I um.. been trying to be more consistent about my exercise regimen. Getting up in the morning, doing 50 crunches, doing a modified pushup. Doing you know some stretches. Because when you get older, you got to stretch your muscles in your body, to keep your elasticity in your body. So I've been doing these stretches. So, it's been really timely that you called me about this exercise program, this exercise program.

I: So what do you think when you hear the word exercise? Give me some of your thoughts.

P: Well you know, myself, I've been about... as I said to you earlier, I've been involved in youth athletics now for... this year my 29th year in football and 29th year in basketball as well. Obviously one of the things I appreciate of the kids is every good athlete is well conditioned. So.. I teach kids that in order to be a well-conditioned athlete [static, interruption in video recording- 0:06:41.8] ... discipline, exercise regimen. So, I wasn't practicing what I was preaching [laughter] Uh so, obvious step in exercise is it is an important endeavor. Like a lot of people um.. as you get older you always find excuses. Well I'll do it later or I'll do it tomorrow. So, I'd be lying if I said I didn't fall into that. So I'm hopeful that what will happen out of this study is that I'll learn some things about how to exercise, how to do you know exercises that will generate some health improvements and then after it is over, I will continue to do those exercises. That is one of the one of the reasons why I want to be a part of it, because I'm hopeful that from the discipline associated with doing this study, I'll be able to just use that as a jump.. a jumping off point for you know, healthy regimens.

I: So, tell me about your current exercise routine right now?

P: Uh.. it consists of basically of walking and as I said, doing some lower intensity crunches and doing some modified pushups. I mean I am on my knees and really just pushing up.. of my knees. And walking. That is basically my exercise regimen. During the football season, I'm out on the field. You know, I'm the president of the program so I don't, I'm not um, I guess I do some coaching, but I am not one of the active coaches. I am just the president. So, I but I am there and I'm doing different stretches for the kids and that's how I am active. I am walking the football field. We have what's called a Gator, that I try to avoid riding in it. When I try to go someplace I try to walk. Just as a way.. to get more exercise. We live in a two-story home so I'm always obviously I'm going up and down the stairs.. **Inaudible And that was one of the other things that let me know that I was having some issues because going up and down the stairs every day, which was something that I was doing pretty regularly with no problem, all of a sudden became uh.. again, you know I had to.

I: Did you get shortness of breath?

P: Well it was just tough to do right? It was because my blood pressure was so high. It was just really tough to do. Um.. I began to get heavy duty swelling in my legs, and in my ankles and in my calves blew up, you know. And I first, I just thought kind of the swelling you get from being sedentary, but you know, it became much more than that. And so, I realized um.. it was a real issue.

I: So, um, your exercise, walking, crunches, pushups, is that, how often do you do this. How frequent?

P: Well every day.

I: Every day for how long?

P: Um.. when I walk, I try to walk long enough to work up a sweat. I don't always get it done **[0:09:59.1].** Because unless you are really power walking.. it is hard to really work up a sweat walking if you just basically strolling.

I: So how.. do you walk 30 minutes to an hour?

P: I try to go about 20 minutes, 20-25 minutes.

I: And then your crunches, and that's at home?

P: I do 50, you know I do 50 repetitions of 10. I do a repetition of 10. I take 10 seconds off and I do 10 more. Over the course of time, I do 50. And then I do essentially the same thing with sit-ups, I do modified sit-ups right. Excuse me, modified pushups. I do 10 count to 10, then do 10, then do 10 more. Do it in a... I'll do 5 repetitions of that, so I do 50.

I: So, you do this all at home? And the walking at home, in your neighborhood?

P: At home.. yea at home. Yes.

I: Yeah. Okay how did you exercise routine change when you were diagnosed with kidney disease?

P: Um... [Long pause] I think it, you know, I think at first it was less. It became.. I became more inconsistent. You know.. because I was concerned about what really what.. I could do. So, I became inconsistent. Before that I was pretty consistent. Every morning, I got up at least a set everyone morning. What I would do is, I would walk on Monday, Wednesday, and Friday. I would you know, I would walk consistently 3 days a week. Or at least 20, typically 25 minutes. However long it took me to make that circuit. Um.. me.. my wife and I sometimes late in the evening, we tend to be night owls. So late in the evening, we would do a circuit together. Do a circuit around the block on those off days. I think.. when I was diagnosed with the kidney disease, I became less consistently. I guess that is the easiest way to sum it up. The discipline associated with my exercise got [way]...

I: What are some of your thoughts on how exercise benefits you?

P: Well, there is no doubt that it gets my heart rate up which is.. good. Uh, because obviously it helps my heart. Um.. you know the thing about exercise is.. you know. I don't know that you lose weight exercising, but it helps you maybe maintain weight. Because you know in order to lose weight, you have to do some really high intensity exercises on a really discipline sort of basis. And so... I wasn't doing that. So I was just using.. more I thought it was maintaining. I'm hopeful that I will get to the point where I will be able to do more weightlifting. Because my understanding is that when you get older, weightlifting helps. One, it helps with your bones. It keeps your ones strong. Your muscular strong. And also, it gets your heart pumping too as well. And.. it will burn fat.

I: So those are your, those are the benefits that..

P: That's what I see as a benefit, yes.

I: Any personal um.. benefits or.

P: In terms of?

I: Exercise?

P: For mental health practicing you mean? Um... man I assume there are some, I mean from a intellectual perspective I assume that there are some.. some mental health benefits that come from being... that you know, slows you down a little bit. I mean one of the things I use.. I use to use my walking time.. for prayer. Um.. I use to but I decided.. for some reason I thought that that was just a little bit... how would say it.. um I don't want to say sacrilegious, that's not the word I want to say. Um, but I felt that I did, that walking sometimes when you are trying to pray, that walking is a distraction. Because there are things going around you that you got to keep your eyes open. You got to look where you are going.

I: Yeah, be more aware of your surroundings.

P: I felt like it was a distraction to prayer and so I stopped praying when I was walking and prayed before I walked. And then go walk. Or pray after I come back from walking. Um.. but yeah I.. I you know, there is no doubt that you feel, if you have a good.. **[0:14:59.5]** brisk walk you come back and you feel heightened energy.

I: So more energy as far as when you do more exercise.

P: Yeah, you definitely feel more energetic right? You know.

I: What motivates you to be physically active?

P: I want to lose some weight [emphasis]. I mean, I know that losing weight is.. um for me it's an important element. It is a tough thing to do. But more importantly, it's an important element. I mean, I would like to have a little more flexibility. And I believe that if I lost weight, I would be more flexible. Um.. you know. Because as you know getting up and down in a chair.. you know just moving around. A lot of times around the football field, I try to demonstrate different things to kids if I had more.. If I lost weight I would have better flexibility, that would.. bring me some mental health benefits. [laughter] You know, just to be able to. Until about.. a month ago, I had a SL500. Which is like a 2 seater, Mercedes 2 seater right. Because it is so low to the ground, you know. I had it for about 6 years. It was starting to become a little of a journey to get in and out of it. Ultimately, I sold it and got something else. Um.. I mean all those things, all of those like losing weight would be for me.. one of the things I would hope exercise would start me on that journey. You know, anybody that is doing it, and I'm sure you do, doing anything about losing weight is really, your diet is probably more important than the exercise. What you eat is probably more important than the exercise part of it. Just because of the level of intensity that you would have to exercise at... in order to really burn.. burn away some serious calories. But I know.. I would not mind um.. as I said. I have come to understand that weightlifting is a way of.. not power lifting but just weight lifting, lifting weights, is a way of maybe burning some of that, getting to that level of intensity where you can begin. **Inaudible... Because you create more muscle and muscle helps your metabolism. So yeah.

I: How important is exercise uh for patients with Kidney Disease?

P: I think it is. I don't know how important it is for patients with kidney disease, but I do think it is important for me.

I: Why is that?

P: I think it can be a key for me, being more discipline and having much more disciplined approach to it. Um... could be a key for me to um.. I mean one of the other things that I like to do, which is related to weight, is I would like to take less pills.

I: So less medication

P: Until.. two years ago, when I had an issue where they had to hospitalize me for... I never took any pills. I mean, and so now you know I'm like a walking pharmacy. Um.. so, I you know, if I could lift. But I've been told that losing weight will help me reduce the amount of medication that I have to take. And I would like to reduce the amount of medication I have to take?

I: Has your doctor talked to you about exercise and kidney disease management?

P: I mean.. every doctor. I shouldn't say every doctor. Doctor...*Inaudible [naming doctors] ... They all said hey look, if you lost some weight, that would... I mean they always [motivated] to exercise consistently. To have a consistent plan for exercise. And they also said, if you lose some weight, that would also... When I was complained to them about the amount of medication I was taking, they said hey look, one of the ways to reduce the amount of medication you take is for you to lose some weight. Um...

**[0:20:00.3]**

I: Any exercise advice that you were given from any of your doctors?

P: Uh.. no not really. No not really. No. But my wife is.. um a person who is really much into um.. finding ways to improve the quality of life so she is constantly looking for things, constant research and things. Um... saying here are some things you should look at, you should read, you should be aware of, exercise related, that may help you in terms of you doing exercise.

I: What are some of the things that make it hard for you to exercise?

P: Um.. things that make it hard for me to exercise... I don't know. It's just a conundrum because in some ways, it is relatively, it should be relatively easy for me to exercise because I pretty much have control over my whole day and I haven't had it in a long time. So, if I decide I want to spend an hour of the day exercising, then there is nobody around to stand there.. hey look. I pretty much set my own hours. I set my start time. I set my ending time. I set the times that I do things during the course of the day. Um.. but there are marks of.. from my.. just you know.. I am working. I am doing research for the job. I am involved in these two programs. Um.. I do stuff with my church. Uh.. so I got a lot of stuff going right. I don't watch too much tv, too much football, too much basketball or whatever the sport might be. And so, I would say, if I wanted to boil it down to like two words, it would be time management. I need to do a better job of managing my time.. and to make sure.. and another word I would use is discipline. To have the discipline to essentially treat exercise like it was you know, one of the more important tasks that I have to do each day. I mean, you know exercise is one of those things that you say wow, if I have time then I will do it. If I don't have time, I will put it off. And I think.. obviously, that is not the proper attitude.

I: Do you have any health barriers to exercise? Is that one of the reasons?

P: Health Barriers?

I: Or any...

P: Things that keep me from exercising? Things that make it difficult for me to exercise? Other than what I spoke to you about my shoulder, no not really. But you know..

I: What would make it easier for you to overcome these barriers?

P: I just need to have more discipline. I mean, just being more um.. disciplined. I mean I.. somebody would say if you.. you know some people might say look at me, a group of people that you did something with, like having a partner, somebody to exercise with that would hold you accountable might be a methodology for.. improving your exercise discipline. And that there is some truth to that. Um... but I'm for the most part, I am a pretty self-directed person. And if it is something I am really passionate about that I really want to do, I do it. Uh and you don't really have to.. you know.. you know when I am really passionate about doing something, people don't have to motivate me from the outside. So.. I just have to learn how to be more passionate, more discipline about exercise.

I: So one of the things we are doing in this study in the exercise program is a group class. How do you feel about group classes in general?

P: I'm alright. You know I'm alright. You know.. I'm... because I do a lot of volunteer work, I work a lot with.. you know in the community. With all different types of people. Some motivated, some not so motivated. And so that aspect of it is.. it would not. If the purpose of the questions, **[0:25:00.3]** would I be shy or would I be at ease around other people as a result of.. my weight or you know physical condition. Um.. the answer to that question is no. If I thought that work with that group is the best thing for me, I would do it. I would not, I wouldn't have any self-esteem issues that would stand in the way of me working with other people.

I: Do you have any dislikes or.. likes about exercise groups study? Like.

P: Likes or dislikes?

I: No, dislikes.. Like you told me..

P: Oh dislikes? In terms of types of exercises?

I: Yeah group exercises.

P: Types of group exercises?

I: Yeah, is there anything you don't like about that?

P: Uh... no I mean. I haven't because most of the exercises that I have done... that were what I would call just pure exercise. Growing up playing basketball, growing up playing baseball, that's exercise too. But that is not what I call pure exercise right. That's not exercise for exercise purposes. That is more like going out, playing a game, just shoot it up, and just all of that. You are getting exercise by participating in that event. Um.. but from a pure exercise perspective, I really never been in a group where our basic focus was exercising together. You know, obviously I played a lot of basketball. Pickup basketball. Not obviously, but I have played a lot of basketball. Um.. football and baseball and.

I: So, you haven't had an exercise group class?

P: I never had an exercise, a class where I just went to exercise.

I: How do you feel about exercise class for people who have kidney disease?

P: That what?

I: That have kidney disease.

P: I mean.. my hope is.. because I have never done it. I don't know that I have a feeling about it but my hope is.. that um, I would learn some things that would help me um.. have more fun you know.. exercising. So that you know, probably what happens with exercising I think, is that if it becomes a chore, you know and once it becomes a chore, once it becomes something.. in a lot of ways like prayer. Once it becomes something that you are obligated to do as oppose to something you are doing because you love God. It's a little bit of a chore, and you kind of like ahhhh, you look for ways to avoid it. When it is a chore, you look for ways to avoid it. So I would like to learn to um.. do some types of exercises that um.. are interesting, you know maybe fun. Um.. you know things that stimulate my intellect as much as my body hopefully. If that is possible.

I: So, do you have any barriers in participating in group exercise? Like transportation or time?

P: Oh, those type of barriers. No.. I mean time like I said is um... I will say because I am a real estate agent, although I have control of my time, in some ways that you know, my time is controlled by my clients. And so.. the times when I may have something planned like exercise and the client calls me and says I need for you to come do this, or we need to go see this, or we need to go do that. And sometimes, and obviously because I am a working person like everybody who is making a living, I sometimes have to give in to that. Like okay, if it is better for you to do this at this time, whatever I am doing, I will figure out how to do it at a different time. So that would be my only barrier.

I: With your work schedule.

P: It would be a time management [issue].

I: How should we design these classes that is most convenient and most appealing to you?

P: Most convenient to me?

I: Is there like type of activities that you enjoy better than others? Or...

P: Um.. nah I really like walking, I like that.. I do not.. I am not very big of doing stuff on treadmills **[0:30:00.9]** and bikes and that kind of exercise. I am not a very big fan of that. Particularly treadmills and bikes. I mean, I would rather walk or ride a bike then to get on a bike and just ride. You know.. do a stationary exercise on a bike or a stationary exercise on a treadmill. Even when you got a TV screen. Oh dear, I would much prefer to do something where I am kind of out in the fresh air. Or semi fresh air.

I: So, like, like outdoors. So those are more appealing to you then doing stationary bikes.

P: I also would probably prefer if I was going to do some exercise, I would prefer.. if I had a preference. If somebody said, hey look, here is a game you can learn to play that will get you the same appropriate amount of exercise [that is going in..] **Inaudible I would probably prefer that right. My nature... **Inaudible... would prefer that.

I: How about location and time? What um..

P: You know.. I mean.. for me any place.. obviously, location is based on the time of day. If I can do between 10 and 1 o'clock, (I... more is that?) Because I get around the area pretty good, I mean traffic wise. If it's got to be done before 10 or after 1, then I obviously closer to home would better because...uh you know, before 10 or after 1, traffic in this bad boy is horrible. I mean it use to be 3 o'clock now traffic starts getting bad.. I don't want to.. (go there?) [laughter] But I mean it does, now I-17 is jammed at 1 o'clock in afternoon. 17 is jammed at 1 o'clock. uh.. so.

I: So... what would you value most from an exercise program?

P: Uh... I mean I like to be active. I am not by.. [long pause] At least I imagine myself to like to be active. [Laughter] That's a strange thing to say but people say things all the time but you always are. I imagine myself, myself liking to be active. I certainly like to be around people. I like to interact with people. Right? I love to be active. I think of myself as.. I have this image in my head and I am aware of myself as an athlete. So because of that aspect of it, um.. It causes me to want to be active. Um..

I: How would you...?

P: I tend to be...

I: I'm sorry, go ahead, I interrupted you.

P: I tend to have more energy.. I tend to have more energy.. it's a strange thing. I tend to have more energy in the afternoon then in the morning. But, I tend to be a pretty early riser. Most of the time, when I get up in the morning, I don't want to do... I just want to get up. I don't necessarily want to do anything, but I just want to get.. up. And be awake in the morning. And you know.. I probably, my prime time for activity is probably between about 11 and.. for physical activity my prime time is probably between about 11-2. Mentally, it is probably between 11 and two in the morning. But physically, it is probably between 11-2 in the afternoon. I guess that's how..

I: So how would you judge if the exercise program was successful?

P: How would I judge it's success... [whisper] Um... you know if.. my judgement would be.. are my health vitals you know? I go to the doctor and they say your vitals, your pulse **[0:34:59.8]**, your breathing seems in good shape you know. If I had energy. If I had increased energy. That would an indicator that the fact that exercise is being successful. Um.. if I lost weight.. I guess. You know.. I mean it would be beautiful if I lost 50 pounds but just being realistic, if I lost 5 or 10 pounds, that would be. I would deem the exercise to be successful.

I: So.. what is your experience with exercise instructors?

P: Well, I've never really had a... at least not since grade school. [laughter] PE Teacher.

I: If you were attending an exercise class, what would you want the instructor to help you be more willing and excited about exercising? What could the instructor do?

P: I would want the instructor to.. teach me how to do exercises that I can do.. I would want the instructor I could do in close quarters. Something I could in my bedroom. Something I could alone in the living room. Something.. you know not, something necessarily.. I am not that big on.. gyms. I know that going to gyms is the new, new thing. Everybody wants to go to the gym, but I am not that big on the gyms.

I: So, something that you could do at home?

P: Yeah, I would prefer to do have exercises that I could at home as oppose to sitting and going to the gym and do that. But I wouldn't be opposed. Like somebody said, hey look you know, I would recommend that you go to the gym to do these types of exercises. It would beneficial to you. I wouldn't run away from that. I wouldn't be opposed to it. But that wouldn't be my first choice. My first choice would to do some things that I could at home. Just because I believe if I had some things I could at home, I would more likely do them. Because, if I could get out of bed or what have you and just go do some exercises as opposed to have to get up, get dressed, and drive to the gym.

I: So, you want the instructor to teach you more techniques that you can duplicate at home?

P: Yeah.

I: Are there things that would discourage you from exercising?

P: You know, I am a habitual person, but I am not a routine person if that makes any sense. I mean.. I form habits really easy but I am not personally... I am not a person that's giving in to a lot of routine, a lot of routine things. So.. if I don't know how to explain.. how can I explain this? If it is something I like doing and I think it is good for me. If I can gracefully fall into the habit of doing it, and I will be disciplined about it. If it is something that is routine and kind of boring, you know and I am doing it dispassionately, you know that I won't.. I may not be as disciplined about that.

I: I see. Got it. So um.. we are considering using a mobile app as part of our program. So are you currently using any.. do you have.. tell me if you have used any health apps on your phone?

P: I haven't used any help.. help apps. The one app that I am starting to use really has nothing to do with my health. It has to do with the health of my.. kids in my program. Um.. we have this app that we are using now called HitCheck. It has to do with concussion safety. And um.. so, kids do a baseline test and for some reason if they have a concussion, which we have rarely I should say. If they have a concussion, and they come back and take the test again and if they don't reach that baseline, it indicates **[0:39:59.6]**, if they are off that baseline, they are not allowed to participate till they get back to the baseline.

I: Okay this app is for like...

P: I'll queue it up on my phone right here.

I: What is it called?

P: Hitcheck.

I: But it's not.. it's more for your team.

P: It's more for my players. It's not for me personally. It's more for more players. I must say, I want once took the baseline and I was having trouble with it.

I: Do you have any activity tracker on your phone for you?

P: Uh.. the Fitbit is. Oh yeah the Fitbit is on my phone too as well.

I: So you.. you were using that. Did you like it or dislike it? What are your thoughts on that?

P: Well to be honest with you.. I kind of liked.. there are aspects of it that I did like but there are aspects of it that were like. Some of it was kind of like.. you need.. I think for those things to really work out well. I understand, I don't have one. But my son tells me that the apple watch is much more intuitive. So you can figure how it works pretty quickly. The Fitbit was a little bit techy.

I: It was too techy? Or it was not straight to the point?

P: Yeah, it was a little bit techy in terms of figuring out you know.. how to use it effectively right? Like the steps. You know.. the reality is that almost anytime you made a motion, it recorded it as a step. So, I had like thousands of steps on my phone.

I: So it was too.. it was too much to like..

P: It was too technical in my opinion. It was you know..

I: And you want it a little bit more simple.

P: For it to be more effective tool, it would nice if you could figure out a way where you don't have to think about it. Because once you have to start thinking about stuff on how to do it right, it is just one more thing you have to do. Whereas where if it was more... also more intuitive in that sense um.. I could probably better explain it with cars, you familiar with cars?

I: A little bit.

P: All of the Lexus cars, all of the Japanese cars are very intuitive. All of that navigational stuff, you get in. You don't have to be no technical genius. You pretty much can figure it out. You do it a couple times. And you can pretty much work it through. But all of the German cars, the BMW's, the Mercedes, there all.. you almost have to be a technical genius to figure out their systems. And that's kind of how I thought about Fitbit. You kind of had to be kind of a technical genius to really kind of figure out all of its systems. So.. you don't use it right? Because all that stuff. When I say it is intuitive, something is kind of logical right? This should be kind of the next logical step and yet, you got this you know. There are all the funny things you have to do. Like the Mercedes you know, when you push the button to put the seat, the memory seats in. Most cars you push the button right to the memory seat. But the Mercedes, you got to hold your finger until the thing gets into place.

I: So with the Fitbit, was that one of the reasons why you stopped?

P: Yeah, yeah, the Fitbit was kind of like you know. I was saying well.. okay, I really want to exercise. How do I get the thing to tell me.. um you know if you wear it all day. You wear it all the time. So, you get up in the morning right, and it's supposed to tell you if you got good sleep or not right? That's one of the things that uh.. that for me was.. important because although I tend not to sleep.. Sleep is the healing process that most people don't know. And for me unfortunately, I tend to sleep deeply but not necessarily.. with longevity. I mean I can sleep 4 hours and feel totally energized. Whereas.. the Fitbit is supposed to help you with all that. And trying to uh.. you know, figure that out. Figure out what time you go to sleep and just. Some of the um.. let's see. I was going to use the word (massenations?) but some of the way the thing works were... it should've. I would have hoped that it would have been more intuitive. Easily figured out. Easily.. solved for a lack of a better word.

I: Okay.. So, I think you provided a lot of information to me...

**End of Interview

P: Audio File Name: 6-22-17 ah.mp4

Recording Date: 6/22/2018

Interviewer: LLD

Transcriber: AB

Dates: 7/30/2018

File Name: #3 CKD Interview Stanford (6-22-2018)

*Start of Interview

I: Study ID Number 43198-11- Interview Date June 22nd, 2018

[Pre-Interview Discussion]

**[0:01:18.9]**

I: So, I wanted to ask you a few questions. Can you tell me time you were physically active that you really enjoyed?

P: A time that I was physically active that... well to be honest with you, up until I was probably uh... [long pause] the time I was about 34-35, I was actually very physically active. I played a lot of softball, played a lot of basketball, then I tore my Achilles tendon playing softball.. in the mid-eighties, which is like my early, my mid-thirties. And after I tore my Achilles, I did that, really kind of put a damper on my physical activity. Um, I still um had a pretty heavy, up until about um.. I'd say four years ago. I had a pretty heavy regimen of walking. I used to walk probably uh... 3 or 4 miles a day. Uh, at least. Before that even more because I live for a long time, I live in Evergreen Foothills. I don't know if you know Evergreen at all but uh.. you know where Clyne Park is? You know where Ruby and Tully is? Up in the foothills there?

I: Yeah

P: I lived really on the corner of Ruby and Tully, basically. So, I would go up Tully [Morreal?] which is what the street is. Go up Clyne, and then go up in the park there, prospect park, and walk most of that park. And that's a pretty good walk because it is all uphill. Even when you get in Prospect park, there is a lot of up hills. So, I was pretty heavy on that regimen until.. I moved from there in 2012 and then moved where I lived now, which is pretty flat, I would walk from where I live now which is uh... up to you know I live near [St. Clair?] University. So, I would walk from where I live now up to [St. Clair] train station and back on a daily basis. And that is probably, two miles um. And that really in some ways what, I don't know if you know all this history but.. What happened was, in 2015 I started to have shortness of breath right? All of sudden, where I could just walk. No problem, I could that walk in a New York minute, right? And I can power walking with no problem. I started to have real problems making that. I mean, that's where I first noticed I was having some real problems. Because all of sudden, something that was pretty easy to do was probably 15-20-minute walk became an hour-long exercise. And a lot of times, I would have to stop.. and just rest. And um, it was in you know, that I really realized my blood pressure got really out of control. I came in here in January of 2016. My blood pressure 242/70 something. I mean it was crazy. I was hospitalized for about a week. **[0:05:01.2]** And um, they did a bunch of stuff to try to work with me, to get my blood pressure controlled. At that [Emphasis] that all of these issues I am dealing with now. The heart failure, the kidney problems, all those were kind of discovered during that period of time. That's really where I began, when I first started seeing doctors [Sussana, Nathan] in the patch clinic during that period of time. So yeah, I mean I was pretty active. To answer your question, I was pretty active. And even now, I try to.. and even before you called me in the study I um.. been trying to be more consistent about my exercise regimen. Getting up in the morning, doing 50 crunches, doing a modified pushup. Doing you know some stretches. Because when you get older, you got to stretch your muscles in your body, to keep your elasticity in your body. So I've been doing these stretches. So, it's been really timely that you called me about this exercise program, this exercise program.

I: So what do you think when you hear the word exercise? Give me some of your thoughts.

P: Well you know, myself, I've been about... as I said to you earlier, I've been involved in youth athletics now for... this year my 29th year in football and 29th year in basketball as well. Obviously one of the things I appreciate of the kids is every good athlete is well conditioned. So.. I teach kids that in order to be a well-conditioned athlete [static, interruption in video recording- 0:06:41.8] ... discipline, exercise regimen. So, I wasn't practicing what I was preaching [laughter] Uh so, obvious step in exercise is it is an important endeavor. Like a lot of people um.. as you get older you always find excuses. Well I'll do it later or I'll do it tomorrow. So, I'd be lying if I said I didn't fall into that. So I'm hopeful that what will happen out of this study is that I'll learn some things about how to exercise, how to do you know exercises that will generate some health improvements and then after it is over, I will continue to do those exercises. That is one of the one of the reasons why I want to be a part of it, because I'm hopeful that from the discipline associated with doing this study, I'll be able to just use that as a jump.. a jumping off point for you know, healthy regimens.

I: So, tell me about your current exercise routine right now?

P: Uh.. it consists of basically of walking and as I said, doing some lower intensity crunches and doing some modified pushups. I mean I am on my knees and really just pushing up.. of my knees. And walking. That is basically my exercise regimen. During the football season, I'm out on the field. You know, I'm the president of the program so I don't, I'm not um, I guess I do some coaching, but I am not one of the active coaches. I am just the president. So, I but I am there and I'm doing different stretches for the kids and that's how I am active. I am walking the football field. We have what's called a Gator, that I try to avoid riding in it. When I try to go someplace I try to walk. Just as a way.. to get more exercise. We live in a two-story home so I'm always obviously I'm going up and down the stairs.. **Inaudible And that was one of the other things that let me know that I was having some issues because going up and down the stairs every day, which was something that I was doing pretty regularly with no problem, all of a sudden became uh.. again, you know I had to.

I: Did you get shortness of breath?

P: Well it was just tough to do right? It was because my blood pressure was so high. It was just really tough to do. Um.. I began to get heavy duty swelling in my legs, and in my ankles and in my calves blew up, you know. And I first, I just thought kind of the swelling you get from being sedentary, but you know, it became much more than that. And so, I realized um.. it was a real issue.

I: So, um, your exercise, walking, crunches, pushups, is that, how often do you do this. How frequent?

P: Well every day.

I: Every day for how long?

P: Um.. when I walk, I try to walk long enough to work up a sweat. I don't always get it done **[0:09:59.1].** Because unless you are really power walking.. it is hard to really work up a sweat walking if you just basically strolling.

I: So how.. do you walk 30 minutes to an hour?

P: I try to go about 20 minutes, 20-25 minutes.

I: And then your crunches, and that's at home?

P: I do 50, you know I do 50 repetitions of 10. I do a repetition of 10. I take 10 seconds off and I do 10 more. Over the course of time, I do 50. And then I do essentially the same thing with sit-ups, I do modified sit-ups right. Excuse me, modified pushups. I do 10 count to 10, then do 10, then do 10 more. Do it in a... I'll do 5 repetitions of that, so I do 50.

I: So, you do this all at home? And the walking at home, in your neighborhood?

P: At home.. yea at home. Yes.

I: Yeah. Okay how did you exercise routine change when you were diagnosed with kidney disease?

P: Um... [Long pause] I think it, you know, I think at first it was less. It became.. I became more inconsistent. You know.. because I was concerned about what really what.. I could do. So, I became inconsistent. Before that I was pretty consistent. Every morning, I got up at least a set everyone morning. What I would do is, I would walk on Monday, Wednesday, and Friday. I would you know, I would walk consistently 3 days a week. Or at least 20, typically 25 minutes. However long it took me to make that circuit. Um.. me.. my wife and I sometimes late in the evening, we tend to be night owls. So late in the evening, we would do a circuit together. Do a circuit around the block on those off days. I think.. when I was diagnosed with the kidney disease, I became less consistently. I guess that is the easiest way to sum it up. The discipline associated with my exercise got [way]...

I: What are some of your thoughts on how exercise benefits you?

P: Well, there is no doubt that it gets my heart rate up which is.. good. Uh, because obviously it helps my heart. Um.. you know the thing about exercise is.. you know. I don't know that you lose weight exercising, but it helps you maybe maintain weight. Because you know in order to lose weight, you have to do some really high intensity exercises on a really discipline sort of basis. And so... I wasn't doing that. So I was just using.. more I thought it was maintaining. I'm hopeful that I will get to the point where I will be able to do more weightlifting. Because my understanding is that when you get older, weightlifting helps. One, it helps with your bones. It keeps your ones strong. Your muscular strong. And also, it gets your heart pumping too as well. And.. it will burn fat.

I: So those are your, those are the benefits that..

P: That's what I see as a benefit, yes.

I: Any personal um.. benefits or.

P: In terms of?

I: Exercise?

P: For mental health practicing you mean? Um... man I assume there are some, I mean from a intellectual perspective I assume that there are some.. some mental health benefits that come from being... that you know, slows you down a little bit. I mean one of the things I use.. I use to use my walking time.. for prayer. Um.. I use to but I decided.. for some reason I thought that that was just a little bit... how would say it.. um I don't want to say sacrilegious, that's not the word I want to say. Um, but I felt that I did, that walking sometimes when you are trying to pray, that walking is a distraction. Because there are things going around you that you got to keep your eyes open. You got to look where you are going.

I: Yeah, be more aware of your surroundings.

P: I felt like it was a distraction to prayer and so I stopped praying when I was walking and prayed before I walked. And then go walk. Or pray after I come back from walking. Um.. but yeah I.. I you know, there is no doubt that you feel, if you have a good.. **[0:14:59.5]** brisk walk you come back and you feel heightened energy.

I: So more energy as far as when you do more exercise.

P: Yeah, you definitely feel more energetic right? You know.

I: What motivates you to be physically active?

P: I want to lose some weight [emphasis]. I mean, I know that losing weight is.. um for me it's an important element. It is a tough thing to do. But more importantly, it's an important element. I mean, I would like to have a little more flexibility. And I believe that if I lost weight, I would be more flexible. Um.. you know. Because as you know getting up and down in a chair.. you know just moving around. A lot of times around the football field, I try to demonstrate different things to kids if I had more.. If I lost weight I would have better flexibility, that would.. bring me some mental health benefits. [laughter] You know, just to be able to. Until about.. a month ago, I had a SL500. Which is like a 2 seater, Mercedes 2 seater right. Because it is so low to the ground, you know. I had it for about 6 years. It was starting to become a little of a journey to get in and out of it. Ultimately, I sold it and got something else. Um.. I mean all those things, all of those like losing weight would be for me.. one of the things I would hope exercise would start me on that journey. You know, anybody that is doing it, and I'm sure you do, doing anything about losing weight is really, your diet is probably more important than the exercise. What you eat is probably more important than the exercise part of it. Just because of the level of intensity that you would have to exercise at... in order to really burn.. burn away some serious calories. But I know.. I would not mind um.. as I said. I have come to understand that weightlifting is a way of.. not power lifting but just weight lifting, lifting weights, is a way of maybe burning some of that, getting to that level of intensity where you can begin. **Inaudible... Because you create more muscle and muscle helps your metabolism. So yeah.

I: How important is exercise uh for patients with Kidney Disease?

P: I think it is. I don't know how important it is for patients with kidney disease, but I do think it is important for me.

I: Why is that?

P: I think it can be a key for me, being more discipline and having much more disciplined approach to it. Um... could be a key for me to um.. I mean one of the other things that I like to do, which is related to weight, is I would like to take less pills.

I: So less medication

P: Until.. two years ago, when I had an issue where they had to hospitalize me for... I never took any pills. I mean, and so now you know I'm like a walking pharmacy. Um.. so, I you know, if I could lift. But I've been told that losing weight will help me reduce the amount of medication that I have to take. And I would like to reduce the amount of medication I have to take?

I: Has your doctor talked to you about exercise and kidney disease management?

P: I mean.. every doctor. I shouldn't say every doctor. Doctor...*Inaudible [naming doctors] ... They all said hey look, if you lost some weight, that would... I mean they always [motivated] to exercise consistently. To have a consistent plan for exercise. And they also said, if you lose some weight, that would also... When I was complained to them about the amount of medication I was taking, they said hey look, one of the ways to reduce the amount of medication you take is for you to lose some weight. Um...

**[0:20:00.3]**

I: Any exercise advice that you were given from any of your doctors?

P: Uh.. no not really. No not really. No. But my wife is.. um a person who is really much into um.. finding ways to improve the quality of life so she is constantly looking for things, constant research and things. Um... saying here are some things you should look at, you should read, you should be aware of, exercise related, that may help you in terms of you doing exercise.

I: What are some of the things that make it hard for you to exercise?

P: Um.. things that make it hard for me to exercise... I don't know. It's just a conundrum because in some ways, it is relatively, it should be relatively easy for me to exercise because I pretty much have control over my whole day and I haven't had it in a long time. So, if I decide I want to spend an hour of the day exercising, then there is nobody around to stand there.. hey look. I pretty much set my own hours. I set my start time. I set my ending time. I set the times that I do things during the course of the day. Um.. but there are marks of.. from my.. just you know.. I am working. I am doing research for the job. I am involved in these two programs. Um.. I do stuff with my church. Uh.. so I got a lot of stuff going right. I don't watch too much tv, too much football, too much basketball or whatever the sport might be. And so, I would say, if I wanted to boil it down to like two words, it would be time management. I need to do a better job of managing my time.. and to make sure.. and another word I would use is discipline. To have the discipline to essentially treat exercise like it was you know, one of the more important tasks that I have to do each day. I mean, you know exercise is one of those things that you say wow, if I have time then I will do it. If I don't have time, I will put it off. And I think.. obviously, that is not the proper attitude.

I: Do you have any health barriers to exercise? Is that one of the reasons?

P: Health Barriers?

I: Or any...

P: Things that keep me from exercising? Things that make it difficult for me to exercise? Other than what I spoke to you about my shoulder, no not really. But you know..

I: What would make it easier for you to overcome these barriers?

P: I just need to have more discipline. I mean, just being more um.. disciplined. I mean I.. somebody would say if you.. you know some people might say look at me, a group of people that you did something with, like having a partner, somebody to exercise with that would hold you accountable might be a methodology for.. improving your exercise discipline. And that there is some truth to that. Um... but I'm for the most part, I am a pretty self-directed person. And if it is something I am really passionate about that I really want to do, I do it. Uh and you don't really have to.. you know.. you know when I am really passionate about doing something, people don't have to motivate me from the outside. So.. I just have to learn how to be more passionate, more discipline about exercise.

I: So one of the things we are doing in this study in the exercise program is a group class. How do you feel about group classes in general?

P: I'm alright. You know I'm alright. You know.. I'm... because I do a lot of volunteer work, I work a lot with.. you know in the community. With all different types of people. Some motivated, some not so motivated. And so that aspect of it is.. it would not. If the purpose of the questions, **[0:25:00.3]** would I be shy or would I be at ease around other people as a result of.. my weight or you know physical condition. Um.. the answer to that question is no. If I thought that work with that group is the best thing for me, I would do it. I would not, I wouldn't have any self-esteem issues that would stand in the way of me working with other people.

I: Do you have any dislikes or.. likes about exercise groups study? Like.

P: Likes or dislikes?

I: No, dislikes.. Like you told me..

P: Oh dislikes? In terms of types of exercises?

I: Yeah group exercises.

P: Types of group exercises?

I: Yeah, is there anything you don't like about that?

P: Uh... no I mean. I haven't because most of the exercises that I have done... that were what I would call just pure exercise. Growing up playing basketball, growing up playing baseball, that's exercise too. But that is not what I call pure exercise right. That's not exercise for exercise purposes. That is more like going out, playing a game, just shoot it up, and just all of that. You are getting exercise by participating in that event. Um.. but from a pure exercise perspective, I really never been in a group where our basic focus was exercising together. You know, obviously I played a lot of basketball. Pickup basketball. Not obviously, but I have played a lot of basketball. Um.. football and baseball and.

I: So, you haven't had an exercise group class?

P: I never had an exercise, a class where I just went to exercise.

I: How do you feel about exercise class for people who have kidney disease?

P: That what?

I: That have kidney disease.

P: I mean.. my hope is.. because I have never done it. I don't know that I have a feeling about it but my hope is.. that um, I would learn some things that would help me um.. have more fun you know.. exercising. So that you know, probably what happens with exercising I think, is that if it becomes a chore, you know and once it becomes a chore, once it becomes something.. in a lot of ways like prayer. Once it becomes something that you are obligated to do as oppose to something you are doing because you love God. It's a little bit of a chore, and you kind of like ahhhh, you look for ways to avoid it. When it is a chore, you look for ways to avoid it. So I would like to learn to um.. do some types of exercises that um.. are interesting, you know maybe fun. Um.. you know things that stimulate my intellect as much as my body hopefully. If that is possible.

I: So, do you have any barriers in participating in group exercise? Like transportation or time?

P: Oh, those type of barriers. No.. I mean time like I said is um... I will say because I am a real estate agent, although I have control of my time, in some ways that you know, my time is controlled by my clients. And so.. the times when I may have something planned like exercise and the client calls me and says I need for you to come do this, or we need to go see this, or we need to go do that. And sometimes, and obviously because I am a working person like everybody who is making a living, I sometimes have to give in to that. Like okay, if it is better for you to do this at this time, whatever I am doing, I will figure out how to do it at a different time. So that would be my only barrier.

I: With your work schedule.

P: It would be a time management [issue].

I: How should we design these classes that is most convenient and most appealing to you?

P: Most convenient to me?

I: Is there like type of activities that you enjoy better than others? Or...

P: Um.. nah I really like walking, I like that.. I do not.. I am not very big of doing stuff on treadmills **[0:30:00.9]** and bikes and that kind of exercise. I am not a very big fan of that. Particularly treadmills and bikes. I mean, I would rather walk or ride a bike then to get on a bike and just ride. You know.. do a stationary exercise on a bike or a stationary exercise on a treadmill. Even when you got a TV screen. Oh dear, I would much prefer to do something where I am kind of out in the fresh air. Or semi fresh air.

I: So, like, like outdoors. So those are more appealing to you then doing stationary bikes.

P: I also would probably prefer if I was going to do some exercise, I would prefer.. if I had a preference. If somebody said, hey look, here is a game you can learn to play that will get you the same appropriate amount of exercise [that is going in..] **Inaudible I would probably prefer that right. My nature... **Inaudible... would prefer that.

I: How about location and time? What um..

P: You know.. I mean.. for me any place.. obviously, location is based on the time of day. If I can do between 10 and 1 o'clock, (I... more is that?) Because I get around the area pretty good, I mean traffic wise. If it's got to be done before 10 or after 1, then I obviously closer to home would better because...uh you know, before 10 or after 1, traffic in this bad boy is horrible. I mean it use to be 3 o'clock now traffic starts getting bad.. I don't want to.. (go there?) [laughter] But I mean it does, now I-17 is jammed at 1 o'clock in afternoon. 17 is jammed at 1 o'clock. uh.. so.

I: So... what would you value most from an exercise program?

P: Uh... I mean I like to be active. I am not by.. [long pause] At least I imagine myself to like to be active. [Laughter] That's a strange thing to say but people say things all the time but you always are. I imagine myself, myself liking to be active. I certainly like to be around people. I like to interact with people. Right? I love to be active. I think of myself as.. I have this image in my head and I am aware of myself as an athlete. So because of that aspect of it, um.. It causes me to want to be active. Um..

I: How would you...?

P: I tend to be...

I: I'm sorry, go ahead, I interrupted you.

P: I tend to have more energy.. I tend to have more energy.. it's a strange thing. I tend to have more energy in the afternoon then in the morning. But, I tend to be a pretty early riser. Most of the time, when I get up in the morning, I don't want to do... I just want to get up. I don't necessarily want to do anything, but I just want to get.. up. And be awake in the morning. And you know.. I probably, my prime time for activity is probably between about 11 and.. for physical activity my prime time is probably between about 11-2. Mentally, it is probably between 11 and two in the morning. But physically, it is probably between 11-2 in the afternoon. I guess that's how..

I: So how would you judge if the exercise program was successful?

P: How would I judge it's success... [whisper] Um... you know if.. my judgement would be.. are my health vitals you know? I go to the doctor and they say your vitals, your pulse **[0:34:59.8]**, your breathing seems in good shape you know. If I had energy. If I had increased energy. That would an indicator that the fact that exercise is being successful. Um.. if I lost weight.. I guess. You know.. I mean it would be beautiful if I lost 50 pounds but just being realistic, if I lost 5 or 10 pounds, that would be. I would deem the exercise to be successful.

I: So.. what is your experience with exercise instructors?

P: Well, I've never really had a... at least not since grade school. [laughter] PE Teacher.

I: If you were attending an exercise class, what would you want the instructor to help you be more willing and excited about exercising? What could the instructor do?

P: I would want the instructor to.. teach me how to do exercises that I can do.. I would want the instructor I could do in close quarters. Something I could in my bedroom. Something I could alone in the living room. Something.. you know not, something necessarily.. I am not that big on.. gyms. I know that going to gyms is the new, new thing. Everybody wants to go to the gym, but I am not that big on the gyms.

I: So, something that you could do at home?

P: Yeah, I would prefer to do have exercises that I could at home as oppose to sitting and going to the gym and do that. But I wouldn't be opposed. Like somebody said, hey look you know, I would recommend that you go to the gym to do these types of exercises. It would beneficial to you. I wouldn't run away from that. I wouldn't be opposed to it. But that wouldn't be my first choice. My first choice would to do some things that I could at home. Just because I believe if I had some things I could at home, I would more likely do them. Because, if I could get out of bed or what have you and just go do some exercises as opposed to have to get up, get dressed, and drive to the gym.

I: So, you want the instructor to teach you more techniques that you can duplicate at home?

P: Yeah.

I: Are there things that would discourage you from exercising?

P: You know, I am a habitual person, but I am not a routine person if that makes any sense. I mean.. I form habits really easy but I am not personally... I am not a person that's giving in to a lot of routine, a lot of routine things. So.. if I don't know how to explain.. how can I explain this? If it is something I like doing and I think it is good for me. If I can gracefully fall into the habit of doing it, and I will be disciplined about it. If it is something that is routine and kind of boring, you know and I am doing it dispassionately, you know that I won't.. I may not be as disciplined about that.

I: I see. Got it. So um.. we are considering using a mobile app as part of our program. So are you currently using any.. do you have.. tell me if you have used any health apps on your phone?

P: I haven't used any help.. help apps. The one app that I am starting to use really has nothing to do with my health. It has to do with the health of my.. kids in my program. Um.. we have this app that we are using now called HitCheck. It has to do with concussion safety. And um.. so, kids do a baseline test and for some reason if they have a concussion, which we have rarely I should say. If they have a concussion, and they come back and take the test again and if they don't reach that baseline, it indicates **[0:39:59.6]**, if they are off that baseline, they are not allowed to participate till they get back to the baseline.

I: Okay this app is for like...

P: I'll queue it up on my phone right here.

I: What is it called?

P: Hitcheck.

I: But it's not.. it's more for your team.

P: It's more for my players. It's not for me personally. It's more for more players. I must say, I want once took the baseline and I was having trouble with it.

I: Do you have any activity tracker on your phone for you?

P: Uh.. the Fitbit is. Oh yeah the Fitbit is on my phone too as well.

I: So you.. you were using that. Did you like it or dislike it? What are your thoughts on that?

P: Well to be honest with you.. I kind of liked.. there are aspects of it that I did like but there are aspects of it that were like. Some of it was kind of like.. you need.. I think for those things to really work out well. I understand, I don't have one. But my son tells me that the apple watch is much more intuitive. So you can figure how it works pretty quickly. The Fitbit was a little bit techy.

I: It was too techy? Or it was not straight to the point?

P: Yeah, it was a little bit techy in terms of figuring out you know.. how to use it effectively right? Like the steps. You know.. the reality is that almost anytime you made a motion, it recorded it as a step. So, I had like thousands of steps on my phone.

I: So it was too.. it was too much to like..

P: It was too technical in my opinion. It was you know..

I: And you want it a little bit more simple.

P: For it to be more effective tool, it would nice if you could figure out a way where you don't have to think about it. Because once you have to start thinking about stuff on how to do it right, it is just one more thing you have to do. Whereas where if it was more... also more intuitive in that sense um.. I could probably better explain it with cars, you familiar with cars?

I: A little bit.

P: All of the Lexus cars, all of the Japanese cars are very intuitive. All of that navigational stuff, you get in. You don't have to be no technical genius. You pretty much can figure it out. You do it a couple times. And you can pretty much work it through. But all of the German cars, the BMW's, the Mercedes, there all.. you almost have to be a technical genius to figure out their systems. And that's kind of how I thought about Fitbit. You kind of had to be kind of a technical genius to really kind of figure out all of its systems. So.. you don't use it right? Because all that stuff. When I say it is intuitive, something is kind of logical right? This should be kind of the next logical step and yet, you got this you know. There are all the funny things you have to do. Like the Mercedes you know, when you push the button to put the seat, the memory seats in. Most cars you push the button right to the memory seat. But the Mercedes, you got to hold your finger until the thing gets into place.

I: So with the Fitbit, was that one of the reasons why you stopped?

P: Yeah, yeah, the Fitbit was kind of like you know. I was saying well.. okay, I really want to exercise. How do I get the thing to tell me.. um you know if you wear it all day. You wear it all the time. So, you get up in the morning right, and it's supposed to tell you if you got good sleep or not right? That's one of the things that uh.. that for me was.. important because although I tend not to sleep.. Sleep is the healing process that most people don't know. And for me unfortunately, I tend to sleep deeply but not necessarily.. with longevity. I mean I can sleep 4 hours and feel totally energized. Whereas.. the Fitbit is supposed to help you with all that. And trying to uh.. you know, figure that out. Figure out what time you go to sleep and just. Some of the um.. let's see. I was going to use the word (massenations?) but some of the way the thing works were... it should've. I would have hoped that it would have been more intuitive. Easily figured out. Easily.. solved for a lack of a better word.

I: Okay.. So, I think you provided a lot of information to me...

**End of Interview

Audio File Name: 7-24-18_43198-32.mp4

Recording Date: 7/24/2018

Interviewer: LLD

Transcriber: AB

Dates: 8/4/2018

File Name: #5 CKD Interview Stanford (7-24-2018)

**Start of Interview

Study ID: 43298-32

I: Okay (Name), can you tell me about a time that you were physically active that you really enjoyed?

P: It's long time ago. Like, 2-3 years ago. Yeah.

I: What were the activities that you enjoyed?

P: Exercise and then doing bike. Walking.

I: What do you think when you hear the word exercise?

P: I am happy.

I: You're happy when exercise comes into your mind? You think of happiness?

P: Yeah. It makes me happy.

I: So, can you tell about your current exercise routine right now?

P: Right now, I just walk for 45 minutes, half an hour. Not long. But you know, I live on 3rd floor. No elevator. Oh, sometimes I am going up and down two three times. The stairs. Like 4 four stairs.

I: So, do you walk every day for 30-45 minutes?

P: Not really. Sometimes because I have knee surgery, I have a lot of pain. I couldn't walk.

I: Okay. And mainly, you walk around the house?

P: Yes.

I: Okay. Is that your usual amount of activity before your knee surgery?

P: No. Before I was doing exercise. Walking a lot like one hour. [cough]

I: So... did you exercise routine change when you were diagnosed with kidney disease?

P: Yeah, because I have been very sick. I have had a lot of pain. For 4 months, I couldn't move. I couldn't eat. I was just lying down on the couch. I don't like to eat anything. Yeah, then my son, he said mom, but you going to eat. I bring you food, ask for food. I was like... throw up. Yeah, my feeling was very bad.

I: Oh.. So, it changed.

P: Yeah, you know when you are sick? Like sometimes I am sick, but I am okay, let me get up. I don't want to lay down. But that, that was painful. I couldn't go up. I was scared because I was dizzy and then very weak. That's why, just I stay home. I.. was scared to go up. Maybe I fall down, or something happen to me.

I: So, can you tell me some benefits of exercising, for you? What are some benefits of exercising?

P: Make me healthy and you know. Like, I feel like I am doing something for myself.

I: Any other benefits?

P: I don't know.

I: What motivates you to be physically active?

P: What does that mean?

I: So, why do you want to be healthy and active? What motivates you?

P: Because, I don't want to like, lay down.. or sit at home. Lay down on couch, watch TV. No. I want to be strong for myself. To go up, to do something.. independent. **[0:04:57.9]** Because all my life, I was independent you know? Just, sometimes I ask my kids, can you do that for me? Otherwise, I did everything. Yeah.

I: So, how important is exercise for patients with kidney disease?

P: I think it is very important.

I: Has your doctor talked to you about exercise? And, any kidney disease management?

P: No.

I: No? She had no exercise advice that she has given to you?

P: No.

I: What are some of the things that make it hard for you to exercise?

P: Like, you know treatment. I couldn't go because my knee, it hurts. If I want to go on a treadmill, you know it is fast. I am scared maybe that I couldn't control. That's something happened.

I: Oh, okay. Anything else besides your knee pain? Is there anything else that makes it hard for you to exercise?

P: No.

I: What will make it easier to overcome the knee pain for you to exercise?

P: I don't know.

I: Stretching?

P: Yes, stretching. I cannot like stretch all the way. Or, uh do it like that. It hurts. Yeah. I have to keep it in [middle?]. You know?

I: Oh okay. So, um one of the things were considering with the study is group exercises. How do you feel about group exercises?

P: I like it.

I: You like it exercises. Um, so you like exercising with people?

P: Mhm.

I: Um. How do you feel about exercise classes, especially created for patients with kidney disease?

P: I didn't try it before but if it's necessary, I am going to try. [Cough] Sorry.

I: Um, is there any barriers for you to participate? Is there anything that will stop you from participating in this exercise study?

P: I don't understand what that means.

I: Are there any things that will stop you from being in the study?

P: No.

I: No? Like for instance, like time or transportation to get here. Is that something that...?

P: I don't think so. Just maybe when I am very sick.

I: But as far as any things, factors that will make you not come to these exercise classes. You have a car and your transportation is not an issue?

P: No.. no.

I: How would we design these classes to be more appealing to you? What would you like these classes to have so you will enjoy it? Are there specific activities that you like? What kind?

P: I don't know really. I didn't been in these classes. I don't know what they have. But they, sometimes I see people like doing like kind of like dance.

I: Dance, uh huh.

P: Yeah! Or..

I: The stretches?

P: Stretches or things. When I start, then I am going to look which one...

I: Which.. which you are interested in?

P: Yes.

I: How about location and time? Most of these classes are here. Um, nearby. Um so, do you live near this area?

P: No, I am not living in this area but it's okay. I can come over here. Exactly where is it located?

I: Well, were still trying to [figure it out]. I think it is here at the Tiffany Center, just down the street from here at the corner. So, it is just at the corner here on [Bascum?] **[0:10:00.0]**

P: Okay.

I: Yeah. so it's not too far.

P: Okay. It's okay.

I: How would you know if an exercise program is successful?

P: I think exercise is successful for people to do things, you know? Make them to move. Make it like talk other people. Maybe make you more happy. Because there is some people. You talk to them and share things with them. Make a difference.

I: Yeah, yeah, so that determines if this exercise program can be successful. If you have these.

P: Yeah!

I: Um, what is your experience in exercise instructors? Have you had any experience with any exercise instructors?

P: Uh.. you know like I told you, I did by myself, exercise. Not with others.

I: Okay, so you did a lot of exercises on your own.

P: Yes. That time I was doing, I don't have any pain anything. I do on treadmill or bike. Biking or um, we had like a pool over there. I walked in the pool. I liked that.

I: Oh okay.

P: Yeah.

I: So, if you were attending the exercise class, what could the instructor do to help you be excited about exercising? What can the instructor do? What would you like them to do?

P: Right now, really, I don't know. But when I see or start, maybe. I see what they doing. I do too.

I: Okay, so you kind of like to, to um copy their routines or..

P: Yeah.

I: Are there any things that would discourage you from exercising? What would make you stop exercising?

P: Maybe pain.

I: Okay.. anything else?

P: No.

I: So, we are considering using a mobile phone app as part of the program. Can you tell me, in the past, have you used any apps on your phone?

P: No. Just for emergency. I have the... yeah. If something happened, call my kids or. I am not home. They call me to find out where I am.

I: So, but you don't have any health apps? Like, anything you track your health. Your walking?

P: No.

I: Okay. Are you um, would you be willing to do that if we offered that to you? With.. the exercise study? Would you be willing to learn about these applications on your phone to help track your exercise?

P: Okay.

I: Yeah?

P: Yeah.

I: Wel okay, this is great.

**[0:13:43.6]**

** End of Interview

Audio File Name: 7-25-18_43198-12.mp4

Recording Date: 7/25/2018

Interviewer: LLD

Transcriber: AB

Dates: 8/4/2018

File Name: #6 CKD Interview Stanford (7-25-2018)

**Start of the Interview

43198-012

Date of Visit: July 25th, 2018

I: I have to do backup. [laughter] Okay. So [Name], can you tell me about a time you were physically active that you really enjoyed?

P: A time in my life?

I: Yes, that you really enjoyed exercising.

P: Oh, I use to be uh, I use to be a runner. But with neuropathy, it is very difficult to run nowadays. Because you can't keep your balance. Um.. I used to play lots of sports, bowling and uh basketball, and football. So that was probably in my twenties. And I use to do jazzercise.

I: Fun!

P: But that was to pick up girls. [laughter]

I: Yeah. So that was uh, those were times back in your twenties that you were physically active?

P: Yeah 20's, early 30's. Yup. I mean more active than ever.

I: So, what do you think when you first hear the words exercise? What are your thoughts?

P: Um, you know getting in shape. Um, doing a different type of diet also. Um, and it's my condition not so hard to do. In other words, it is not a lot of running involved but more walking. You know, trying to set a better pace.

I: Okay.. So, tell me about your current exercise routine right now?

P: Basically, right now, just walking the dog half a mile a day, mile a day. Twice a day.

I: Twice a day. So where do you walk?

P: Just around our block. But we have a block where there is a.. probably a severe incline um.. that is like walking up a little mountain.

I: So, it is an incline hill a little bit.

P: Right. Yeah.

I: So how long do you do this? Like every day? Every other day?

P: Well recently, again. Back to doing it again, every day. But you know, I use to.. a year ago, it would be twice a day. Without you know, take the dog for a walk.

I: So, is this your usual amount of physical activity?

P: Yeah, pretty much.

I: Yeah. So how did your exercise routine change when you were diagnosed with kidney disease?

P: Well, for me, it is more neuropathy is the problem than it is the kidney disease. You know um... you know neuropathy, your balance is off, and.. when your endurance is off. So.. that is what changed it. You can't walk further. You can't go on. It can get frustrating that you don’t.. you know.

I: Mhm. So, what are some of the benefits of exercising?

P: Um, being health, of course. And, I mean you know, having uh, having again endurance to keep going on. Sleeping better.

I: Any other health or personal benefits that exercise..?

P: Being happier. For sure.

I: Yeah.

P: That's.. I don't know any more adjectives.

I: What motivates you to be physically active?

P: Um.. what motivates you.. Um, knowing that you are going to live longer [laughter] You know, if you are just going to lay around all day, other things are going to happen to you. Medically so. [0:05:00.9]

I: [pause] So, how important is exercise for patients with kidney disease?

P: Oh.. very important. You're.. As I said, if you just lay around, other things will happen. Bad things will happen to you. You will have pneumonia. You will get fluid in your lungs. Um, and depression will set in. If you don't exercise.

I: So, has your doctor talked to about exercise and any other kidney disease management?

P: No.. The things about it, for exercising, you can't.. your chances of getting a new kidney diminish because if you are not fit, they won't accept you in their programs. I don't know if you know that. So, that is one of the first things they ask. Can you walk up a certain flight of stairs? So, you know, if you don't meet those criteria, then your chance of getting a kidney is diminished. Where, that's why it is important that you exercise.. to try to reach their level.

I: So did you doctor..?

P: Well, she explained that to me. Which is what your question was. So yeah, she talked to me about it. Which is why we are sitting here, is because... [laughter]. She is the one that recommended me to you, so.

I: So, what kind of exercise advice were you given from your doctor? Or, if any?

P: Yeah.. not really advice as much as don't give up. She said, we'll find a way to help you.

I: What are some of the things that make it hard for you to exercise?

P: Oh, just neuropathy.

I: So like..

P: My feet. Unbalanced. Because the exercise that I had done in my past no longer are exercises that I can do today because I know that physically I can't do those. So, looking for new things to exercise. New techniques. So, if I wanted to exercise years ago, if I am like okay, let's go running, even though I was smoker, I could run like crazy. I don't know why.

I: What would make it easier for you to overcome those barriers?

P: What would make it easier? Um, finding new techniques of exercising. In other words, if I can't run, what could we do to replace the running? Is it just walking? Is it walking at a better pace? Things like that.

I: So, one of the things we are considering in our exercise program is group classes. How do you feel about exercise classes in general?

P: I have no issue with them.

I: How do you feel.. so do you like.. have you had experience in group classes? Do you like them?

P: Not exercising.. I've never been in a group exercise. You know, I was in, I learned how to disco dance with a group. That was fun.

I: Okay. So, you enjoyed..

P: I enjoyed mingling with people sure.

I: So, how do you feel about exercise classes created especially for patients with kidney disease?

P: It's good! I think it will be exciting. [inaudible] other people's views.

I: Do you have any barriers about participating in this study?

P: No. No.

I: Any transportation or time issues?

P: Well, I will have to see about... because I don't drive. About getting a ride up here for that time. [0:10:01.2] Whenever that time is.

I: Okay. But you will be able to..

P: But the mornings are better for me to get rides than the afternoons. Like I said, I live [MorningHill] so it's harder to get a hard from here to there.

I: So, how should we design these classes to be most convenient and appealing to you?

P: Oh just, again, for me, mornings, mid-mornings is easiest. And uh..

I: Any type of activity?

P: And you start, you know. As anything, you start slow and build yourself up to it?

I: Any type of activities that you want to have in these exercise..?

P: Well, more walking. You know, maybe I don't know if you have any muscle gain.

I: What would you value the most from this exercise?

P: Better health. I don't know what more you would need other than better health. And better shape.

I: How would you judge this exercise program to be successful?

P: For me, for me to walk away knowing that it wasn't a waste of my time. So um, and kept my interest.

I: What is your experience with exercise instructors?

P: None. Not that I can think of.

I: So, if you were attending the exercise class, what could the exercise instructor do to help you be more willing and excited about exercising?

P: Hmm, just be positive. And uh, be understanding of limitations.

I: How helpful is goal setting? For you?

P: For me, very helpful.

I: What things would discourage you from..?

P: Cancelations. And uh, bad instructors.

I: Anything else.

P: Nothing else would discourage me... Or, not being able to do it. Being told that there is nothing we can do for you. That would be discouraging.

I: We are considering using a mobile app for this study. Can you tell me your past or current experience with health apps on your mobile phone?

P: I don't have any.

I: So no.. okay. You haven't used..

P: I have never used an app for health reasons.

I: Any activity tracker besides that you have used?

P: No, never.

I: If we.. will you be able? Are you computer savvy with..? you know.

P: Yeah, somewhat.

I: Somewhat. So, you will be able to download apps?

P: Oh yeah. I can figure out the Valley app.

I: Oh, there is a Valley app, I didn't know that. [laughter]

P: Oh my gosh, that's how you set up all your appointments.

I: Oh! Oh it's like a my health..

P: Yeah, yeah, yeah. I can figure out their app.

[0:14:08.6]

** End of Interview

Audio File Name: 7-27-18_43198-009.mp4

Recording Date: 7/27/2018

Interviewer: LLD

Transcriber: AB

Dates: 8/6/2018

File Name: #7 CKD Interview Stanford (7-25-2018)

**Start of Interview

Study ID 43198-009 Date of Visit: July 27th 2018

I: Okay [Name], can you tell me about a time that you were physically active that you really enjoyed?

P: Uh, around 4 years ago, yeah.

I: Can you tell me what kind of physical activities you did?

P: Basketball like that. Walking.

I: And did you do that every day?

P: Almost every day.

I: What do you think when you hear the word exercise? Can you tell me your thoughts about exercise?

P: It's uh.. being physically fit.

I: Anything else that you want to uh, when you hear exercise, how does it make you feel?

P: Energy and like that, yeah.

I: Anything else?

P: Uh.. physical health. Good.

I: So, tell me about your current exercise now?

P: Usually, when I came here, I just do my work. So, my exercise is my work.

I: Okay, so tell me what you do for work and what kind of exercise it is?

P: Actually.. we, I uh assemble parts, includes sculpting. It is like a machine. So, sometimes I lift the base which is quite heavy. Around 20 pounds. The [chillers] is also around I think 30 pounds like that. But not per regularly. Usually uh, the most uh, maybe 8 times per day I lift, do all this lifting, yeah. And just transferring and like that.

I: So you work. So you're exercise is at work doing kind of lifting and is this a daily um, a daily thing you do every day?

P: Uh, there are some weeks that I do it but there some weeks I just uh, do the [torking?] just uh, making assemblies on the parts. Yeah so, I do not much of lifting on that week. But there are some weeks that I do a lot of lifting.

I: So do you do any exercise after work?

P: No.

I: So the usual amount of time of your physically activities is like 8 hours at work. Okay, um can you describe like when you were physically active, what would you do?

P: Usually, in the morning when I was in the Philippines, usually I wake up early. I would just walk, for like a few miles. Walk and jog. Exercise, stretch and like that. Sometimes in the afternoon I play basketball.

I: And is this something that you did daily?

P: Uh, not really daily but mostly maybe 3 times a week, 4 times a week.

I: Okay. How did your exercise routine change when you were diagnosed with kidney disease?

P: Cause uh, sometimes I feel tired and not.. And I was a little depressed. Yeah.

I: Because you didn't have the energy?

P: Yeah. Yeah. The strength. And uh, when I was. I had uh, swelling in my feet. The first, I thought it was just my previous injury because I had injuries. I thought it was just uh, another injury on my ankles. But uh, the doctor said it was because of my kidney disease. Yeah.

I: Oh, okay. **[0:04:59.4]** So, when you were diagnosed, you um felt tired. No energy.

P: Yeah.

I: Okay. Tell me some of the benefits of exercising.

P: Uh, before of course I feel happy. Because uh, I can see the sweat, my sweating. I'm with my friends. Yeah.

I: Oh, so yeah, it's with your friends. You're doing things with your friends at the same time exercising. Um, what motivates you to become physically active?

P: Before or now?

I: What motivates you? Yeah now, uh before. What motivated you?

P: Of course, because uh I am happy with.. cause I really like to play basketball. So..

I: So, you enjoyed that at the same time.

P: Yeah. But right now, when I usually sometimes play with my kids, I get tired easily. And sometimes, my arms hurt now.

I: Oh, so you get some pain?

P: Yeah, yeah.

I: Were you feeling some of the pain in your shoulders?

P: Yeah and in my back, I think.

I: Um, how important is exercise with patients with kidney disease?

P: Um, for me, it is just, I really don't know but I think it helps motivate them to live a normal life. And uh, I hope it helps, uh it helps prolong my dialysis or improves my health.

I: Has [name] talked to you about exercise and kidney disease management?

P: No, not yet.

I: No, not yet. So nothing, no advice that he has..

P: No, no, no.

I: Okay. So, no advice given. What are some of the things that make it hard for you to exercise? Anything from health?

P: Uh, yeah actually my, sometimes I am tired from work. Like that. And, it's not use to be I don't have.. like right now, I don't have much energy like compared to when I was younger.

I: Anything else that makes it hard for you to exercise?

P: Uh, maybe the time? Like that. And I don't know much, know anyone here in the US.

I: Oh, okay. Your family is here though?

P: Uh, my wife, my family lives in San Francisco. The Daly City. I just go there on weekends sometimes. I just live with my brother.

I: Oh, okay. So, your family is over there. Okay. What would make it easier for you to overcome these barriers?

P: If I am healthy life. Healthy. [laughter]

I: So, being tired from work, what could you do to.. is there anything that would make it easier for you?

P: Um, I don't know. But uh, I think the life here [USA] is very stressful for me. For the.. I think for the.. cause of the change. Yeah.

I: So, when did you arrive here from the Philippines?

P: 2016. Yeah.

I: So, lifestyle changed here in the Philippines. Then being adjusted to.. the United States.

P: Yeah still adjusting.

I: Okay, so one of the things we are considering here is group classes. How do you feel about group classes?

P: No problem.

I: You have no problem with it. So, do you have any likes or dislikes about it?

P: No, no.

I: Okay. Um, so how do you feel about group exercise classes for.. created especially for people with kidney disease?

P: I think it is good because uh, **[0:10:00.1]** I will be more.. I'll know more.

I: And you get to meet..

P: Yeah, yeah.

I: Um, so are there any things that will.. any barriers that will stop you from exercising? Is it like any transportation or time?

P: No, no, no. Except for the.. my work, my work.

I: Okay, so um, time has to be after..

P: After 5:30. Monday to Thursday only I work. But on Fridays I can do.. except if we go overtime. There are times when we go overtime.

I: Okay, so how should we design these classes, that is most appealing and convenient for you? Are there any activities that you like.. that you want to have in the exercise program?

P: I don't know what is best for my health, but I think I just want what is best for me, yeah.

I: And, how about any, the location. What is most convenient for you?

P: Um, as long as it's..

I: Around here.

P: Yeah.

I: So, there is no conflicts.

P: No, no, no... Cause actually, when I have free time and I don't have nothing to do. I drive lyft or uber.

I: Okay, so you do uber.

P: Yeah, sometimes, just sometimes.

I: Olay. Um, what would you value most of this program?

P: My.. of course my wellbeing and my health.

I: Okay, how would you judge if this exercise program is successful?

P: If my health would be improved and my energy will improve.

I: Okay, what is your experience with exercise instructors?

P: Uh, they're good. Uh yeah. Sometimes very demand, like that. Cause, I was uh, when I was in high school, I was a varsity player, so my coach was very strict [laughter]

I: Oh, what did you play?

P: Basketball.

I: Oh okay. So, you have experience working with tough exercise instructors.

P: Yeah. Tough, tough coach yeah.

I: So, if you were attending the exercise group, what could the exercise instructor do to help you be more willing and be excited about exercising?

P: Uh.. I'm not sure. Just be himself I think.

I: Anything else that the instructor can do to help you get motivated?

P: Uh.. as long as there are no insults. Yeah.

I: So.. what would discourage you from exercising?

P: That, that. [Insults]

I: Anything else?

P: Yeah no. I'm good.

I: Okay, so part of this study is we are using the health app. Um, can you tell me if you have experience with health apps in the past. Have you used them before?

P: Uh.. in here, do you have health like, like heartbeat. Sometimes I use it.

I: Which one is that? From the iPhone?

P: From the Samsung.

I: Oh, okay.

P: Samsung health.

I: So, do you use that?

P: Yeah, I just sometimes check my heartbeat, heartbeats like that.

I: What do you like about it? And what you don't like about it.

P: I'm not sure if these or accurate or not [laughter] This one [shows her phone]

I: Okay. I see. Oh that's a really nice uh.. So is that a default in your..?

P: Yeah yeah.

I: It's a free app?

P: Yeah, yeah. It's cause, they have a sensor here. And you just put your hand. Heart rate and there is a stress.

I: Okay, do you have any... Oh, they have a stress one too? That's great. Um, so do you use this daily?

P: No [emphasis] no, no.

I: No okay. So you don't.. besides that, you don't use any other exercise apps?

P: No, no.

I: Okay... so you are familiar with apps and computers.. okay. Okay great, this is great. Thank you so much.

**[0:15:03.7]**

** End of Interview

**Audio File Name: 1-28-2019 #43198-136.m4a**

**Recording Date: 1/28/2019**

**Interviewer: LLD**

**Transcriber: BB**

**Dates: 2/26/2019, 3/2/2019**

**File Name: #8 CKD Interview Stanford (1-28-2019)**

[00:00:00]

**Start of Interview

I: Ok, so I’m gonna go through some questionnaires here, there are no right or wrong answers so just be honest. Everything you say today will be kept completely confidential.

P: Ok

I: Ok? So, in order for us to catch everything, that’s why we’re recording our discussion. Ok?

P: No problem.

I: Ok, so we want to learn a little bit more before we get started. Can you tell me about a time you were physically active that you really enjoyed?

P: Hmm, mean a specific incident?

I: Yah, any time in your life that you were, that you.

P: I can't actually think of any [laughter]. Physical activity is not something I've made a big point of. Uh [pause], I guess when I was, when I first met Alejandra.

I: When was that?

P: Um, 30 years ago, 89.

I: 1989?

P: She got me some sessions with a trainer in a gym. I'd never been to a gym before, or with a trainer, and I kinda enjoyed those.

I: What did you do during those sessions?

P: Well he just showed me how to use different, there were how many what like five or six sessions, uh yeah, so he just showed me how to use the various machines in the gym, and then just set me up with a routine of you know, exercises and taught me what reps were and sets were, stuff that I had no idea at the time.

I: So, what do you think when you hear the word exercise? What comes to mind?

P: Guilt, it's something I should do but don't. Well I go to the gym, although I haven't been in the last couple of weeks, but its uh, the previous few months I was going two to three times a week, up until Christmas. And I actually quite enjoyed going there. I got very cool to the point where I had my phone and my podcasts and the headphones and on the bicycle, expect I was really cool. [Laughter]

I: What is, what were your exercise routine?

P: At the gym?

I: Yah at the gym.

P: It was mostly focused on endurance rather than strength because I was told that's what I should be focusing on. So, I'd always start with like thirty minutes on a stationary bike, and that was quite good because over the time I was doing it I started, you know, I could only do ten minutes or fifteen minutes and had to take a break, and at the lowest level of resistance on the bicycle. And fairly rapidly, I progressed I could do thirty minutes at the highest level of resistance without stopping and pretty much every session I was doing a little more than I did the session before. I find that kind of, uh inspiring is not the right word, but I found it sort of encouraging that I was, I could see progress, you know? So I always did that and there were two or three other machines that I did basically exercised my upper body because I have, you know as you can probably see, I have posture issues which are partly a result of the surgery I had on my lower spine, because of he put in a rod and it fuses all the vertebrae and at the end of the rod there is a lot of stress on the next vertebrae and tend to bend over, that's what I'm doing. So, we're trying to help that by improving the strength of my back muscles, the upper back. To improve posture and then ultimately, I may end up with another back surgery, but hopefully that's a while away. I'm sorry, any other stuff that I do... um, no not really just the gym, the two or three machines that I used, that was about it.

I: So how long, like frequency, like did you go once or twice a week, or?

P: It was two to three times a week.

I: Two to three times a week, ok.

[00:04:59.7]

P: I didn't have a set schedule or anything.

I: And where did you work, where is your gym at?

P: It's at the Centennial Recreation Center in Morgan Hill.

I: So, have you been doing this recently? Your exercise?

P: Um, apart from the last two or three weeks, yeah, but basically at Christmas I sort of stopped, but I need to get back into it. And I will, I've been once this month which is not very good but, I feel guilty about it, so I need to get back into it.

I: How did your exercise routine change when you were diagnosed with kidney disease?

P: It hasn't.

I: It hasn't changed?

P: No.

I: What are some of the benefits of exercising?

P: Well, I'm thinking hot and just muscles and just general energy level.

I: Any personal benefits?

P: My wife stops nagging me. [Laughter] She's been trying to get me to go to the gym for a very long time.

I: What motivates you to be physically active?

P: Um, I want to improve from where I am now so I'm not taking my walker with me and you know, my health, even though I didn't exercise, much at all, up until about three years ago, I was doing pretty good, apart from being diabetic, I was fine, you know? But in the last three years, I had a toe amputated, I had my spine surgery, I had the kidney diagnosis, I also have a heart problem, turns out. My body's kinda falling apart at the moment, so hopefully exercise will help to at least slow down that process if not reverse it a little bit, that's what I'd like to think.

I: What do you think is important, why exercise is important for patients with kidney disease?

P: Well, because you don't want to progress to dialysis basically. I mean there's no cure for kidney disease that I'm aware of. If I can at least slow the progress, then that's better.

I: Has your doctor talked to you about any other, any exercise and kidney disease management?

P: No.

I: No? Who's your doctor?

P: My primary care physician is [name].

I: Ok, but your nephrologist?

P: Oh, nephrologist is [name].

I: Oh, ok. So, she hasn't recommended any exercise or kidney disease management?

P: No.

I: No, ok. So, what are some of the things that make it hard for you to exercise?

P: Um, it’s never really been a part of my routine, up until six months ago, when I finally got to go into the gym, with a little encouragement from my wife, who exercises daily and has done for the last thirty years, you know?

I: Are there any health barriers? Time, or?

P: No, I'm retired.

I: Ok, are you feeling unmotivated?

P: Um, yeah but that's the kind of general feeling I've had for a couple of years now, or more.

I: So, what would make it easier for you to overcome your barriers?

P: I think just being able to track progress to see that I'm actually improving. Like I said, I find it very encouraging when I was using the bike, cause every time I went, I could see I was pedaling further, or I was pedaling faster, or I was pedaling at a higher resistance rate. And to me that was very encouraging because I could see an improvement every week or every time I went there actually.

[00:10:05.1]

I: So, one of the things that we're doing in this study is [catching] exercise program in group classes. How do you feel about group classes in general?

P: Um, I've taken them, I've given them, so yeah.

I: So, you have experience?

P: Yeah.

I: Is there anything you like or dislike about having group classes?

P: No.

I: No.

P: Nothing I can think of, no.

I: So how do you feel about exercise classes created for patients with kidney disease?

P: I think it's great. You said there is no cure right now, so anything we can do to slow or stop the progress is good.

I: Do you have any barriers to participating in group classes?

P: Other than just getting here, I can get here, I mean sometimes my wife brings me. My eyesight's not as good as it used to be so I'm kind of uncomfortable driving around San Jose these days. But, there's VTA access that I use, that works. What else? Oh yah, I use VTA access or I can just flat take the bus. Believe it or not, it takes about the same time as the car. Because after a while for them, picking other people up and dropping them off on the way here, they inevitably get me here early. Always, always early. But, you know, I can also take, there's a bus at the end of the street. I mean literally, you know, a hundred feet from the front door, I'd catch the bus. I can get a train, got on another bus and I'm here, in about the same time, literally about an hour and twenty minutes. Which is really not too bad.

I: So how should we design these classes, to be most convenient and appealing to you?

P: I don't know. I think you already have a schedule three times a week.

I: Are there things that, that is more appealing to you like types of activities and like activities that you um, enjoy doing?

P: No, not really. There's no activities that I, I haven't participated in sports, ever. There's no kind of specific sporting activity.

I: How about like location and time?

P: Well if you had them in Morgan Hill that would be really nice. Times, it doesn't really matter, middle of the days is good because then I can avoid the rush hour in the mornings getting here and avoid the rush hour in the afternoon getting home. Because the traffic going down, up and down to Morgan Hill is pretty horrendous.

I: So, what would you value most about this exercise program?

P: Hopefully slowing down the progress of my kidney disease. It's important, you know? That's it.

I: How would you judge if the exercise program was successful?

P: I used to be a management consultant in a previous life, so you live and die by numbers, you know? It would be nice to see progress numerically or graphically.

I: With your individual progress?

P: Whether it's with my [GFR] or whatever else.

I: So, you find it if your lab results are improving.

P: Yah, and if I see Dr. Name and she tells me "Hey, you're doing great", you know, then I'll be good.

I: So personal success would, with the study.

P: Yah, I like to see progress and someone independent from the study saying.

I: What about the program? Anything that you would like, how would you judge if our program is successful?

[00:15:03.1]

P: Well it would be interesting to compare my progress with other similar people's progress. I'm not sure how comparable we are but it would be, you know, where am I in the pack, am I you know, a superstar or am I way at the back of the pack, I don't know? It'd be nice to know that.

I: What is your experience with exercise instructors?

P: Um, I had the one thirty years ago that my wife paid for, he was a nice guy. Since then, nothing up until I got discharged from the hospital after my surgery last year, and I was in a nursing home for about seven weeks. I was in bed for the first two, but after that I had physiotherapy. Occupational therapists first, just to get me walking again. And then physical therapist, that I've actually stayed with, still, and I still see her about, well once a week, but no, once a month. I was seeing her several times a week when I was in the nursing home, you know, and she was very good. And then when I was discharged, I got home physical therapy from a different company, just because of the insurance. And that was fine, and then after that I became an outpatient again back at the nursing home where I'd been before. And I was with the same physiotherapist and that was good because I just felt like she knew me, she'd seen my progress, and that worked pretty well, still works pretty well. We've been slowing it down a bit with, actually last time I saw her was I think December. And we said we'll do it once a month, and this last week I was again at Stanford with the neurosurgeon who may be the one doing the surgery on my upper back next, and he recommended physiotherapy once a week. So, I'm gonna tell my physiotherapist next time I see her, we need to do this more regularly. And I think also focus a bit more because with her I've been focusing mostly on, she'd recommended endurance and that's what you really need to work on because I have no endurance. And she said strength was not maybe that big of an issue for me because as I was bent over, if she'd try and strengthen your muscles now while you’re in this kind of posture, you'll be in that posture permanently. But the neurosurgeon said I should be working on strength as well, especially my back muscles and also my leg muscles. So, I have to talk to her about that, see her more regularly.

I: So, if you were attending an exercise class, what could the exercise instructor do to help you be more willing and excited about exercise?

P: I'm thinking you have to connect with people, if you connect with the instructor and that's great, but if it's somebody you don't connect with then it's not so much fun. When I was learning to fly, intimate is not the right word, but it’s a very close relationship because there's two of you in the plane you know? And if you don't build a rapport with your instructor, even though the guy is telling you stuff, it's just not enjoyable. I've had an instructor who was, I liked being with very much, and I've had instructors who were not really much fun, you know? Yeah, you gotta be able to connect.

I: To connect, yeah. How helpful is goalsetting for you when you exercise?

P: To be honest it doesn't do a lot, I like to see my own progress but to me setting a goal is almost like take a random number and then try and reach it. I just, in my former life as a management consultant, I saw so many goals and strategies and all this other stuff to meet the goals and it turned out the goal was simply a random number that some high up executive thought of and it just was nonsense, didn't reflect reality. So, something that reflects reality. Here's my current status, here's what I can achieve, and two weeks from now here's what I can achieve and if it's better, then great, so I like to see, you know, the progress rather than looking for some goal out there. That just doesn't do anything for me.

I: What things would discourage you when exercising?

[00:19:53.3]

P: The reverse I guess, not having an instructor I felt comfortable with. Or someone who was, I don't want to use the word smart but, someone who has read a lot of books in goalsetting and strategy and doesn't have a clue. A well-read kid or something like that is not something I would connect with.

I: So, we're considering using a mobile app for this part of the program. Can you tell me about the past and current experiences of you using a health app on your mobile phone?

P: Well the Apple phone, the iPhone comes with a health app, which I looked at a couple of times and I didn't, quite honestly, have a lot of interest in.

I: So that's the only thing you have?

P: Well I got my Fitbit app, but I only got that on Saturday evening, so I had it two days and I've had a look at it. My experience so far has not been good for a number of reasons. One, it's difficult to read. It was poorly designed, basically. And I'm sorry it’s my management consulting hat on again but, I used to design user interfaces and it's one of the things I did, and I look at that, and I look at basics like a light grey font on an off-white background, I'm sorry that's not exactly visible, you know? That kind of stuff, to me that's basic.

I: Is there things that you like about it? About the health app you used?

P: It's too early to say.

I: So, you just received that on Saturday? So how would we, with our study, would you probably have to remove that?

P: I'd keep it on the other wrist yeah. I'll be comparing the two, you know, heart rate, number of steps walked, the stuff I've always wondered about, how this stuff calculates. And it'd be interesting to compare the two, and that's just my tacky hat on.

I: So, you are familiar with you know, health apps and downloading and syncing and data. Ok, because that's part of our study, we'll have to have you daily sync your data from the Garmin.

P: Yeah, I've designed and developed websites. I even developed a language app for my wife, police officers so they can, oh I've given out a ticket and look it up and now here's all the phrases you need and here's, that kind of stuff. It took a long time.

I: Ok, well this is great. You've given us a lot of information for this study. Is there any questions that you have?

P: No, I'm looking forward to it. I hope it's gonna help you know?

I: Ok so we're done with the part of the questionnaire.

** End of Interview
